# Solid-state ^17^O NMR study of α-d-glucose: exploring new frontiers in isotopic labeling, sensitivity enhancement, and NMR crystallography†

**DOI:** 10.1039/d1sc06060k

**Published:** 2022-01-03

**Authors:** Jiahui Shen, Victor Terskikh, Jochem Struppe, Alia Hassan, Martine Monette, Ivan Hung, Zhehong Gan, Andreas Brinkmann, Gang Wu

**Affiliations:** Department of Chemistry, Queen's University 90 Bader Lane Kingston Ontario K7L 3N6 Canada wugang@queensu.ca; Metrology, National Research Council Canada Ottawa Ontario K1A 0R6 Canada; Bruker Biospin Corporation 15 Fortune Drive, Billerica MA 01821 USA; Bruker Switzerland AG Fällanden Switzerland; Bruker Biospin Ltd. 2800 High Point Drive, Suite 206 Milton Ontario L9T 6P4 Canada; National High Magnetic Field Laboratory 1800 East Paul Dirac Drive Tallahassee Florida 32310 USA

## Abstract

We report synthesis and solid-state ^17^O NMR characterization of α-d-glucose for which all six oxygen atoms are site-specifically ^17^O-labeled. Solid-state ^17^O NMR spectra were recorded for α-d-glucose/NaCl/H_2_O (2/1/1) cocrystals under static and magic-angle-spinning (MAS) conditions at five moderate, high, and ultrahigh magnetic fields: 14.1, 16.4, 18.8, 21.1, and 35.2 T. Complete ^17^O chemical shift (CS) and quadrupolar coupling (QC) tensors were determined for each of the six oxygen-containing functional groups in α-d-glucose. Paramagnetic Cu(ii) doping was found to significantly shorten the spin–lattice relaxation times for both ^1^H and ^17^O nuclei in these compounds. A combination of the paramagnetic Cu(ii) doping, new CPMAS CryoProbe technology, and apodization weighted sampling led to a sensitivity boost for solid-state ^17^O NMR by a factor of 6–8, which made it possible to acquire high-quality 2D ^17^O multiple-quantum (MQ) MAS spectra for carbohydrate compounds. The unprecedented spectral resolution offered by 2D ^17^O MQMAS spectra permitted detection of a key structural difference for a single hydrogen bond between two types of crystallographically distinct α-d-glucose molecules. This work represents the first case where all oxygen-containing functional groups in a carbohydrate molecule are site-specifically ^17^O-labeled and fully characterized by solid-state ^17^O NMR. Gauge Including Projector Augmented Waves (GIPAW) DFT calculations were performed to aid ^17^O and ^13^C NMR signal assignments for a complex crystal structure where there are six crystallographically distinct α-d-glucose molecules in the asymmetric unit.

## Abstract 2

We report the first “total synthesis” of ^17^O-labeled d-glucose and its solid-state ^17^O NMR characterization with unprecedented sensitivity and resolution.

## Graphical abstract

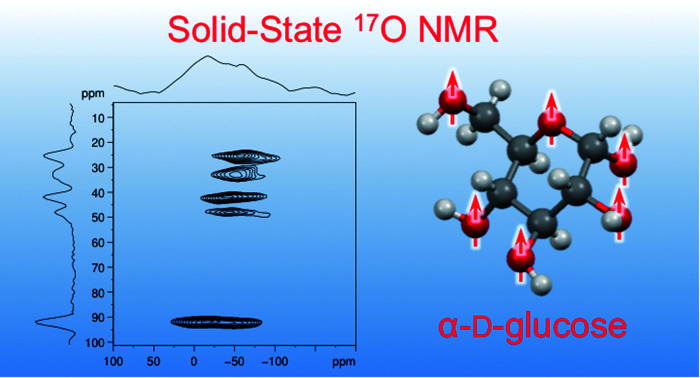

## Introduction

The element of oxygen is a key constituent of organic and biological molecules. Oxygen-containing functional groups are often directly involved in chemical reactions including biological transformation such as enzyme catalysis. While NMR spectroscopy is a powerful technique for structural elucidation of organic and biological molecules, most NMR studies are based on detection of signals from hydrogen, carbon, nitrogen, and phosphorus atoms. While it is highly desirable to add oxygen to the list of nuclear probes available for NMR studies, two major obstacles have made it difficult to characterize NMR signals from oxygen atoms. First, the NMR-active oxygen isotope, ^17^O, has an exceedingly low natural abundance (0.037%). Thus, it is usually necessary to prepare ^17^O-enriched molecular systems in order to boost NMR detectability. This ^17^O-labeling process can be a difficult task. Second, ^17^O has a quadrupolar nucleus (*I* = 5/2), which often gives rise to significantly broader NMR signals than those commonly encountered from other more NMR-friendly spin-1/2 nuclei such as ^1^H, ^13^C and ^15^N. This quadrupole line broadening is a major roadblock to ^17^O NMR applications in terms of spectral resolution. Over the last two decades, however, significant progress has been made in demonstrating ^17^O NMR as a viable tool to study organic and biological molecules in both solution and the solid state.^1–7^ For ^17^O NMR studies of biological molecules, in particular, some important developments have occurred in recent years. Zhu *et al.*^8^ showed that it is possible to obtain solid-state ^17^O NMR spectra from protein–ligand complexes where the ligand molecules are site-specifically ^17^O-labeled. Tang *et al.*^9^ applied this approach to study hydrogen-bonding interactions around the “oxyanion hole” in several acyl-enzymes. Zhu *et al.*^10,11^ demonstrated a technique known as quadrupole-central-transition (QCT) NMR in obtaining high-resolution ^17^O NMR spectra for biological macromolecules undergoing slow tumbling motion in aqueous solution. Young *et al.*^12^ applied the ^17^O QCT method to monitor the formation of enzymatic intermediates of tryptophan synthase under active catalysis. Recently, Paulino *et al.*^13^ reported a comprehensive ^17^O solid-state NMR study of the water–carbonyl interactions in gramicidin A ion channel. The latest advancement in the field was the work by Lin *et al.*^14^ where they demonstrated a general approach to incorporate ^17^O isotopes into recombinant proteins and reported solid-state ^17^O NMR spectra for yeast ubiquitin.

In addition to the abovementioned new applications, there have also been recent developments in solid-state ^17^O NMR methodology. One particular area of interest is concerned with heteronuclear correlation solid-state NMR spectroscopy between ^17^O and other nuclei such as ^1^H, ^13^C, and ^15^N.^15–19^ For example, Hung *et al.*^19^ reported a new 3D D-RINEPT/DARR OCC experiment where overlapping ^17^O NMR signals can be completely separated in the ^13^C dimension. Another highly promising direction is to use dynamic nuclear polarization (DNP) to enhance ^17^O NMR signals for organic and biological molecules.^20–23^ Currently, most DNP-enhanced ^17^O NMR studies were performed at low or moderate magnetic fields (≤14.1 T) to study inorganic materials; it would be highly beneficial for the study of organic and biological molecules if DNP for ^17^O becomes feasible at higher magnetic fields.^24^

**Scheme 1:**
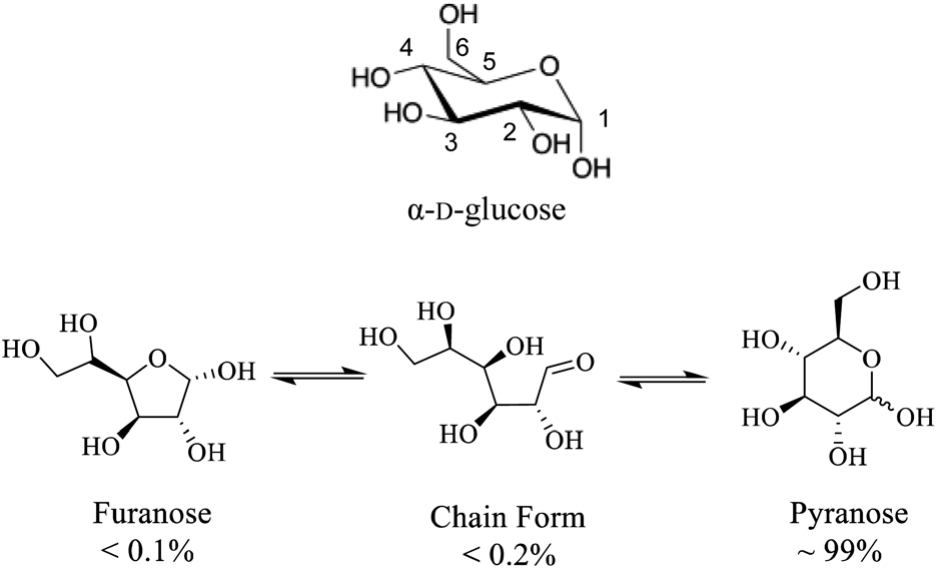
(Top) Molecular structure of α-d-glucose where carbon atoms are numbered. (Bottom) different d-glucose tautomers present in aqueous solution.

While fundamental ^17^O NMR data on chemical shift (CS) and electric-field-gradient (EFG) tensors have been reported for many oxygen-containing organic functional groups, there are still many unexplored classes of organic compounds for which little is known about their ^17^O NMR tensor properties. One notable example is concerned with carbohydrates. Carbohydrates are an important class of oxygen-rich organic molecules of biological significance. However, solid-state ^17^O NMR studies dealing with carbohydrate molecules are very rare in the literature. Sefzik *et al.*^25^ reported the first solid-state ^17^O NMR study of several protected carbohydrate compounds. Yamada *et al.*^26^ obtained the solid-state ^17^O NMR signal for the O6 atom of d-glucosamine. More recently, Hung *et al.*^19^ reported 2D and 3D ^13^C–^17^O heteronuclear correlation solid-state NMR spectra of [1-^13^C,^17^O]-α/β-d-glucose. Also relevant are two ^17^O QCT NMR studies by Shen *et al.*^27^ and by Gan *et al.*^28^ where ^17^O-labeled d-glucose samples were examined with the aid of high magnetic fields. One major challenge in solid-state ^17^O NMR studies of carbohydrates is the synthesis of ^17^O-labeled target compounds. To further explore synthetic procedures and solid-state ^17^O NMR for unprotected carbohydrate compounds, we selected d-glucose as an initial target (Scheme 1). In this work, we report synthesis of a total of six site-specifically ^17^O-labeled d-glucose compounds and their full solid-state ^17^O NMR characterization. For the latter part, because crystallization of d-glucose into a pure anomeric form (α or β) often encounters low yields, we decided to prepare all solid samples of d-glucose in the form of a d-glucose/NaCl/H_2_O (2/1/1) cocrystal. This cocrystal is known to contain exclusively α-d-glucose and can be readily prepared in crystalline form with near 100% yields.^29–31^ Throughout this work, we will use “α-d-glucose” as a shorthand name for the α-d-glucose/NaCl/H_2_O (2/1/1) cocrystal.

Another objective of the present work is to demonstrate utilization of the current state-of-the-art solid-state ^17^O NMR technologies achieving unprecedented sensitivity and spectral resolution for organic and biological molecules. To this end, we explore the following three areas. First, we perform solid-state ^17^O NMR at multiple magnetic fields including an ultrahigh magnetic field of 35.2 T.^28^ Second, we investigate the effect of paramagnetic doping in shortening spin-relaxation times for ^17^O nuclei so that fast data acquisition might be possible. Third, we test the sensitivity enhancement for solid-state ^17^O NMR applications using a new CPMAS CryoProbe.^32^

## Experimental section

### Synthesis of sitespecifically ^17^O-labeled d-glucose compounds

**Scheme 2:**
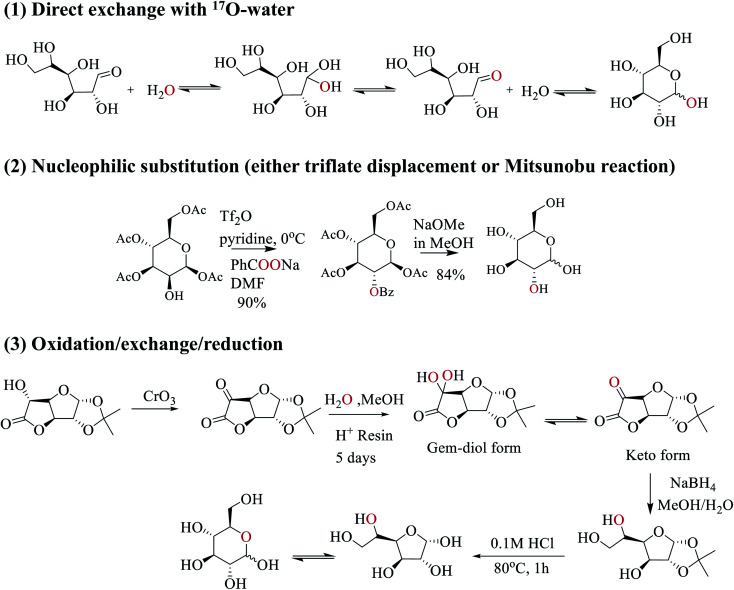
Three synthetic strategies used in this work to prepare site-specifically ^17^O-labeled d-glucose.

In this work, we employed three strategies to synthesize site-specifically ^17^O-labeled d-glucose compounds; see Scheme 2. First, the anomeric O1 atom in d-glucose can be readily ^17^O-labeled by a simple exchange with ^17^O-water.^33–35^ This exchange occurs through the hydration/dehydration process of the aldehyde functional group in the open chain glucose tautomer. Second, for both primary and secondary hydroxyl groups (O2, O3, O4, O6), ^17^O isotopes can be incorporated into glucose by S_N_2 nucleophilic substitution (*via* either triflate displacement route or Mitsunobu reaction) from appropriate starting epimers.^36^ In this case, either sodium [1,2-^17^O_2_]benzoate (triflate displacement reaction) or [1,2-^17^O_2_]benzoic acid (Mitsunobu reaction) can be used as the source of ^17^O. For example, as shown in Scheme 2, in pyridine at 0 °C, the O2 atom of [1,3,4,6-acetyl]-d-mannose is first functionalized with triflate anhydride, followed by the triflate displacement reaction in DMF with sodium [1,2-^17^O_2_]benzoate. Subsequent removal of the protecting groups gives [2-^17^O]-d-glucose. Third, for ^17^O-labeling of the O5 atom, we utilize a combined oxidation/exchange/reduction method starting from 1,2-*O*-isopropylidene-d-glucofuranurono-6,3-lactone as illustrated in Scheme 2. After oxidation of the OH group by chromium trioxide,^37^^17^O-labels are introduced onto the keto group from ^17^O-water *via* an acid-catalyzed hydration/dehydration process (or keto/gem-diol exchange). Then, reduction with NaBH_4_ converts the keto group back to the hydroxyl group.^38^ Finally, removal of protecting groups allows the furanose/pyranose equilibrium to occur, producing [5-^17^O]-d-glucose.^39^ Full details of the synthetic procedures and compound characterization are provided in the ESI.†

### Preparation of solid samples

As mentioned above, because crystallization of d-glucose into the pure α (or β) form is often associated with low yields, we prepared all solid samples of ^17^O-labeled d-glucose as a d-glucose/NaCl/H_2_O (2/1/1) cocrystal where all d-glucose molecules are in the α-form.^29^ The d-glucose/NaCl/H_2_O (2/1/1) cocrystal was readily prepared by adding solid NaCl to aqueous solution of d-glucose followed by lyophilization. A solid sample was prepared as an equal molar mixture of [3-^17^O]-d-glucose, [5-^17^O]-d-glucose, and [6-^17^O]-d-glucose. This sample was denoted as [3/5/6-^17^O]-α-d-glucose in this work. Because the three compounds may have different levels of ^17^O-enrichment, the mixing process was monitored by solution ^17^O NMR; see ESI.† As a result, the level ^17^O enrichment in this [3/5/6-^17^O]-α-d-glucose sample was only about 10%. The integrity of all solid samples was checked by acquiring solid-state ^13^C CPMAS NMR spectra; all spectra are provided in ESI. Solid samples with paramagnetic Cu(ii) dopants were prepared in the following fashion. To 2 mL of H_2_O was first added 15 mg of solid Na_2_[Cu(EDTA)_2_] to give a clear blue solution, followed by addition of 150 mg d-glucose/NaCl/H_2_O (2/1/1) cocrystal. The solution turned greenish when solids were fully dissolved. The solution was then dried under a stream of N_2_ until it became a syrup. Addition of 2 mL of absolute ethanol induced crystallization. After removal of the supernatant, solids were dried in air. Cu(ii)-doped solid samples displayed the same solid-state ^13^C CPMAS NMR spectra as regular d-glucose/NaCl/H_2_O cocrystals; see ESI.†

### Solid-state NMR

Solid-state ^17^O and ^13^C CPMAS NMR data at 14.1 T were collected on a Bruker Avance-600 NMR spectrometer at Queen's University. For static ^17^O NMR experiments, a Bruker 4 mm HX MAS probe was used. The 90° pulse width for the ^17^O central-transition (CT) was 2.0 μs. ^1^H decoupling with 60 kHz rf field was applied during data acquisition in the static experiments. Solid-state ^17^O NMR experiments at 21.1 T were performed on a Bruker Avance-II 900 NMR spectrometer at the National Ultrahigh Field NMR Facility for Solids (Ottawa, Ontario, Canada). A Hahn-echo sequence was used for acquiring solid-state ^17^O NMR spectra under both MAS and static conditions to eliminate probe ringing artifacts. For MAS experiments, a 3.2 mm Bruker HX MAS probe was used where the effective 90° pulse width for the ^17^O CT was 1.0 μs. For static experiments, a homebuilt 5 mm solenoid probe was used with powder samples packed into 5 mm Teflon tubes to reduce background signals. On this solenoid probe, the 90° pulse width for the ^17^O CT was 2.0 μs. ^1^H decoupling with 75 kHz rf field was applied during data acquisition. A liquid H_2_O sample was used for both rf power calibration and ^17^O chemical shift referencing (*δ* = 0 ppm). All spectral simulations were performed with DMfit.^40^

Solid-state ^17^O NMR experiments at 35.2 T were carried out on the 36 T series-connected hybrid (SCH) magnet^28^ at the National High Magnetic Field Laboratory (NHMFL, Tallahassee, Florida, USA) with a Bruker Avance NEO console. A single-resonance 3.2 mm MAS probe with an external field regulation circuit designed and constructed at the NHMFL was used with pencil-type ZrO_2_ rotors spinning at a MAS frequency of 16 kHz. A Hahn-echo sequence was used with 5 and 10 μs pulses (with 16.7 kHz rf field) and a recycle delay of 0.1 s.

Solid-state ^17^O and ^13^C NMR experiments were also performed on a Bruker NEO-800 (18.8 T) at the Bruker application lab (Fällanden, Switzerland) with a broadband 3.2 mm CPMAS CryoProbe. The sample spinning was 15 kHz. The ^17^O rf field was about 64 kHz, which gave an effective 90° pulse of 1.3 μs for the CT. The ^1^H decoupling field was 83 kHz. An apodization weighted sampling (AWS) scheme^41^ was used for collecting 2D ^17^O shifted-echo 3QMAS data. For the ^13^C refocused INADEQUATE experiment, the ^13^C 90° pulse was 5.0 μs. The spectral width in the F_1_ dimension was 7.5 kHz. A frequency swept TPPM ^1^H decoupling (83 kHz) scheme was applied during data acquisition.

### Computational details

All quantum chemical calculations were performed using the CASTEP code^42^ (version 2019) together with BIOVIA's Materials Studio. CASTEP employs DFT using the plane-wave pseudopotential approach. The generalized gradient approximation with either the Perdew–Burke–Ernzerhof^43^ or revised Perdew–Burke–Ernzerhof (rPBE)^44^ exchange correlation functionals was chosen. First, geometry optimization was performed employing the Broyden–Fletcher–Goldfarb–Shanno (BFGS) algorithm together with OTFG on-the-fly ultrasoft pseudopotentials (version 2017R2), a cut-off energy of 598.7 eV and a *k*-point grid with a maximum separation of 0.071 Å^−1^. We also tested the treatment of dispersion interactions by using the two-body force-field method of Grimme (D2) (ref. 45) with a re-parameterized damping function (s6 = 1.0; *d* = 3.25 or *d* = 5.0)^46,47^ in geometry optimizations. Subsequently, the NMR parameters were calculated using the Gauge Including Projector Augmented Waves (GIPAW) method implemented in the NMR module of CASTEP.^48–50^ In this work, a total of four sets of GIPAW DFT computations were performed and they are denoted as: (1) PBE, (2) rPBE, (3) rPBE-D2 (*d* = 3.25), and (4) rPBE-D2 (*d* = 5.0). However, because these four methods produced essentially the same results, we will focus on the results obtained with the PBE method and report the complete results from all four methods in the ESI.†

## Results and discussion

### Determination of ^17^O NMR tensors in α-d-glucose

**Fig. 1:**
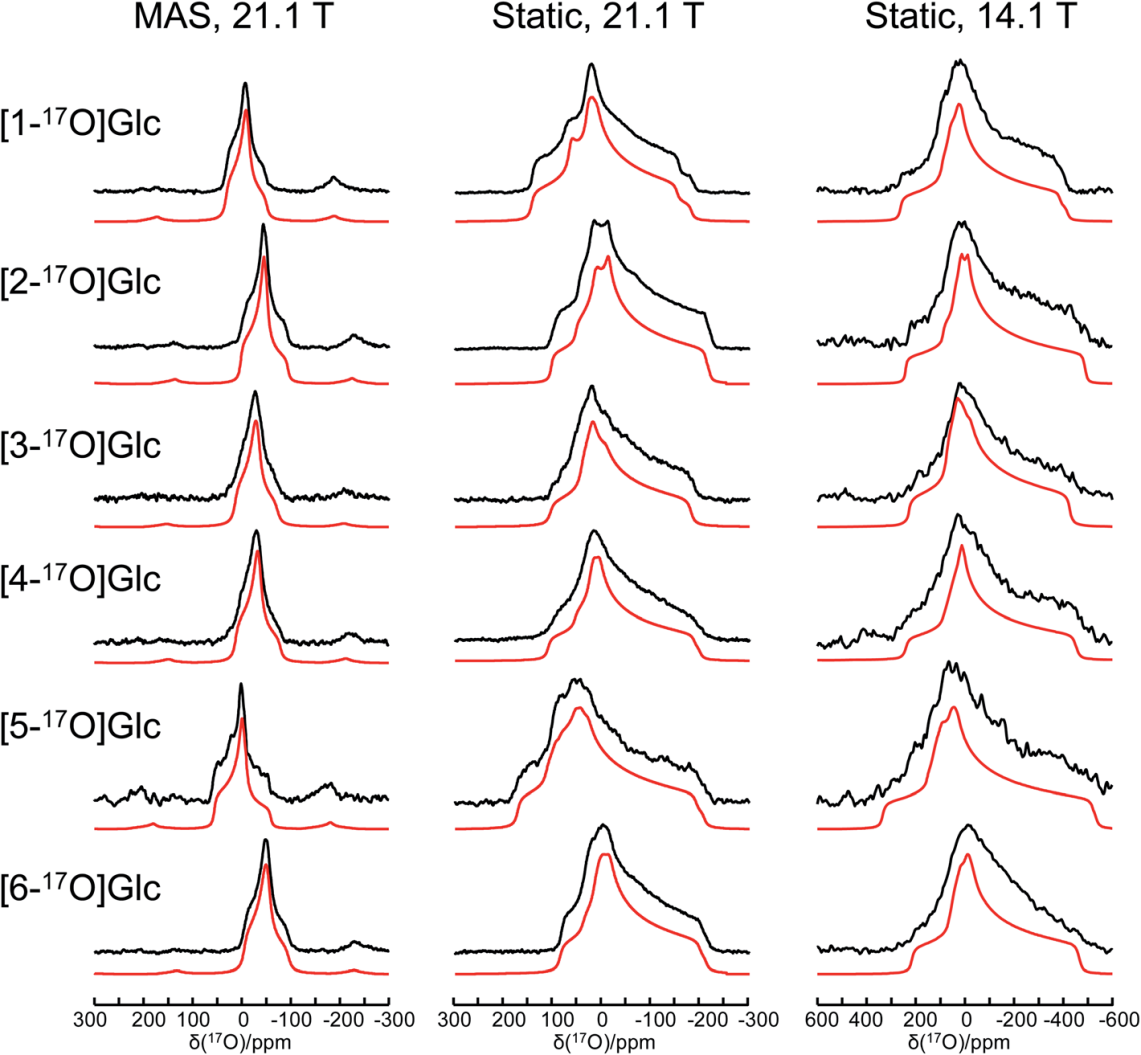
Experimental (black traces) and simulated (red traces) solid-state ^17^O NMR spectra for a total of six site-specifically ^17^O-labeled α-d-glucose compounds under the MAS (22 kHz sample spinning) and static conditions at 21.1 and 14.1 T. The ^17^O NMR parameters used in the spectral simulations are summarized in Table 1. The same set of parameters were used for each compound to simulate spectra obtained at two magnetic fields. Detailed acquisition parameters are given in the ESI.†

Fig. 1 shows the solid-state ^17^O NMR spectra obtained for all six site-specifically ^17^O-labeled d-glucose compounds. In each ^17^O MAS NMR spectrum, a well-defined powder line shape was observed, which is known to arise from the second-order quadrupole interaction. In general, second-order quadrupole interactions are inversely proportional to the applied magnetic field. However, as seen from Fig. 1, even at 21.1 T, second-order quadrupole interactions cause a line broadening on the order of 100 ppm. This is because the oxygen-containing functional groups in d-glucose (hydroxyl and ether groups) are known to experience rather large ^17^O nuclear quadrupole interactions. It is also immediately clear that the relatively small ^17^O chemical shift variations among the six oxygen-containing groups in d-glucose can be easily obscured by such second-order quadrupole broadenings (*vide infra*). In each case, an analysis of the observed powder line shape obtained under MAS conditions allowed us to obtain three ^17^O NMR parameters: *δ*_iso_, *C*_Q_, and *η*_Q_. Complete experimental results are listed in Table 1.

**Table 1:** Experimental ^17^O NMR tensor parameters obtained for α-d-glucose from a spectral analysis of data presented in Fig. 1. The uncertainties in experimental values of *δ*_iso_, *δ*_ii_ (*i* = 1, 2, 3), *C*_Q_, and *η*_Q_ are estimated to be ±2 ppm, ±10 ppm, ±0.2 MHz, and ±0.2, respectively

Atom	*δ*_iso_/ppm	*δ*_11_/ppm	*δ*_22_/ppm	*δ*_33_/ppm	∣*C*_Q_∣/MHz	*η*_Q_
O1	32	72	22	2	8.4	1.0
O2	2	27	−6	−12	9.1	1.0
O3	14	34	12	−5	8.8	0.9
O4	13	33	6	−2	8.9	1.0
O5	56	96	56	16	9.9	1.0
O6	−5	20	−5	−30	9.0	0.9

When the solid-state ^17^O NMR experiments are performed for stationary (non-spinning) powder samples, even broader powder line shapes are observed, as also seen from Fig. 1. At 14.1 T, each static powder line shape spans about 700 ppm, which is reduced to roughly 300 ppm at 21.1 T. This is because now both ^17^O CS and QC tensors contribute to the static powder line shape. The interplay between the two NMR tensors is responsible for the observed field dependence of the static ^17^O NMR spectra. From an analysis of these static powder line shapes, we were able to obtain the principal components of the ^17^O CS tensor and their relative orientations with respect to the ^17^O QC tensor. All experimental ^17^O NMR tensor parameters determined for the six oxygen atoms in α-d-glucose are summarized in Table 1. In general, the values of |*C*_Q_(^17^O)| found in α-d-glucose are about 8–10 MHz with *η*_Q_ close to 1. These parameters are similar to those previously reported for protected carbohydrate compounds,^25^d-glucosamine,^26^ and several other related functional groups such as hemiacetal/hemiketal,^51,52^ gem-diol,^53^ hydroxyl,^54^ and phenolic groups.^55–57^ Because the six oxygen-containing functional groups in α-d-glucose are very similar, their ^17^O isotropic chemical shifts, *δ*_iso_(^17^O), are found within a small range of 60 ppm. Nonetheless, there is a general trend in the observed *δ*_iso_(^17^O) values: O5 (C–O–C part of a cyclic hemiacetal) > O1 (C–OH part of a cyclic hemiacetal) > O2, O3, O4 (secondary alcohol groups) > O6 (a primary alcohol group). These agree with previous solution ^17^O NMR studies^58,59^ as well as with our own measurements for the ^17^O-labeled d-glucose compounds in aqueous solution (see ESI†). Compared with the ^17^O NMR parameters found for crystalline hydrates,^60–64^ the values of |*C*_Q_(^17^O)| for the alcohol and ether groups in α-d-glucose are somewhat larger, but the spans of the ^17^O CS tensors are comparable.

**Fig. 2:**
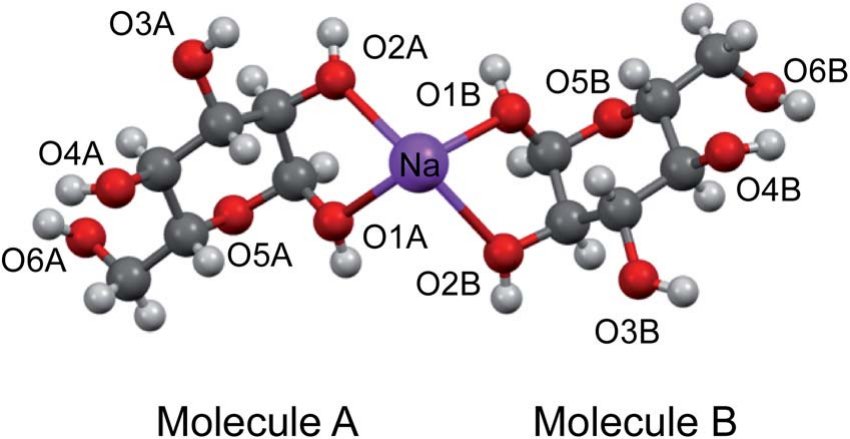
Partial crystal structure of d-glucose/NaCl/H_2_O (2/1/1) cocrystal^29^ displaying one of the three Na^+^-chelated glucose “dimers” in the asymmetric unit. While the two α-d-glucose molecules within each dimer, A and B, are crystallographically distinct, they are nonetheless related by an approximate two-fold axis perpendicular to the page plane. The two axial ligands to complete the octahedron geometry around the Na^+^ ion are O4 atoms from neighboring glucose “dimers”, but are omitted for clarity.

**Fig. 3:**
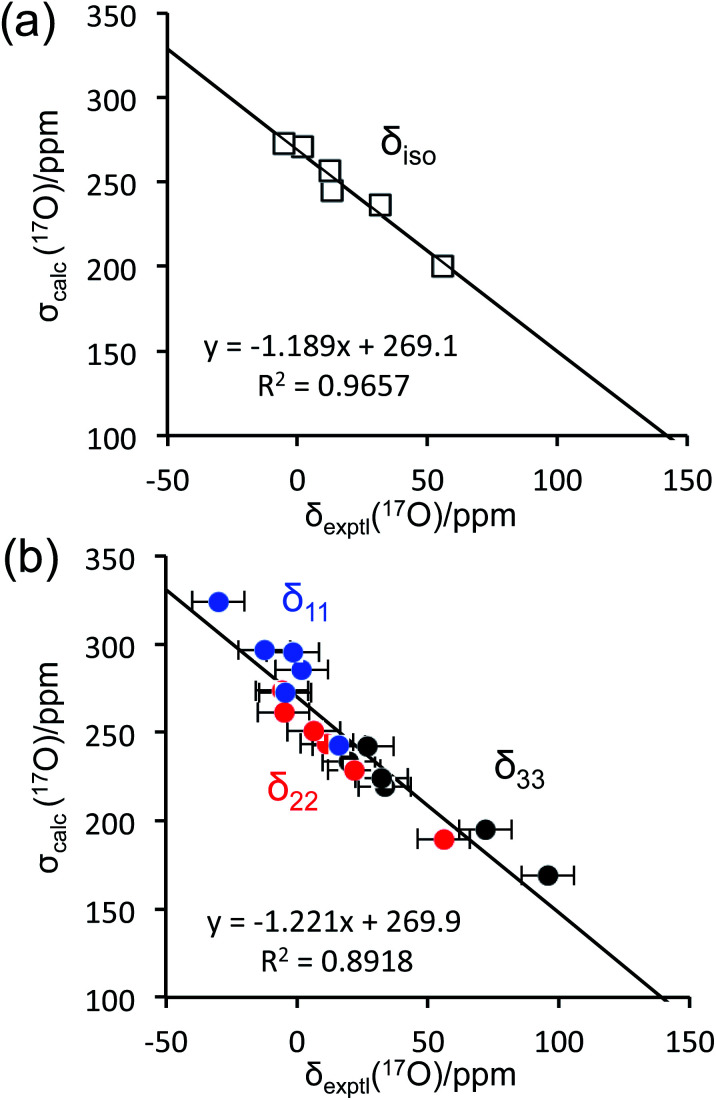
Comparison between experimental ^17^O chemical shifts (*δ*) and GIPAW DFT computed magnetic shielding values (*σ*) for the α-d-glucose/NaCl/H_2_O cocrystal: (a) isotropic values; (b) principal CS tensor components. The root mean square errors (RMSE) are: (a), 4.5 ppm; (b), 12.9 ppm.

To aid the interpretation of experimentally determined ^17^O NMR tensor parameters, we performed extensive GIPAW DFT computations. As mentioned earlier, the choice of making d-glucose/NaCl/H_2_O cocrystal for solid-state ^17^O NMR experiments was based on the considerations for having a pure anomeric form and easy preparation of crystalline samples. Now this turns into a computational challenge, because the d-glucose/NaCl/H_2_O cocrystal has a very large unit cell (trigonal space group *P*3_1_, *a* = 16.836 Å, *c* = 17.013 Å, *V* = 4176 Å^3^, *Z* = 9) that contains six crystallographically distinct glucose molecules in the asymmetric unit.^29^ Careful examination of the crystal structure reveals that the six crystallographically unique d-glucose molecules form three “dimers” *via* Na^+^ chelation with the O1 and O2 atoms, as depicted in Fig. 2. Furthermore, the asymmetric unit contains three water molecules, each involved in hydrogen bonding with both two symmetry-related d-glucose molecules and one Cl^−^ ion. In the original crystal structure,^29^ one of the water molecules was missing a single hydrogen atom, which was added back into the structure before the geometry was optimized using DFT. As a result, all three water molecules have a similar hydrogen-bonding environment (see Fig. S6 in the ESI†). The GIPAW DFT results obtained with the PBE method for ^17^O NMR parameters are listed in Table 2; complete GIPAW DFT results from all four methods are given in the ESI.† It can be seen from Table 2 that all six crystallographically independent d-glucose molecules have similar ^17^O NMR parameters (*vide infra*). Thus, within the spectral resolution limit of the 1D ^17^O MAS spectra, we can assume just one ^17^O NMR signal for each oxygen position. For this reason, Fig. 3 shows comparison between experimental ^17^O CS tensor parameters and “averaged” GIPAW DFT results (averaged over the six crystallographically independent glucose molecules in the asymmetric unit). Because the ^17^O chemical shift anisotropies are rather small in glucose, the agreement seen in Fig. 3 is clearly satisfactory. Since the ^17^O QC tensor parameters do not show much variation, we will not examine them further, except to note that the GIPAW DFT calculations are consistent with the experimental results.

**Table 2:** GIPAW DFT results on ^17^O NMR parameters computed with the PBE method for the six crystallographically distinct α-d-glucose molecules in the asymmetric unit of d-glucose/NaCl/H_2_O (2/1/1) cocrystal

Mol	Atom^a^	Atom^b^	*σ*_iso_/ppm	*σ*_11_/ppm	*σ*_22_/ppm	*σ*_33_/ppm	*C*_Q_/MHz	*η*_Q_
A1	O1	O4	234.4	193.4	226.1	283.6	−9.166	0.84
	O2	O5	271.7	240.6	275.1	299.5	9.965	0.98
	O3	O6	248.6	229.2	241.5	275.2	9.514	0.99
	O4	O7	260.6	229.2	256.0	296.6	9.915	0.96
	O5	O8	199.4	166.8	189.2	242.3	10.95	0.86
	O6	O9	281.0	248.1	269.4	325.3	9.88	0.90
A2	O1	O22	236.5	194.2	228.3	287.1	−9.196	0.89
	O2	O23	272.3	242.1	275.8	298.9	10.06	0.96
	O3	O24	249.0	228.9	242.2	276.1	9.579	0.99
	O4	O25	260.2	228.5	255.5	296.5	9.923	0.96
	O5	O26	198.4	166.2	188.9	240.0	10.91	0.87
	O6	O27	281.3	251.2	268.8	324	9.982	0.90
A3	O1	O34	236.6	195.2	229.3	285.4	−9.168	0.86
	O2	O35	271.8	241.1	274.4	299.8	9.947	0.98
	O3	O36	248.8	229.0	241.7	275.6	9.571	0.98
	O4	O37	260.6	228.7	256.4	296.7	9.922	0.96
	O5	O38	199.5	167.4	188.8	242.3	10.96	0.86
	O6	O39	280.9	248.5	269.4	324.9	9.834	0.91
B1	O1	O10	238.4	196.0	231.3	288.0	−9.162	0.93
	O2	O11	269.8	243.5	272.2	293.8	9.964	0.97
	O3	O12	241.8	209.2	246.9	269.4	10.11	0.89
	O4	O13	253.2	219.5	245.2	294.9	10.22	0.92
	O5	O14	201.5	171.5	190.2	242.8	10.89	0.89
	O6	O15	265.3	218.9	253.4	323.6	9.806	0.92
B2	O1	O16	237.0	196.7	229.9	284.5	−9.109	0.89
	O2	O17	269.3	241.8	271.7	294.5	9.870	0.99
	O3	O18	241.0	209.2	245.1	268.6	10.01	0.90
	O4	O19	253.6	219.9	246.3	294.7	10.21	0.91
	O5	O20	202.7	171.7	190.8	245.4	10.92	0.89
	O6	O21	264.2	215.3	253.9	323.3	9.673	0.92
B3	O1	O28	236.3	195.0	228.0	286.0	−9.153	0.91
	O2	O29	269.3	242.8	272.1	292.9	9.993	0.97
	O3	O30	241.4	208.9	245.1	270.2	10.02	0.90
	O4	O31	252.9	219.6	244.2	294.9	10.20	0.92
	O5	O32	201.1	170.4	190.6	242.4	10.88	0.88
	O6	O33	265.7	220.0	252.5	324.7	9.891	0.91

a Atomic numbering according to Scheme 1.

b Atomic numbering in the original crystal structure (CCDC 1281434.cif).^29^

**Fig. 4:**
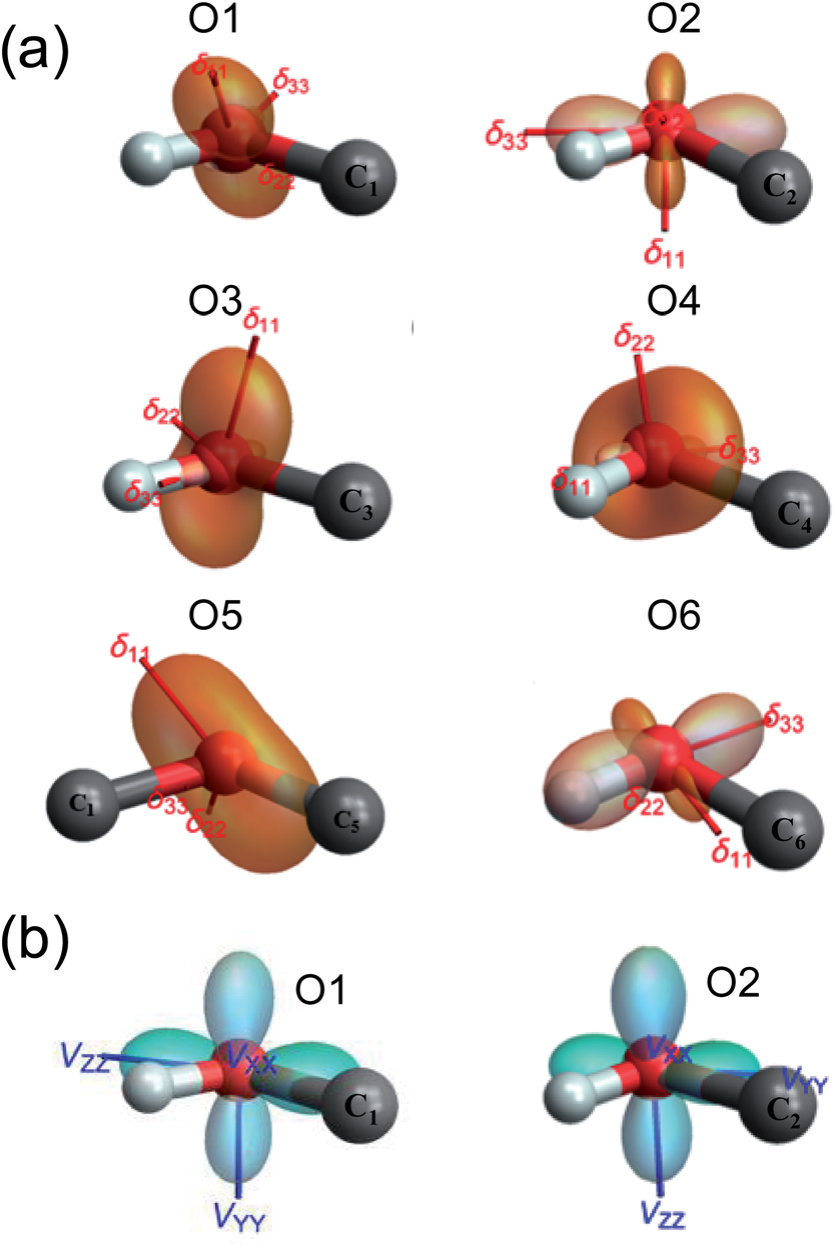
The ovaloid representation of computed ^17^O CS (a) and QC (b) tensor orientations in the molecular frame of α-d-glucose. In (b), the ^17^O QC tensor orientations for O3, O4, O5, and O6 are the same as that shown for O2. See text for discussion.

GIPAW DFT computations also yielded further information about the ^17^O NMR tensor orientations in the molecular frame. In Fig. 4, we used TensorView^65^ to display the ovaloid representation of the ^17^O CS and QC tensors for the six oxygen sites in α-d-glucose. Two general types of orientation were found for the ^17^O CS tensors, as seen in Fig. 4(a). For O1, O2, and O3, the direction of *δ*_11_ appears to be almost perpendicular to the H–O–C plane. For O4, O5, and O6, however, it is the *δ*_22_ component that is perpendicular the H–O–C or C–O–C plane. In all cases, *δ*_33_ lies approximately parallel to the H–O or C–O bonds. Because the ^17^O chemical shift anisotropies in α-d-glucose are generally small, it is difficult to detect any other general trends. Unlike the ^17^O CS tensors, the ^17^O QC tensors for the O–H and C–O–C groups in α-d-glucose were found to be invariant. Although Fig. 4(b) displays two types of QC tensor orientations in the molecular frame, the two seemingly different orientations are essentially the same. This is because the largest QC tensor or EFG tensor component (V_*zz*_) is defined according to its absolute value so that |V_*zz*_| ≥ |V_*yy*_| ≥ |V_*xx*_|. Because all the C–O–H and C–O–C groups in α-d-glucose exhibit *η*_Q_ ≈ 1, the two high tensor components in each case would have very similar magnitudes but opposite signs. The one with the negative sign lies in-plane being perpendicular to the bisector of the C–O–H or C–O–C angle, whereas the one with the positive sign is perpendicular to the C–O–H or C–O–C plane. The smallest QC or EFG tensor component bisects the C–O–H or C–O–C angle; but because this component is always very small for *η*_Q_ ≈ 1, it is hardly seen in the ovaloid representation shown in Fig. 4(b). If the tensor component with the negative sign is of slightly greater magnitude, its direction is defined as V_*zz*_, so *C*_Q_(^17^O) < 0. The ^17^O QC tensor for O1 was found to belong to this case. On the other hand, if the component with the positive sign is larger, *C*_Q_(^17^O) > 0. The ^17^O QC tensors for the O2, O3, O4, O5 and O6 atoms in α-d-glucose were found to belong to this case. However, if the individual tensor components in the molecular frame are directly compared, the two ^17^O QC tensor orientations shown in Fig. 4(b) are rather similar. The link between the tensor orientation and the sign of *C*_Q_(^17^O) was recently explained with the concept of valence p-orbital population anisotropy (VPPA).^66^ Since the ^17^O QC tensor is invariant with respect to the molecular frame for the C–O–H and C–O–C groups, it is worth pointing out that one can use the ^17^O QC tensor as an internal reference to link the ^17^O CS tensor to the molecular frame once the relative orientation between the QC and CS tensors were experimentally determined.

### Solid-state ^17^O NMR at high magnetic fields

**Fig. 5:**
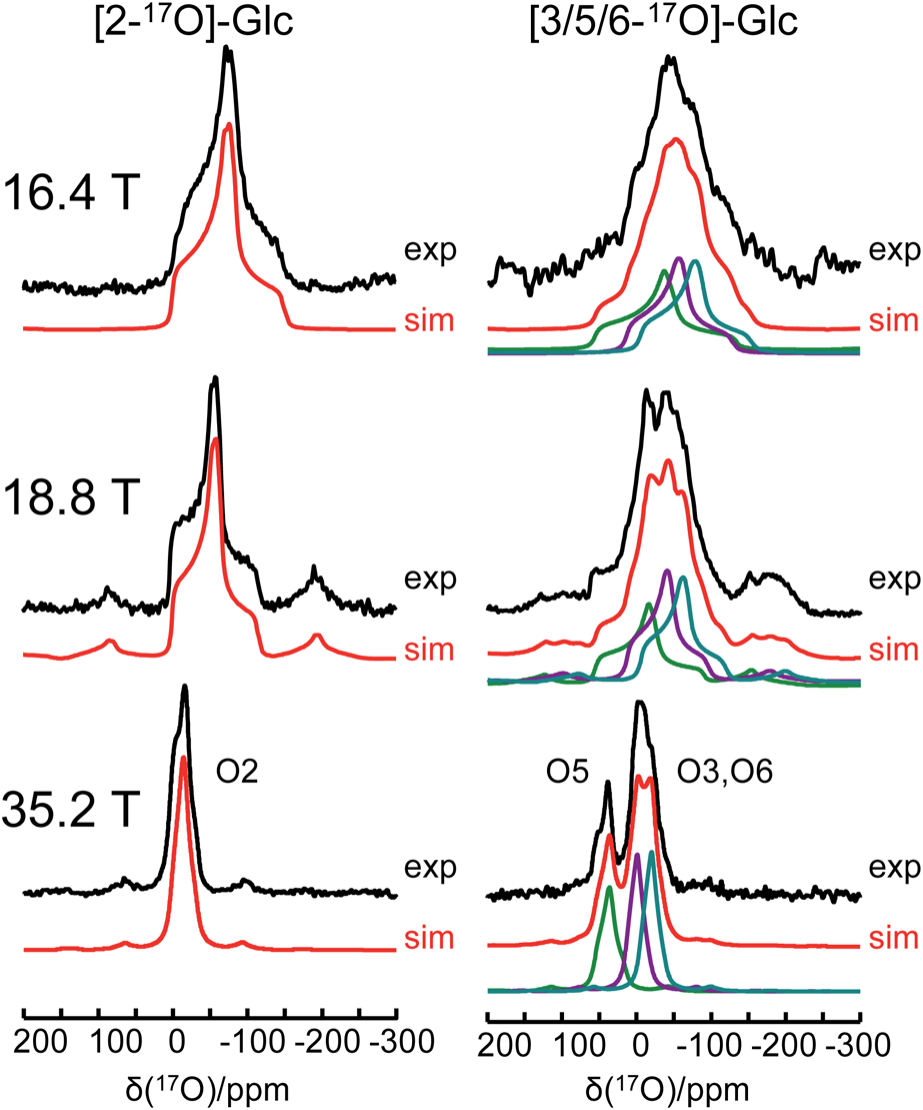
Experimental (black traces) and simulated (red traces) ^17^O MAS NMR spectra of [2-^17^O]-α-d-glucose and [3/5/6-^17^O]-α-d-glucose at three magnetic fields.

One of the major challenges in solid-state ^17^O NMR studies of carbohydrate compounds is that all oxygen-containing functional groups are either hydroxyl or ether groups. As a result, they exhibit very similar ^17^O NMR parameters. For example, the ^17^O isotropic chemical shifts for the six oxygen sites in α-d-glucose, given in Table 1, are within a narrow range of 60 ppm. If multiple oxygen sites are simultaneously ^17^O-labeled, it could be very difficult to resolve their ^17^O NMR signals because each signal would be significantly broadened by the second-order quadrupole interaction. Since the second-order quadrupole interaction is inversely proportional to the applied magnetic field, it is often advantageous to perform solid-state ^17^O NMR experiments at the highest possible magnetic field. To test the limit of this brute-force approach, we obtained ^17^O MAS NMR spectra for [2-^17^O]-α-d-glucose and [3/5/6-^17^O]-α-d-glucose at three magnetic fields, 16.4, 18.8, and 35.2 T. As seen from Fig. 5, the ^17^O NMR signals are progressively sharpened as the applied magnetic field strength increases. For example, at 16.4 T, the line width at the base of the signal for [2-^17^O]-α-d-glucose is 200 ppm. This line width is reduced to approximate 50 ppm at 35.2 T. Remarkably, at 35.2 T, the three oxygen sites in [3/5/6-^17^O]-α-d-glucose become partially resolved. It is also interesting to compare the ^17^O MAS NMR spectrum for [3/5/6-^17^O]-α-d-glucose shown in Fig. 5 with the ^17^O QCT spectrum reported by Gan *et al.*^28^ for the same compound in the slow motion regime. In isotropic liquids, the ^17^O NMR signals are broadened by the intrinsic transverse (*T*_2_) spin relaxation. In the slow motion regime (ω_0_*τ*_c_ ≫ 1), the quadrupole relaxation becomes multi-exponential with only the slow quadrupole relaxation component corresponding to the central transition being detected in the ^17^O QCT spectra. At 35.2 T, the ^17^O QCT signals are significantly narrower than the MAS signals. One additional benefit for studying carbohydrates at ultrahigh magnetic fields is that the oxygen sites in carbohydrates exhibit rather small ^17^O chemical shift anisotropies (CSAs). As seen from Fig. 5, no significant spinning sidebands are observed at 35.2 T. In contrast, ^17^O MAS NMR signals obtained at 35.2 T from protein backbone oxygen atoms display many spinning sidebands.^14^ Because the cross-relaxation between CSA and second-order quadrupole interactions becomes more important at high magnetic fields, ^17^O QCT spectra will display higher resolution for carbohydrates (with small CSAs) than for proteins (with large CSAs).^67^

### Combination of paramagnetic doping and CPMAS CryoProbe technology

**Fig. 6:**
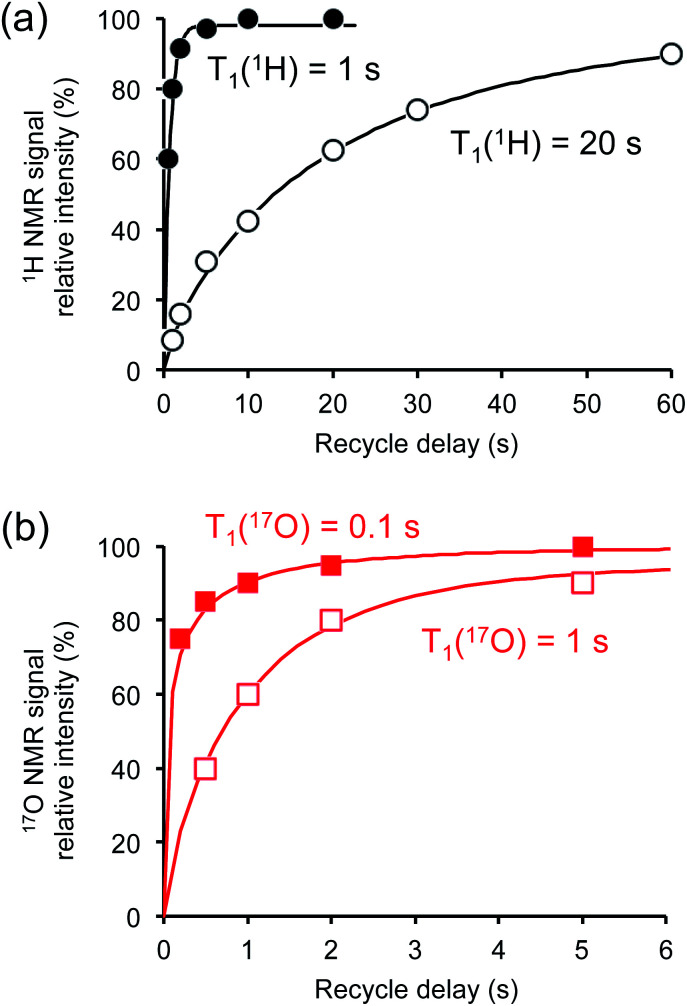
Effects of paramagnetic Cu(ii)–EDTA doping on (a) ^1^H and (b) ^17^O spin–lattice relaxation times. All measurements were carried out at 14.1 T for the [2-^17^O]-d-glucose/NaCl/H_2_O cocrystal with (closed symbols) and without (open symbols) added Cu–EDTA.

**Fig. 7:**
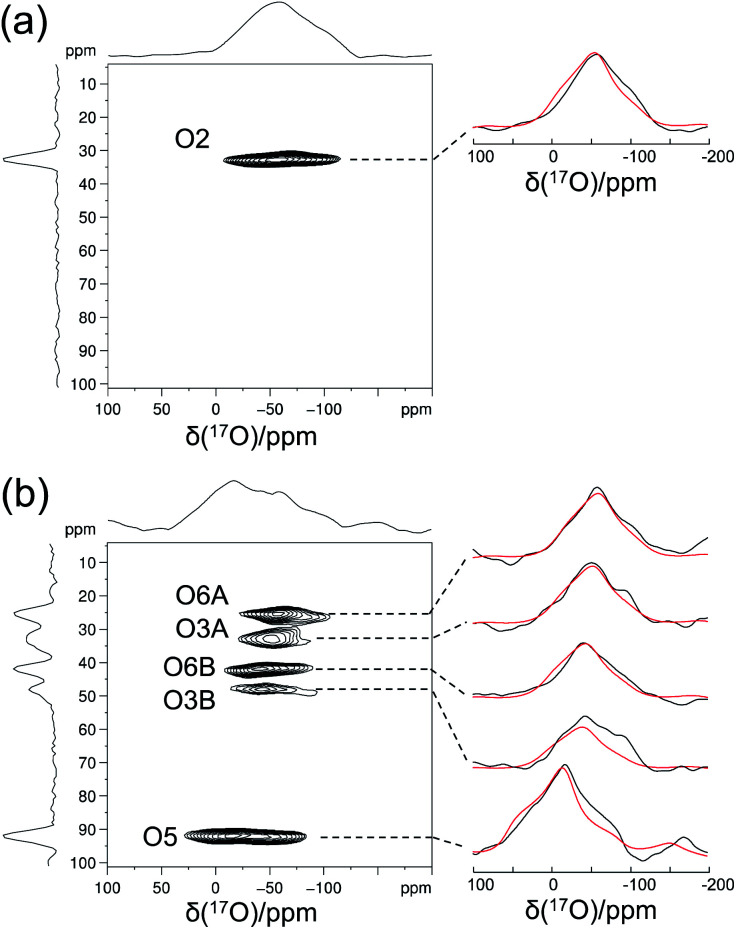
2D ^17^O 3QMAS spectra of (a) [2-^17^O]-α-d-glucose and (b) [3/5/6-^17^O]-α-d-glucose obtained at 18.8 T with a Bruker 3.2 mm CPMAS CryoProbe. The sample spinning frequency was 15 kHz. Each sample contains 10% (w/w) Cu–EDTA. Experimental (black traces) and simulated (red traces) F2-slice spectra are displayed on the side. Data acquisition parameters are: (a), recycle delay 1 s, 48 *t*_1_ increments (with apodization weighted sampling, *n*_0_ = 1536 transients), total experimental time 10 h; (b) recycle delay 1 s, 32 *t*_1_ increments (with apodization weighted sampling, *n*_0_ = 31 680 transients), total experimental time 6 days.

Crystalline d-glucose is known for its exceedingly long *T*_1_(^1^H) values. It was observed that *T*_1_(^17^O) values are also long for d-glucose compounds, hindering rapid repetition of ^17^O data acquisition. One common approach that has been widely employed in cross polarization (CP)-based solid-state ^13^C NMR studies is to add paramagnetic Cu(ii) dopants to shorten *T*_1_(^1^H).^68–70^ In this work, we hypothesized that the same paramagnetic doping approach might be useful for ^17^O NMR studies as well. To this end, we prepared two d-glucose/NaCl/H_2_O cocrystal samples, [2-^17^O]-α-d-glucose and [3/5/6-^17^O]-α-d-glucose, each containing 10% (w/w) Na_2_[Cu(EDTA)_2_]. Fig. 6 shows the effects of paramagnetic doping on the ^1^H and ^17^O NMR signals of [2-^17^O]-α-d-glucose. We found that paramagnetic doping at the 10% (w/w) level shortens the *T*_1_(^1^H) and *T*_1_(^17^O) values in [2-^17^O]-α-d-glucose by about 20 and 10 times, respectively. The [3/5/6-^17^O]-α-d-glucose cocrystal sample containing Cu(ii)-EDTA exhibited similar results. This shortening of *T*_1_(^17^O) allowed more rapid data acquisition, effectively enhancing the sensitivity by approximately a factor of 2. However, the reduction of *T*_1_(^17^O) alone is still insufficient when performing more demanding experiments such as 2D ^17^O multiple-quantum MAS^71,72^ for the α-d-glucose samples prepared in this work. To further increase sensitivity, we combined the paramagnetic doping with new CPMAS CryoProbe technology. It has recently been shown that a CPMAS CryoProbe provides a 3–4 times higher sensitivity for detecting ^13^C and ^15^N nuclei compared to a conventional MAS probe.^32^ After the submission of this work, we learned that Michaelis and co-workers^73^ also obtained some preliminary solid-state ^17^O NMR data using the CPMAS CryoProbe. For acquiring ^17^O MAS spectra for the α-d-glucose compounds, we found that the combination of paramagnetic doping and CPMAS CryoProbe yielded a sensitivity gain by a factor of 6–8. Fig. 7 shows the 2D ^17^O 3QMAS spectra obtained for [2-^17^O]-α-d-glucose and [3/5/6-^17^O]-α-d-glucose samples doped with Cu–EDTA. This is the first time that 2D ^17^O 3QMAS spectra are reported for carbohydrate compounds. It should be emphasized that the level of ^17^O enrichment in the [3/5/6-^17^O]-α-d-glucose sample was only about 10%. Thus, the observed sensitivity shown in Fig. 7 is quite remarkable. Interestingly, whereas each of the O3 and O6 signals appears to split into two signals, no signal splitting was observed for the O2 and O5 signals (*vide infra*). We were able to fit the F2-slice spectra and obtained the following ^17^O NMR parameters: O2, *δ*_iso_ = 2 ppm, *C*_Q_ = 9.1 MHz, *η*_Q_ = 1.0; O3A, *δ*_iso_ = 6 ppm, *C*_Q_ = 8.8 MHz, *η*_Q_ = 0.9; O3B, *δ*_iso_ = 12 ppm, *C*_Q_ = 8.8 MHz, *η*_Q_ = 0.9; O6A, *δ*_iso_ = −6 ppm, *C*_Q_ = 8.8 MHz, *η*_Q_ = 0.9; O6B, *δ*_iso_ = 8 ppm, *C*_Q_ = 8.8 MHz, *η*_Q_ = 0.9; O5, *δ*_iso_ = 56 ppm, *C*_Q_ = 9.9 MHz, *η*_Q_ = 1.0. These values are also confirmed by the signal positions in the isotropic dimension of the ^17^O 3QMAS spectrum; see ESI.† As expected, the ^17^O NMR parameters for O2 and O5 are identical to those extracted from 1D MAS spectra as listed in Table 1. For O3 and O6, in contrast, the unprecedented spectral resolution offered by 2D ^17^O 3QMAS spectra revealed finer spectral details. We will further discuss these new details in the next section.

### Further ^17^O and ^13^C NMR signal assignments

**Fig. 8:**
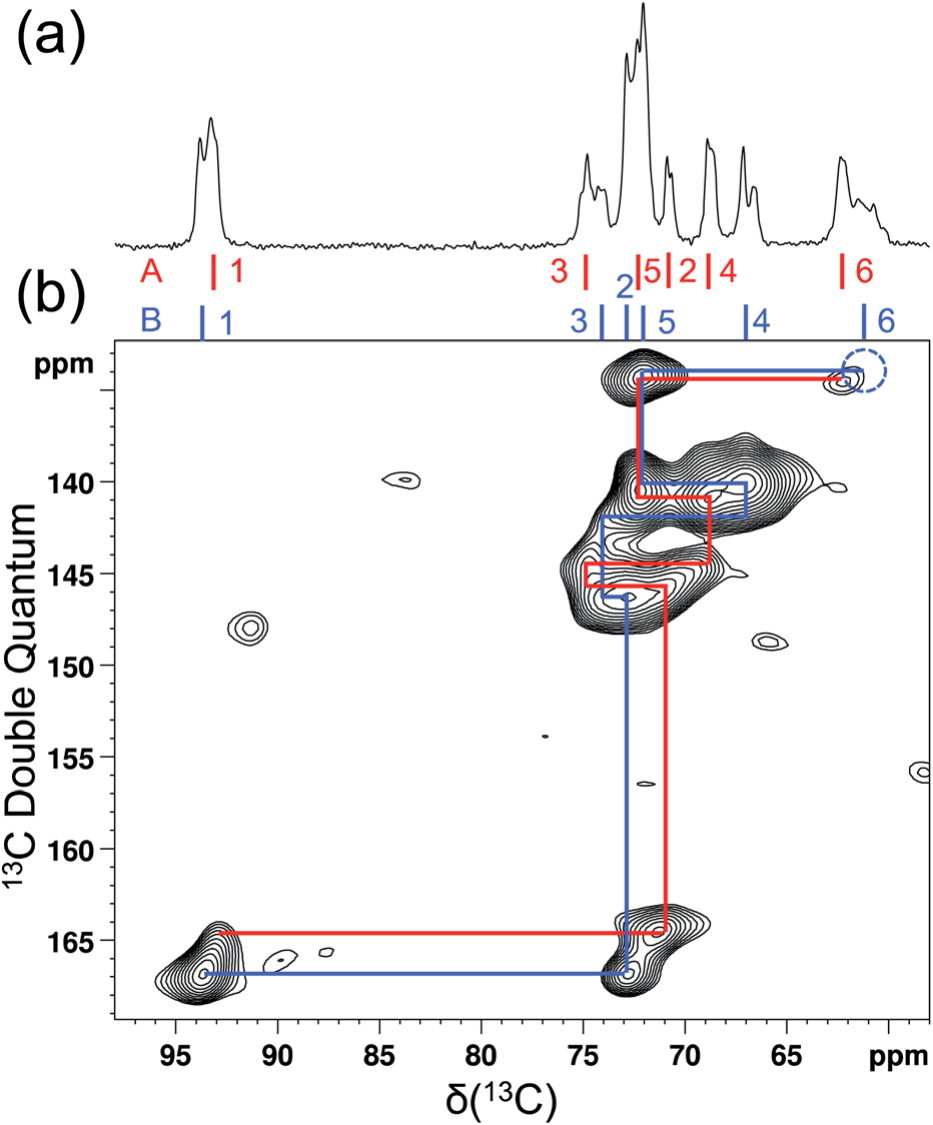
Natural abundance ^13^C (a) 1D CPMAS and (b) 2D refocused INADEQUATE NMR spectra of [2-^17^O]-α-d-glucose doped with 10% (w/w) Cu–EDTA. The dotted blue circle indicates the absence of the C6B signal due to its relatively short *T*_2_ (3.7 ms). Both spectra were obtained at 18.8 T with a Bruker 3.2 mm CPMAS CryoProbe. The sample spinning frequency was 15 kHz. Data acquisition parameters are: (a), 1D CP/MAS, contact time 3 ms, recycle time 3.7 s, 16 transients; (b), 2D refocused INADEQUATE, recycle delay 2 s, 1536 transients per *t*_1_, 34 *t*_1_ increments, J-evolution period of 3.99 ms, total experimental time 30 h.

**Fig. 9:**
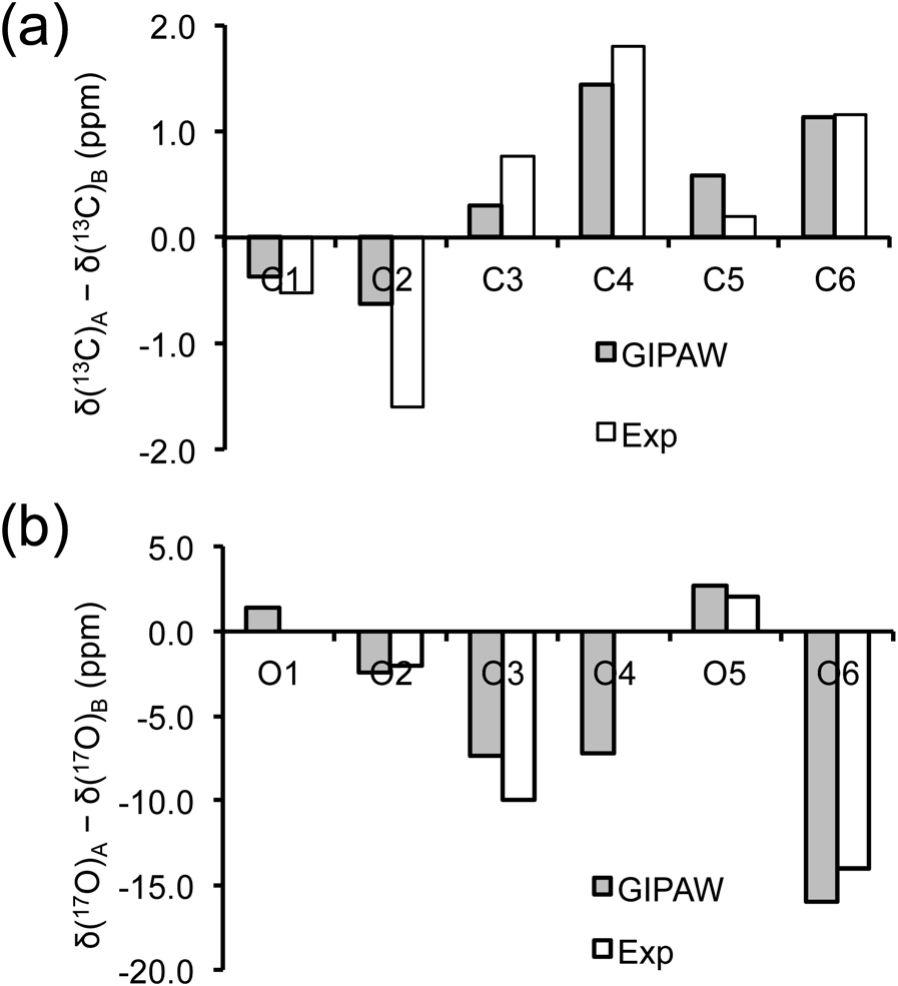
Comparison between observed and GIPAW DFT calculated (a) ^13^C and (b) ^17^O chemical shift differences between Molecules A and B in α-d-glucose. In (b), because the line width observed in the 3Q isotropic dimension for the O2 and O5 3QMAS signals was about 5 ppm, the upper limit of any potential signal splittings for O2 and O5 was estimated to be 2 ppm.

As mentioned earlier, there are six crystallographically independent glucose molecules in the asymmetric unit of d-glucose/NaCl/H_2_O cocrystal. Thus, in principle, there should be six ^17^O NMR signals for each oxygen atom in this compound. However, the six crystallographically independent glucose molecules form three Na^+^-chelated glucose “dimers” with very similar structures. For this reason, the two different signals observed for each of the O3 and O6 groups in the 2D ^17^O 3QMAS spectrum shown in Fig. 7 can be attributed to the two types of α-d-glucose molecules, A and B, within each Na^+^-chelated glucose “dimer”. This also implies that the difference among the three “dimers” cannot be detected with the current spectral resolution. The tentative signal assignments shown in Fig. 7 were based on the GIPAW DFT calculations listed in Table 2. To further confirm this hypothesis, we decided to fully assign the solid-state ^13^C NMR signals for the same α-d-glucose sample. To this end, we obtained a 2D refocused INADEQUATE NMR spectrum at the ^13^C natural-abundance isotope level for the same compound using the CPMAS CryoProbe. As seen from Fig. 8, a similar signal “doubling” was indeed observed for each carbon atom. Fig. 8 also shows the ^13^C NMR signal assignment for Molecules A and B, based on GIPAW DFT results for ^13^C chemical shifts (provided in the ESI). In fact, in the 1D ^13^C CPMAS spectrum shown in Fig. 8, there are also hints that smaller resonance splittings beyond the signal “doubling” are also present for C1, C2A, C3, C4, and C6B. Unfortunately, within the currently achievable spectral resolution, it is not possible to resolve all six ^13^C NMR signals for each site. So, for now we focus on the chemical shift differences between Molecules A and B within the glucose “dimer”. Clearly, for different carbon sites, the ^13^C chemical shift differences between Molecules A and B show different patterns. We will further examine these patterns for all the carbon and oxygen atoms in α-d-glucose. Fig. 9 shows a comparison between experimental and GIPAW DFT results with the PBE method for both ^13^C and ^17^O chemical shifts; complete GIPAW DFT results from all four methods are provided in the ESI.† The observed general agreement between experiment and computation suggests that the reported signal assignment is quite reasonable. Now we can understand why no “doubling” or “splitting” was observed for the O2 and O5 signals in the ^17^O 3QMAS spectra shown in Fig. 7. As seen from Fig. 9, the GIPAW DFT calculations predict that the ^17^O chemical shift difference between Molecules A and B is indeed rather small for O2 and O5 (<3 ppm). It is also evident from Fig. 9 that the ^17^O chemical shift is a much more sensitive probe than the ^13^C chemical shift to any structural variation. In practice, however, the generally lower spectral resolution encountered in ^17^O NMR often makes it difficult to fully utilize such sensitivity. On the other hand, it is also not difficult to imagine that, in some cases, the superior sensitivity of ^17^O NMR to molecular structure and chemical bonding can produce information that is unobtainable by ^13^C NMR. Ideally, one should utilize all available magnetically-active nuclei in a molecular system as a general approach of “NMR crystallography”.^74^

**Fig. 10:**
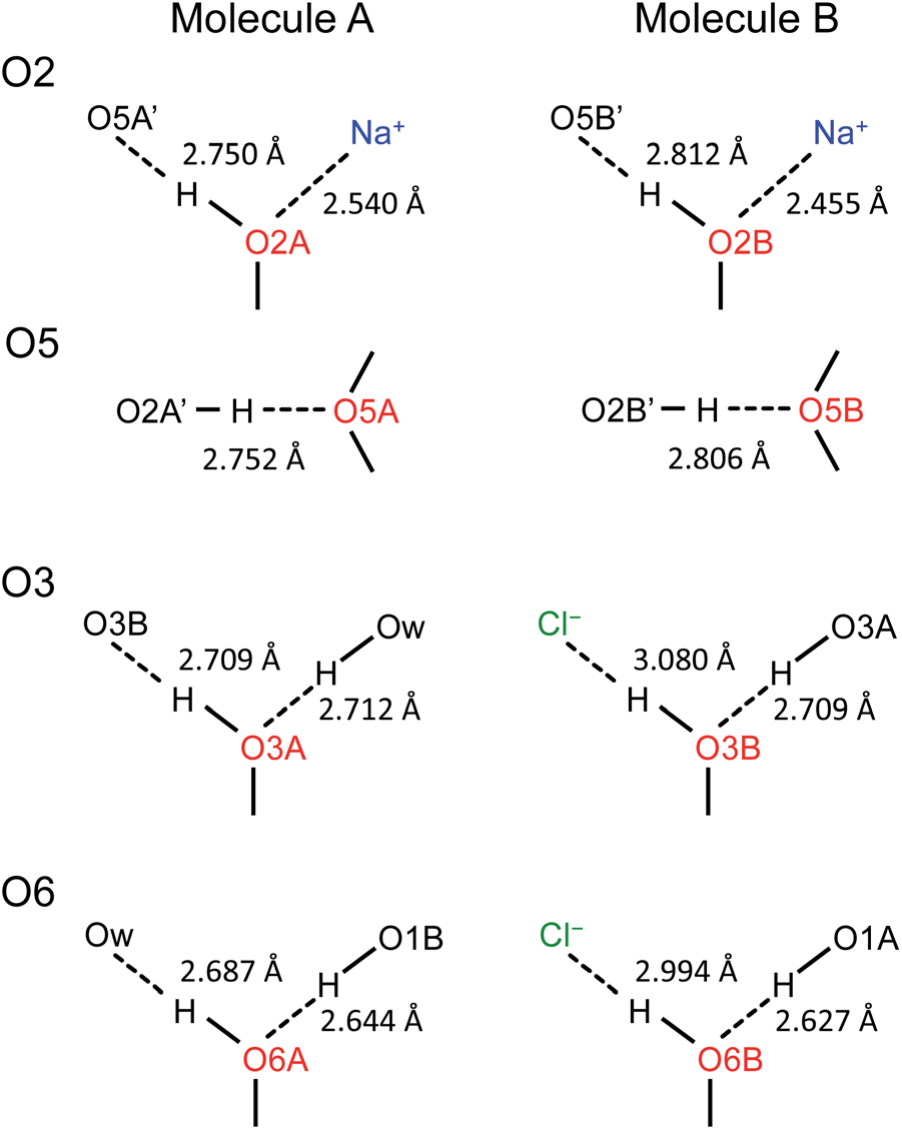
Comparison of hydrogen-bonding environments around the O2, O5, O3, and O6 atoms between Molecules A and B in the d-glucose/NaCl/H_2_O cocrystal. Distances between the two heavy atoms in each hydrogen bond are listed.

Now, what are the reasons for the ^17^O chemical shift differences between Molecules A and B to show the patterns displayed in Fig. 9? Why do the O2 and O5 atoms between Molecules A and B exhibit very similar ^17^O chemical shifts (within 2 ppm), but the O3 and O6 atoms have so different values (by more than 10 ppm)? To link the structural features to these spectral characteristics, we will need to further examine the crystal structure of the d-glucose/NaCl/H_2_O cocrystal. Fig. 10 summarizes the hydrogen-bonding and ion-coordination environments around the O2, O5, O3 and O6 atoms in Molecules A and B. Clearly, the O2 and O5 atoms have essentially the same hydrogen-bonding and ion-coordination environments between Molecules A and B. In both Molecules A and B, the O2 atom forms a hydrogen bond of the O–H⋯O type and is also coordinated to a Na^+^ ion. In sharp contrast, the O3 and O6 atoms display quite different hydrogen-bonding environments between Molecules A and B. As seen from Fig. 10, the key structural difference is the replacement of a neutral O–H⋯O hydrogen bond in Molecule A by a stronger ionic O–H⋯Cl^−^ hydrogen bond in Molecule B. Thus, both O3 and O6 experience stronger hydrogen-bonding environments in Molecule B than in Molecule A. For example, the hydrogen bond enthalpies for the H–O–H⋯OH_2_ and H–O–H⋯Cl^−^ dimers are 3.6 and 13.5 kcal mol^−1^, respectively.^75^ While hydrogen-bonding effects on ^17^O NMR parameters are well known for carbonyl compounds,^76–86^ data on hydroxyl and ether functional groups are scarce in the literature. The best-known case is that the ^17^O NMR signal from gaseous H_2_O was found at *δ*(^17^O) = −36.1 ppm with respect to that from liquid H_2_O, *δ*(^17^O) = 0 ppm.^87–89^ This means that hydrogen-bonding interactions would cause deshielding on the ^17^O nucleus of the O–H group (thus increase in the *δ*(^17^O) value). This general trend was first firmly established by Reuben^90^ in the study of solvent effects on ^17^O chemical shifts. The same trend was also observed for the hydronium ion H_3_O^+^ in the solid state.^91^ In the present case of the d-glucose/NaCl/H_2_O cocrystal, because the O3 and O6 atoms are involved in stronger hydrogen-bonding interactions in B than in A, the values of *δ*(^17^O)_A_ − *δ*(^17^O)_B_ are negative for both O3 and O6, as seen from Fig. 10. Thus, the unprecedented resolution in the 2D ^17^O 3QMAS spectra allowed us to detect a subtle structural difference between the two crystallographically distinct molecules. More specifically, we found that replacement of a neutral O–H⋯O hydrogen bond by a stronger ionic O–H⋯Cl^−^ hydrogen bond causes an increase in *δ*(^17^O) by *ca.* 10–14 ppm. Once again, this finding illustrates the remarkable sensitivity of ^17^O NMR parameters to hydrogen bonding interactions. Interestingly, the GIPAW DFT calculations showed that the protons attached to O3B and O6B are also significantly deshielded by 2–3 ppm, due to the stronger hydrogen bonding, than the corresponding protons attached to O3A and O6A.

## Conclusions

We have carried out a comprehensive solid-state ^17^O NMR study for α-d-glucose. In this work, a total of six site-specifically ^17^O-labeled α-d-glucose compounds were synthesized. The ^17^O CS and QC tensors were determined for each of the six oxygen sites in α-d-glucose from an analysis of solid-state ^17^O NMR spectra obtained at multiple magnetic fields. This is the first case where all oxygen-containing functional groups in a carbohydrate molecule are site-specifically ^17^O-labeled and have their ^17^O NMR tensors fully characterized. We found that paramagnetic Cu(ii) doping can significantly shorten the *T*_1_(^17^O) values for solid α-d-glucose samples, making it possible to rapidly collect ^17^O NMR data. By combining the paramagnetic doping effect with the new CPMAS CryoProbe technology and apodization weighted sampling at high magnetic fields, we have achieved a significant sensitivity boost that allowed us to obtain the first set of ^17^O 3QMAS spectra ever reported for carbohydrate compounds. The unprecedented resolution offered by 2D ^17^O 3QMAS spectra permitted the detection of a subtle structural difference for a single hydrogen bond between two types of crystallographically distinct d-glucose molecules. With the aid of GIPAW DFT calculations, all observed ^17^O and ^13^C NMR signals were assigned to the two groups of crystallographically distinct α-d-glucose molecules. This combined ^17^O and ^13^C solid-state NMR approach adds a new dimension to the field of “NMR crystallography”. Successful synthesis of site-specifically ^17^O-labeled d-glucose also paves the way for researchers to consider ^17^O NMR as a new spectroscopic tool in glucose-related research, which can range from glucose binding proteins to glucose metabolism of live cells. In a broader context, this work demonstrates that continuing advancement of solid-state ^17^O NMR spectroscopy has begun to open the door for studying many biological molecules that are usually considered too difficult for ^17^O NMR spectroscopy. It is about time to add ^17^O to the NMR toolbox for probing organic and biological molecules.

## Author contributions

J. Shen synthesized the ^17^O-labeled compounds and recorded solid-state ^17^O NMR data at 14.1 T. VT obtained solid-state ^17^O NMR spectra at 21.1 T. J. Struppe, AH, and MM acquired the solid-state ^17^O and ^13^C NMR data at 18.8 T with the CPMAS CryoProbe. IH and ZG conducted solid-state ^17^O NMR experiments at 35.2 T. AB performed the GIPAW DFT calculations. GW designed the project, supervised the work, obtained solid-state ^17^O NMR data at 16.4 T and ^13^C CPMAS data at 14.1 T, and interpreted the results. All authors contributed to the writing of the manuscript.

## Conflicts of interest

There are no conflicts to declare.

## Supplementary Material

SC-013-D1SC06060K-s001
