# Supplementary material for: Solid-state 17O NMR study of α-d-glucose: exploring new frontiers in isotopic labeling, sensitivity enhancement, and NMR crystallography
Source: Chem Sci. 2022 Jan 3;13(9):2591–603. doi: 10.1039/d1sc06060k (PMC8890099; doi:10.1039/d1sc06060k)
Supplement: SC-013-D1SC06060K-s001 [file SC-013-D1SC06060K-s001.pdf]

Electronic Supplementary Material (ESI) for Chemical Science.
This journal is © The Royal Society of Chemistry 2022

Electronic Supplementary Information

Solid-State ^{17}O NMR Study of α-D-Glucose: Exploring New Frontiers in Isotopic Labeling, Sensitivity Enhancement, and NMR Crystallography

Jiahui Shen,[1] Victor Terskikh,[2] Jochem Struppe,[3] Alia Hassan,[4] Martine Monette,[5] Ivan Hung,[6] Zhehong Gan,[6] Andreas Brinkmann,[2] and Gang Wu[1]*

[1]Department of Chemistry, Queen's University, 90 Bader Lane, Kingston, Ontario K7L 3N6, Canada; [2]Metrology, National Research Council Canada, Ottawa, Ontario K1A 0R6, Canada; [3]Bruker Biospin Corporation, 15 Fortune Drive, Billerica, MA 01821, United States; [4]Bruker Switzerland AG, Fällanden, Switzerland; [5]Bruker Biospin Ltd., 2800 High Point Drive, Suite 206, Milton, Ontario L9T 6P4, Canada; [6]National High Magnetic Field Laboratory, 1800 East Paul Dirac Drive, Tallahassee, Florida 32310, United States.

*Corresponding author: wugang@queensu.ca

Table of Contents

1. Detailed synthetic procedures and compound characterization ….……………….. S3

1.1 Synthesis of [1-^{17}O]-D-glucose………………………………………….… S3

1.2 Synthesis of [2-^{17}O]-D-glucose …………………………………………..…S3

1.3 Synthesis of [3-^{17}O]-D-glucose …………………………………………..…S4

1.3.1 Triflate displacement reaction ……………………………….…S4

1.3.2 Mitsunobu reaction ……………………………………………..…S6

1.4 Synthesis of [4-^{17}O]-D-glucose …………………………………………..…S8

1.5 Synthesis of [5-^{17}O]-D-glucose …………………………………………..…S9

1.6 Synthesis of [6-^{17}O]-D-glucose ……………………………………………S12

1.7 Summary of ^{17}O NMR data for reaction intermediates and final products...S14

2. Solid-state ^{17}O NMR ………………………..…………………………………S17

2.1 Data acquisition parameters for 1D experiments …………………………S18

2.2 Signal positions in 2D 3QMAS spectra …………………………………..S18

3. Solid-state ^{13}C NMR characterization of solid samples ………..……………………..S19

4. GIPAW DFT results

4.1 Hydrogen bonding interactions around water molecules …………………S20

4.2 Complete GIPAW results on ^{17}O CS and QC tensors ……………….……S21

4.2 Complete GIPAW results on ^{13}C isotropic magnetic shielding constants…S25

Appendix:

Supplementary solution ^{1}H, ^{13}C, ^{17}O NMR spectra……………………………….….. S29

1. Detailed synthetic procedures and compound characterization

1.1 Synthesis of [1-^{17}O]-D-glucose

Natural abundance D-glucose (150 mg, 0.83 mmol) was dissolved in 0.2 mL $H_2{}^{17}O$ (40% ^{17}O, purchased from Cambridge Isotope Laboratories) in a 5-mm NMR tube. The NMR tube was left at 40 °C for 2 days. At this point, solution ^{17}O NMR showed that the ^{17}O exchange process was complete. The solvent was then evaporated to give a clear syrup of [1-^{17}O]-D-glucose (125 mg, 0.65 mmol, yield 83%). ^{17}O NMR (67.7 MHz, D_2O): δ = 47 (β form), 37 (α form), 0 ppm (D_2O).

1.2 Synthesis of [2-^{17}O]-D-glucose

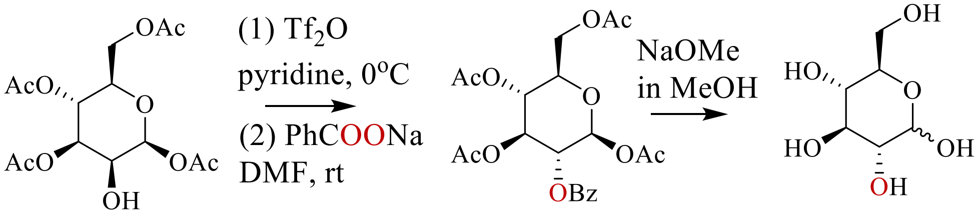

(2I) (2II)

Scheme S1. Synthetic route for preparation of [2-^{17}O]-D-glucose.

1,3,4,6-Tetra-O-acetyl, [2-^{17}O]-benzoyl-β-D-mannopyranose (2I). In a 100 mL flask added with 1,3,4,6-tetra-O-acetyl-β-D-mannopyranose (174 mg, 0.5 mmol) and anhydrous pyridine (20 mL). After fully dissolved, Tf_2O (0.17 mL, 1 mmol) was added into the solution at 0 °C and stirred for an hour. The solution became pale yellow. DCM (20 mL) was then added to dilute the solution and the mixture was washed with ice cold 5% HCl and cold water, and dried using Na_2SO_4. The concentrated oil was later dissolved in DMF (20 mL) added with ^{17}O-labeled sodium benzoate (88 mg, 1.2 mmol) and stirred for 12 h. The mixture was then diluted with DCM, washed with water, saturated

$NaHCO_3$, and NaCl, and then concentrated to dryness. The mixture was later re-dissolved in minimum amount of DCM and column chromatography (Hexane: EtOAc = 3: 1) was run to give the product 2I (194 mg, 0.32 mmol, yield: 90%). ^{1}H NMR (400 MHz, $CDCl_3$-*d*): δ = 8.00 (d, J= 7.65 Hz, 2H, aromatic), 7.61 (t, J= 7.65 Hz, 1H, aromatic), 7.48 (d, J=7.65 Hz, 2H, aromatic), 5.91 (H-1, d, $J_{1,2}$ = 7.82 Hz, 1H), 5,46 (H-2, H-5, m, 2H), 5.23 (H-3, t, $J_{3,2}$ = $J_{3,4}$ = 9.01 Hz, 1H), 4.35 (H-6a, dd, $J_{6a,5}$ = 4.42 Hz, $J_{H6a,6b}$ = 12.38 Hz, 1H), 4.16 (H-6b, dd, $J_{H6a,6b}$ =12.38 Hz, $J_{5,6b}$ = 1.81 Hz, 1H), 3.94 (H-4, m, 1H), 2.14 (Me, s, 3H), 2.06 (Me, s, 6H), 1.95 (Me, s, 3H). ^{13}C NMR (100 MHz, $CDCl_3$-*d*): δ = 170.61, 170.01, 169.39, 164.90, 133.69, 129.87, 128. 60, 91.90, 72.93, 72.58, 70.63, 67.85, 61.55, 20.76, 20.71, 20.57, 20.49 ppm. ^{17}O NMR (54 MHz, $CDCl_3$-*d*): δ = 342, 152 ppm.

[2-^{17}O]-D-glucose (2II). All the product 2I was dissolved into methanol (20 mL), NaOMe powder (30 mg, 0.56 mmol) was added and the mixture was stirred for 3 hours and then neutralized using Amberlite IR 120 H^+ and then filtered. The solution was dried to give syrup product 2II (65 mg, 0.36 mol, yield: 84%). ^{17}O NMR (54 MHz, D_2O): δ = 10, 0 ppm (D_2O).

1.3 Synthesis of [3-^{17}O]-D-glucose

1.3.1 Triflate displacement reaction

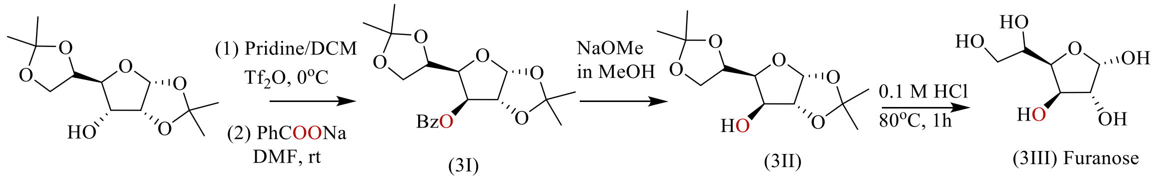

Scheme S2. Synthetic route for preparation of [3-^{17}O]-D-glucose by triflate displacement reaction.

[3-^{17}O]-Benzoyl-1, 2-O-isopropylidene-α-D-glucofuranose (3I). In a 100 mL flask added with 1,2:5,6-Di-O-isopropylidene-α-D-allofuranose (260 mg, 2 mmol) and 20:1 DCM/anhydrous pyridine solvent (50 mL). After fully dissolved, Tf_2O (0.68 mL, 4 mmol) was added into the solution at 0 ^{o}C and stirred for an hour. The solution became pale yellow. DCM (50 mL) was then added to dilute the solution and then the mixture was washed with ice cold 5% HCl, cold water and dried using Na_2SO_4. After that, the mixture was dissolved in DMF (50 mL) and ^{17}O-labeled sodium benzoate (432 mg, 3 mmol) was added, and the mixture was stirred for 12 h. The mixture was then diluted with DCM, washed with water, saturated $NaHCO_3$, and NaCl, and then concentrated to dryness. The mixture was then re-dissolved in minimum amount of DCM and column chromatography (Hexane: EtOAc = 3: 1) was run to give the product 3III (500 mg, 1.37 mmol, yield: 69%). ^{1}H NMR (400MHz, $CDCl_3$-*d*): δ = 8.03 (m, 2H, aromatic), 7.60 (m, 1H, aromatic), 7.47 (m, 2H, aromatic), 5.96 (H-1, d, $J_{1,2}$ = 3.6 Hz, 1H), 5,52 (H-3, d, $J_{3,4}$ = 2.7 Hz, 1H), 4.64 (H-2, d, $J_{2,1}$ = 3.6 Hz, 1H), 4.34 (H-4, H-5, m, 2H), 4.11 (H-6a, H-6b, m, 2H), 1.56 (Me, s, 3H), 1.43 (Me, s, 3H), 1.33 (Me, s, 3H), 1.28 (Me, s, 3H); ^{17}O NMR (54 MHz, $CDCl_3$-*d*): δ = 340 ppm, 151 ppm (Benzoyl group).

[3-^{17}O]-1,2;5,6-Di-O-isopropylidene-α-D-glucofuranose (3II). The product obtained from the previous step was dissolved in methanol (30 mL), to which solid NaOMe (30 mg, 0.56 mmol) was added. The mixture was stirred for 2 h and then neutralized using H^+ resin and filtered, dried to give 3II (340 mg, 1.31 mmol, yield: 96%). ^{1}H NMR (300MHz, $CDCl_3$-*d*): δ= 5.92 (H-1, d, $J_{1,2}$ = 3.56 Hz, 1H), 4.51 (H-2, d, $J_{2,1}$ =3.56 Hz, 1H), 4.30 (H-

3, H5, m, 2H), 4.14 (H-6a, dd, $J_{6a,6b}$ = 8.62 Hz, $J_{6a,5}$ = 6.40 Hz, 1H), 4.08 (H-4, dd, $J_{3,4}$ = 7.63 Hz, $J_{4,5}$ = 2.71 Hz, 1H), 3.99 (H-6b, dd, $J_{6a,6b}$ = 8.62 Hz, $J_{6b,5}$ = 5.42 Hz, 1H), 1.48 (Me, s, 3H), 1.43 (Me, s, 3H), 1.35 (Me, s, 3H), 1.30 (Me, s, 3H); ^{17}O NMR (400 MHz, $CDCl_3$-*d*): δ = 0.58 ppm, -37 (methanol).

[3-^{17}O]-D-glucose (3III). A mixture of [3-^{17}O]-1,2;5,6-Di-O-Isopropylidene-α-D-glucofuranose (340 mg, 1.31 mmol) and 5 mL of 0.1 M HCl was heated at 80 °C for 1 h. The mixture was then neutralized with OH^- resin. The solution was evaporated to dryness. Addition of a minimum amount of absolute ethanol induced crystallization to yield crystalline glucose 3III (112 mg, 0.62 mmol, yield: 47%). ^{17}O NMR (54 MHz, D_2O): δ= 13, 0 ppm (D_2O).

1.3.2 Mitsunobu reaction

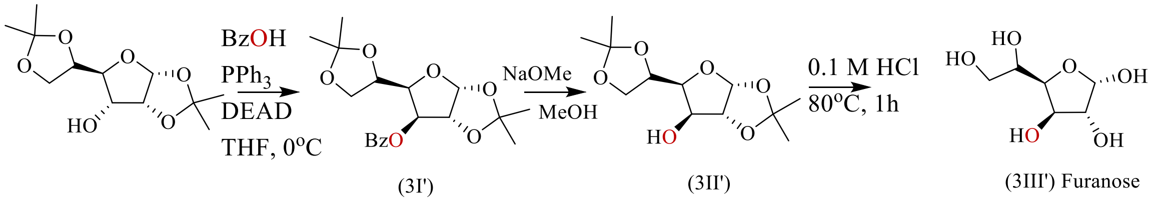

Scheme S3. Synthetic route for preparation of [3-^{17}O]-D-glucose by Mitsunobu reaction.

[3-^{17}O]-Benzoyl-1, 2-O-isopropylidene-α-D-glucofuranose (3I'). In a 100 mL flask added with 1,2:5,6-Di-O-isopropylidene-α-D-allofuranose (260 mg, 2 mmol) and anhydrous THF (50 mL). After fully dissolved, ^{17}O-labeled BzOH (366 mg, 3 mmol), PPh_3 (1048 mg, 4 mmol) was added. Then it was treated with (0.64 mL, 4 mmol) DEAD dropwise in 0 °C with ice-bath. The mixture was stirred for 24 h at room temperature.

TLC plate analysis shows four points (the second one is the product) in eluent (hexane/EtAc 1:1, v/v), the mixture was then re-dissolved in 4 mL DCM and column chromatography (Hexane: EtAc = 20:1 to 8:1) was run to give product 3I' (325 mg, 0.89 mmol, yield: 45%). ^{1}H NMR (400MHz, $CDCl_3$-*d*): δ=8.03 (m, 2H, aromatic), 7.60 (m, 1H, aromatic), 7.47 (m, 2H, aromatic), 5.96 (H-1, d, $J_{1,2}$ = 3.6 Hz, 1H), 5,52 (H-3, d, $J_{3,4}$ = 2.7 Hz, 1H), 4.64 (H-2, d, $J_{2,1}$ = 3.6 Hz, 1H), 4.34 (H-4, H-5, m, 2H), 4.11 (H-6a, H-6b, m, 2H), 1.56 (Me, s, 3H), 1.43 (Me, s, 3H), 1.33 (Me, s, 3H), 1.28 (Me, s, 3H); ^{17}O NMR (54 MHz, $CDCl_3$-*d*): δ = 340 ppm, 151 ppm (Benzoyl group).

[3-^{17}O]-1,2;5,6-Di-O-isopropylidene-α-D-glucofuranose (3II'). Products obtained from the first step were then treated with NaOH in MeOH (~ 0.5 M, ~ 10 mL) and was stirred for 1 h. The mixture was concentrated to 1 mL and then $CHCl_3$ (10 mL) and water (4 mL) was added. The organic phase was separated, and the water phase was further extracted twice (10 mL × 2) with $CHCl_3$. The mixture then became white product 3II' (220 mg, 0.85 mmol, yield: 95%). ^{1}H NMR (300MHz, $CDCl_3$-*d*): δ= 5.92 (H-1, d, $J_{1,2}$ = 3.56 Hz, 1H), 4.51 (H-2, d, $J_{2,1}$ =3.56 Hz, 1H), 4.30 (H-3, H5, m, 2H), 4.14 (H-6a, dd, $J_{6a,6b}$ = 8.62 Hz, $J_{6a,5}$ = 6.40 Hz, 1H), 4.05 (H-4, dd, $J_{3,4}$ = 7.63 Hz, J_{4-5} = 2.71 Hz, 1H), 3.99 (H-6b, dd, $J_{6a,6b}$ = 8.62 Hz, $J_{6b,5}$ = 5.42 Hz, 1H), 1.48 (Me, s, 3H), 1.43 (Me, s, 3H), 1.35 (Me, s, 3H), 1.30 (Me, s, 3H); ^{17}O NMR (400 MHz, $CDCl_3$-*d*): δ=1 ppm, -37 (methanol).

[3-^{17}O]-D-glucose (3III'). A mixture of 3-^{17}O-1,2;5,6-di-O-isopropylidene-α-D-glucofuranose (220 mg, 85%) and 5 mL of 0.1 M HCl was heated at 80 °C for 1 h. The

mixture was then neutralized with OH^- solution. The solution was evaporated to dryness to give colorless syrup 3III' (150 mg, 0.83 mmol, yield: 98%). ^{17}O NMR (54 MHz, D_2O): δ= 13, 0 ppm (D_2O).

1.4 Synthesis of [4-^{17}O]-D-glucose

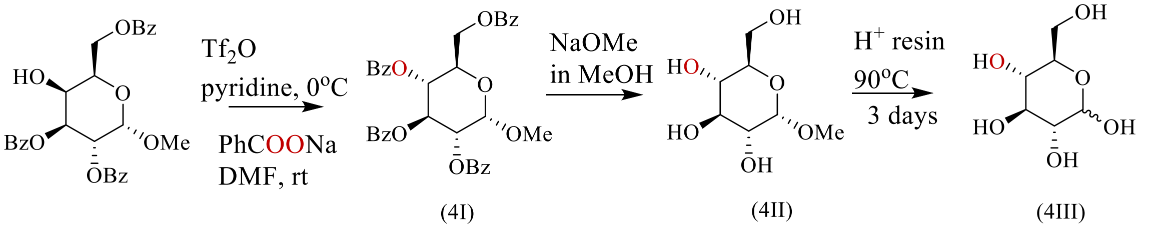

Scheme S4. Synthetic route for preparation of [4-^{17}O]-D-glucose.

[4-^{17}O]-Methyl 2,3,4,6-tetra-O-benzoyl-α-D-glucopyranoside (4I). In a 100 mL flask added with methyl 2,3,6-tri-O-benzoyl-α-D-galactopyranoside (126.5 mg, 0.25 mmol) and 20:1 DCM: pyridine (10 mL). After fully dissolved, trifluoromethanesulfonic anhydride (Tf_2O, 0.08 mL, 0.5 mmol) was added into the solution at 0 °C and stirred for an hour. The solution became pale yellow. DCM (20 mL) was then added to dilute the solution and then the mixture was washed with ice cold 5% HCl and then cold water and dried using Na_2SO_4. After that, the mixture was dissolved in DMF (10 mL) and ^{17}O-labeled sodium benzoate (44 mg, 0.3 mmol) was added, and the mixture was stirred for 12 h. The mixture was then diluted with DCM, washed with water, saturated $NaHCO_3$, and NaCl, and then concentrated to dryness. The mixture was then re-dissolved in minimum amount of DCM and column chromatography (Hexane: EtOAc = 3:1) was run to give product 4I (130 mg, 0.21 mmol, 84%). ^1H NMR (500 MHz, $CDCl_3$-*d*): δ = 7.76

(m, 8H), 7.15 (m, 12H), 5.99 (H-3, dd, $J_{3,2} = J_{3\text{-}4}$ = 9.9 Hz, 1H), 5.49 (H-4, dd, $J_{4,3} = J_{4,5}$ = 9.9 Hz, 1H), 5.08 (H-2, dd, $J_{2,1}$ = 3.34 Hz, $J_{2,3}$ = 9.9 Hz), 5.28 (H-1, d, $J_{1,2}$ = 3.34 Hz, 1H), 4.42 (H-6a, dd, $J_{6a,6b}$ = 11.71 Hz, $J_{6a,5}$ = 2.25 Hz, 1H), 4.30 (H-6b, dd, $J_{6a,6b}$ = 11.71 Hz, $J_{6b,5}$ = 5.41 Hz, 1H), 4.26 (H-5, m, 1H), 3.29 (OMe, s, 3H) . ^{17}O NMR (67 MHz, $CDCl_3$-*d*): δ = 336 ppm, 149 ppm (Benzoyl group).

[4-^{17}O]-Methyl-α-D-glucopyranoside (4II). All the [4-^{17}O]-methyl-2,3,4,6-tetra-O-benzoyl-α-D-glucopyranoside (130 mg, 0.21 mmol) was dissolved in MeOH (20 mL), NaOMe powder (15 mg, 0.27 mmol) was added. The mixture was stirred for 1 h. After that, H^+ resin was added to acidify the mixture and the mixture was concentrated to give 4II (40 mg, 0.21 mmol, yield: 98%). ^{1}H NMR (300 MHz, D_2O): δ = 4.62 (H-1, d, $J_{1,2}$ = 3.47 Hz, 1H), 3.74 (H-6a, dd, $J_{6a,5}$ = 1.44 Hz, $J_{6a,6b}$ = 12.09 Hz, 1H), 3.62 (H-6b, dd, $J_{6b,5}$ = 5.41 Hz, $J_{6a,6b}$ = 12.09 Hz, 1H), 3.52 (H3, H-5, m, 2H), 3.43 (H-2, dd, 1H), 3.29 (OMe, s, 3H), 3.27 (H-4, m, 1H); ^{17}O NMR (54 MHz, D_2O): δ = 11, 0 ppm (D_2O).

[4-^{17}O]-D-glucose (4III). 40 mg of [4-^{17}O]-methyl-α-D-glucopyranoside was dissolved in 1 mL D_2O and 20 grains of H^+ resin. The reaction mixture was heated at 90 °C for three days and neutralized to give syrup form glucose 4III (38 mg, 0.21 mmol, yield: 100%). ^{17}O NMR (54 MHz, D2O): δ = 8, 0 ppm (D_2O).

1.5 Synthesis of [5-^{17}O]-D-glucose

[5-^{17}O]-D-glucose was synthesized in four steps as illustrated in Scheme S5. First, 1,2-O-isopropylidene-D-glucofuranurono-6,3-lactone was oxidized to 1,2-O-

isopropylidene-D-xylo-hexofuranurono-6,3-lactone-5-ulose hydrate (5I) by chromium trioxide. Second, the ^{17}O-labels were introduced to 5I to form [5,5-$^{17}O_2$]-5I via an equilibrium process between the 5-keto and 5,5-gem-diol derivatives in methanol/$H_2{}^{17}O$. Third, [5,5-$^{17}O_2$]-5I was reduced with $NaBH_4$ to 1,2-O-isopropylidene-[5-^{17}O]-α-D-glucofuranose (5II). Fourth, removal of the protecting group led to the formation of [5-^{17}O]-D-glucose (5III). The synthetic details are given below.

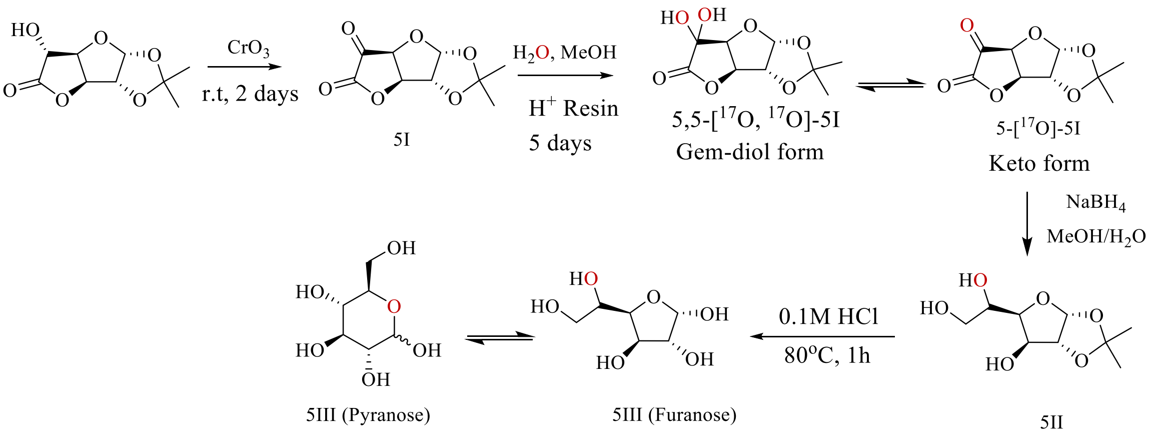

Scheme S5. Synthetic route for preparation of [5-^{17}O]-D-glucose.

1,2-O-isopropylidene-D-xylo-hexofuranurono-6,3-lactone-5-ulose hydrate (5I). CrO_3 (3.5 g, 35 mmol, Sigma Aldrich) was slowly added over 10 minutes to a solution of 1,2-O-isopropylidene-D-glucofuranurono-6,3-lactone (3.8 g, 18 mmol, Alfa Aesar) in ethyl acetate (50 mL) with stirring. The dark red solution was stirred at room temperature for two days to result in a black slurry reaction mixture. The reaction mixture was filtered under vacuum and the black cake was further washed with hot ethyl acetate (15 mL). After decoloring with activated carbon, the clear filtrate was evaporated to dryness. The white solid was dissolved in ethyl acetate (30 mL) and then hexane (10 mL) was added to

precipitate white solid 5I (4.0 g, 17.2 mmol, yield: 96%). ^{1}H NMR (300 MHz, DMSO-d_6): δ = 7.49 and 7.33 (s, *gem*-OH), 5.97 (H-1, d, $J_{1,2}$ = 3.5 Hz, 1H), 4.83 (H-2, d, $J_{1,2}$ = 3.5 Hz, 1H), 4.87 (H-3, d, $J_{3,4}$ = 2.8 Hz, 1H), 4.40 (H-4, d, $J_{3,4}$ = 2.8 Hz, 1H), 1.39 (Me, s, 3H), 1.24 (Me, s, 3H). ^{13}C NMR (75.0 MHz, DMSO-d_6): δ = 172.80, 112.53, 106.72, 93.60, 82.35, 82.19, 82.13, 27.16, and 26.84 ppm.

Exchange process of [5,5-$^{17}O_2$]-5I. 1,2-O-isopropylidene-D-xylo-hexofuranurono-6,3-lactone-5-ulose hydrate (II) (220 mg, 0.950 mmol) was dissolved in 8 mL methanol and 160 μL $H_2{}^{17}O$ (40% ^{17}O atom, purchased from CortecNet), to which 5 grains of Amberlite-IR 120 (H^+) resins were added. The solution was left at room temperature for 5 days, during which ^{17}O NMR was used periodically to monitor the equilibrium process. ^{17}O NMR (67.7 MHz, methanol-d_4): δ = 57, 46, 0 ppm (water) and -22 ppm (methanol).

1,2-O-isopropylidene-α-D-[5-^{17}O]-glucofuranose (5II). To a solution of [5,5-$^{17}O_2$]-5I (220 mg, 0.950 mmol) in 8 mL methanol (directly from Step 2), a solution of sodium borohydride (150 mg, 3.96 mmol, Sigma Aldrich) in methanol (5 mL) was added dropwise. After 40 min, the solution was neutralized with Amberlite-IR 120 (H^+). The residue was treated several times with methanol to remove borate. The white solid (190 mg) was then further recrystallized from ethyl acetate to give 5II (103 mg, 0.47 mmol, yield: 49%). ^{1}H NMR (600 MHz, methanol-d_4,): δ = 5.87 (H-1,d, $J_{1,2}$ = 3.6 Hz, 1H), 4.48 (H-2, dd, $J_{1,2}$ = 3.6 Hz, $J_{2,3}$ < 0.1Hz, 1H), 4.21 (H-3, dd, $J_{3,4}$ = 2,62 Hz, $J_{2,3}$ < 0.1 Hz, 1H), 4.02 (H-4, dd, $J_{3,4}$ = 2.62 Hz, $J_{4,5}$ = 8.38 Hz, 1H), 3.89 (H-5, ddd, $J_{4,5}$ = 8.38 Hz, $J_{5,6b}$ = 6.0 Hz, $J_{5,6a}$ = 3.16 Hz, 1H), 3.76 (H-6a, dd, $J_{5,6a}$ = 3.16 Hz, $J_{6a,6b}$ = 11.53 Hz, 1H), 3.60 (H-

6b,dd, $J_{5,6b}$ = 6.0 Hz, $J_{6a,6b}$ = 11.53 Hz, 1H), 1.45 (Me, s, 3H), 1.31 (Me, s,3H); ^{13}C NMR (150 MHz, methanol-d_4,): δ = 112.72, 106.15, 86.44, 81.13, 75.32, 70.51, 65.71, 27.30, 26.03 ppm. ^{17}O NMR (81.4 MHz, methanol-d_4): δ = 3, -37 ppm (MeOD).

[5-^{17}O]-D-glucose (5III). A mixture of 1,2-O-isopropylidene-α-D-[5-^{17}O] glucofuranose (5II) (103 mg, 0.470 mmol) and 4 mL of 0.1 M HCl was heated at 80 °C for 1 h. The solution was then neutralized with AmberLite A26 OH^- resin. The solution was evaporated to dryness to give syrup 5III (80.0 mg, 0.44 mmol, yield: 94%). ^{1}H NMR (300 MHz, D_2O): δ = 5.32 (αH-1), 4.74 (βH-1), multiple peaks from 3.5-4 ppm. ^{13}C NMR (75.0 MHz, D_2O): δ = 96.05 (αH-1), 92.18 (βH-1), 75.89, 75.70, 74.08, 72.72, 71.40, 69.58, 60.72, 60.56 ppm. ^{17}O NMR (67.7 MHz, D_2O): δ = 62, 0 ppm (D_2O).

1.6 Synthesis of [6-^{17}O]-D-glucose

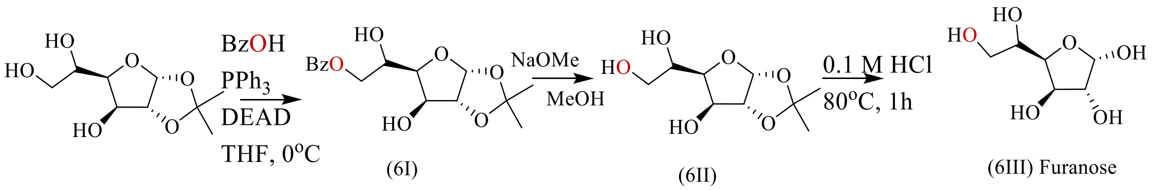

Scheme S6. Synthetic route for preparation of [6-^{17}O]-D-glucose by Mitsunobu reaction.

[6-^{17}O]-Benzoyl-1,2-O-isopropylidene-α-D-glucofuranose (6I). In a 100 mL flask added with 1,2-O-isopropylidene-α-D-glucofuranose (330 mg, 1.50 mmol, Sigma Aldrich) and 24 mL anhydrous THF. After fully dissolved, ^{17}O-labeled BzOH (193 mg, 1.58 mmol), PPh_3 (786 mg, 3.00 mmol, Sigma-Aldrich) was added. Then it was treated with diethyl azodicarboxylate (0.480 mL, 3.00 mmol, Alfa Aesar) dropwise in 0 °C with ice-water bath. The mixture was then stirred for 48 h at room temperature. TLC plate

analysis [5:95, (v/v) MeOH/CH_2Cl_2] showed the reaction was complete. The mixture was evaporated, and the crude residue was purified by column chromatography (9:91 (v/v) MeOH/CH_2Cl_2) to afford the white solid 6I (400 mg, 1.23 mmol, yield: 82%). ^{1}H NMR (300 MHz, DMSO –*d₆*): δ = 7.55-8.01 (m, 5H, Ar-H), 5.82 (H-1, d, 1H, $J_{1,2}$ = 3.6 Hz), 5.29 (OH, d, 1H, J = 5.0 Hz), 5.20 (OH, d, 1H, J = 5.7 Hz), 4.43 (H-6, dd, 1H, $J_{5,6}$ = 1.9 Hz, $J_{6,6'}$ = 11.3 Hz), 4.42 (H-2, d, 1H), 4.22 (H-6', dd, 1H, $J_{5,6'}$ = 5.5 Hz, $J_{6,6'}$ = 11.3 Hz), 4.08 (H-4, dd, 1H, $J_{3,4}$ = 2.3 Hz, $J_{4,5}$ = 4.9 Hz), 4.04 (H-5, m, 1H), 4.02 (H-3, d, 1H), 1.37 (s, 3H, CH_3), 1.27 (s, 3H, CH_3).

1,2-O-Isopropylidene-α-D-[6-^{17}O]-glucofuranose (6II). A solution of NaOMe in MeOH (~ 0.5 M, ~ 4 mL) was added to [6-^{17}O]-benzoyl-1,2-O-isopropylidene-α-D-glucofuranose (6I) (400 mg, 1.23 mmol). The mixture was stirred for 1 h until it was shown by TLC analysis [5:95, (v/v) MeOH/CH_2Cl_2] that the reaction was complete. After that, the reaction mixture was neutralized by about 3 mL Amberlite H^+ (1.8 meq/mL) and was filtered and concentrated. Later the residue was purified by another column chromatography [7:93, (v/v) MeOH/CH_2Cl_2] to give the white solid product 6II (257 mg, 1.17 mmol, yield: 94%). ^{1}H NMR (600 MHz, methanol-d_4): δ = 5.87 (H-1, d, $J_{1,2}$ = 3.6 Hz, 1H), 4.48 (H-2, dd, $J_{1,2}$ = 3.6 Hz, $J_{2,3}$<0.1 Hz, 1H), 4.21 (H-3, dd, $J_{3,4}$ = 2.62 Hz, $J_{2,3}$ < 0.1 Hz, 1H), 3.90 (H-4, dd, $J_{3,4}$ = 2.62 Hz, $J_{4,5}$= 8.38 Hz, 1H), 3.75 (H-5, ddd, $J_{4,5}$ = 8.38 Hz, $J_{5,6b}$ = 6.0 Hz, $J_{5,6a}$ = 3.16 Hz, 1H), 3.61 (H-6a, dd, $J_{5,6a}$ = 3.16 Hz, $J_{6a,6b}$ = 11.53 Hz, 1H), 3.57 (H-6b, dd, $J_{5,6b}$ = 6.0 Hz, $J_{6a,6b}$ = 11.53 Hz, 1H); ^{13}C NMR (150 MHz, methanol-d_4): δ = 112.67, 106.33, 86.50, 81.28, 75.35, 70.34, 65.13, 26.99, 26.28 ppm. ^{17}O NMR (54 MHz, methanol-d_4): δ = -15 ppm, -37 ppm (MeOD).

[6-^{17}O]-D-glucose (6III). A mixture of 1,2-O-isopropylidene-α-D-[6-^{17}O]-glucofuranose (6II) (257 mg, 1.17 mmol) and 5 mL of 0.1 M HCl was heated at 80 ^{o}C for 1 h. The solution was then neutralized with OH^- resin. The solution was evaporated to dryness to give syrup 6III (180 mg, 1.00 mmol, yield: 85%). ^{1}H NMR (300 MHz, D_2O): δ = 5.32 (αH-1), 4.74 (βH-1), multiple peaks from 3.5-4 ppm. ^{13}C NMR (75.0 MHz, D_2O): δ = 96.05 (αH-1), 92.18 (βH-1), 75.89, 75.70, 74.08, 72.72, 71.40, 69.58, 60.72, 60.56 ppm. ^{17}O NMR (67.7 MHz, D_2O): δ = -7, 0 ppm (D_2O).

1.7 Summary of ^{17}O NMR data obtained for reaction intermediates and final products

Table S1. Summary of ^{17}O NMR chemical shifts observed for reaction intermediates and final products.

Compound	δ^{17}O (ppm)	Compound	δ^{17}O (ppm)
**1I**[a]	46.5 (β form), 36.3 (α form)	4III[b]	8.3
**2I**[a]	151.5[d], 341.5[e]	[5,5-$^{17}O_2$]-5I[f]	57.1, 46.2
**2II**[b]	10.1	[5,5-$^{17}O_2$]-5I[g]	562.1
**3I**[a]	150.8[d], 340.1[e]	5II[c]	3.2
**3II**[c]	0.6	5III[b]	62.0
**3III**[b]	12.8	6II[c]	-14.6
**4I**[a]	148.9[d], 335.5[e]	6III[b]	-7.6
**4II**[b]	11.0		

[a] In $CDCl_3$, referenced to external H_2O at 0.0 ppm. [b] In H_2O, referenced to H_2O at 0.0 ppm. [c] In MeOD, referenced to external H_2O at 0.0 ppm. [d] PhCOOR shift. [e] PhCOOR shift. [f] Gem-diol form. [g] Ketone form.

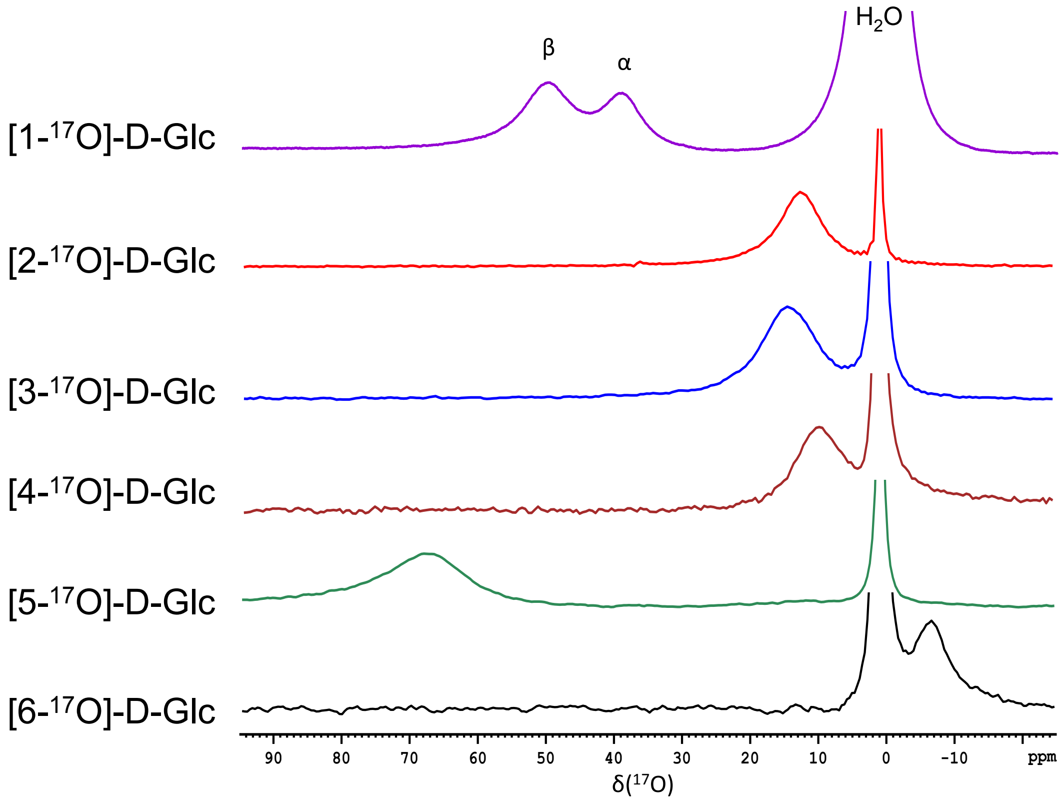

Figure S1. Solution ^{17}O NMR spectra of the six glucose compounds synthesized in this study. All experiments were performed at 11.7 T for aqueous solutions of D-glucose at 90 °C.

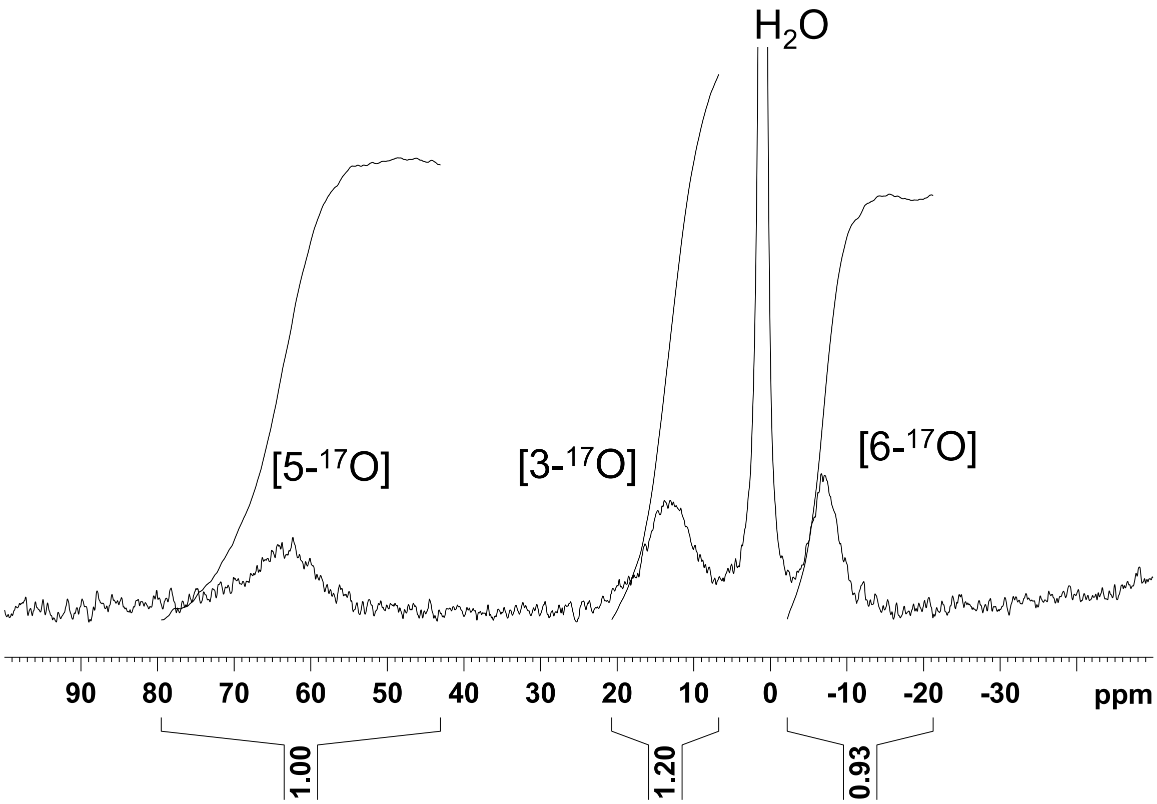

Figure S2. Solution ^{17}O NMR spectrum obtained at 14.1 T for an aqueous solution of [3/5/6-^{17}O]-D-glucose at 90 °C.

2. Solid-state ^{17}O NMR

2.1 Data acquisition parameters for 1D ^{17}O NMR experiments

Table S2. NMR data acquisition parameters used in the solid-state ^{17}O NMR experiments for site-specifically ^{17}O-labeled glucose compounds at two magnetic fields.

Compound	MAS at 21.1 T	Static at 21.1 T	Static at 14.1 T
[1-^{17}O]Glc	D1 = 5 s; NS = 16000	D1 = 5 s; NS = 16000	D1 = 10 s; NS = 6273
[2-^{17}O]Glc	D1 = 10 s; NS = 6000	D1 = 10 s; NS = 8000	D1 = 10 s; NS = 6923
[3-^{17}O]Glc	D1 = 10 s; NS = 8752	D1 = 5 s; NS = 10500	D1 = 10 s; NS = 16496
[4-^{17}O]Glc	D1 = 5 s; NS = 16000	D1 = 5 s; NS = 16000	D1 = 10 s; NS = 14406
[5-^{17}O]Glc	D1 = 5 s; NS = 44000	D1 = 5 s; NS = 30500	D1 = 2 s; NS = 85330
[6-^{17}O]Glc	D1 = 5 s; NS = 12000	D1 = 5 s; NS = 5000	D1 = 10 s; NS = 8793

2.2 Signal positions in ^{17}O 3QMAS spectra (Figure 7)

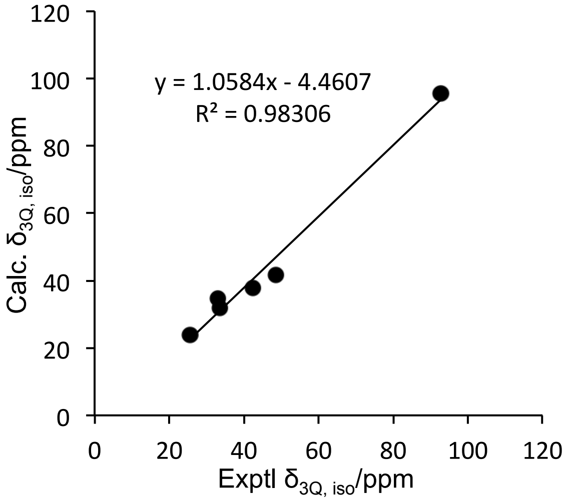

Figure S3. Comparison between experimental and calculated signal positions in ^{17}O 3QMAS spectra for [2-^{17}O]-D-glucose and [3,5,6-$^{17}O_3$]-D-glucose at 18.8 T. $\delta_{3Q,iso}$ is calculated according to the following equation: $\delta_{3Q,iso} = \delta_{iso} + \frac{3}{850}\left(1 + \frac{\eta_Q}{3}\right)\left(\frac{C_Q}{\nu_0}\right)^2$. The root mean square error of the data is 3.1 ppm.

3. Solid-state ^{13}C NMR characterization of solid samples

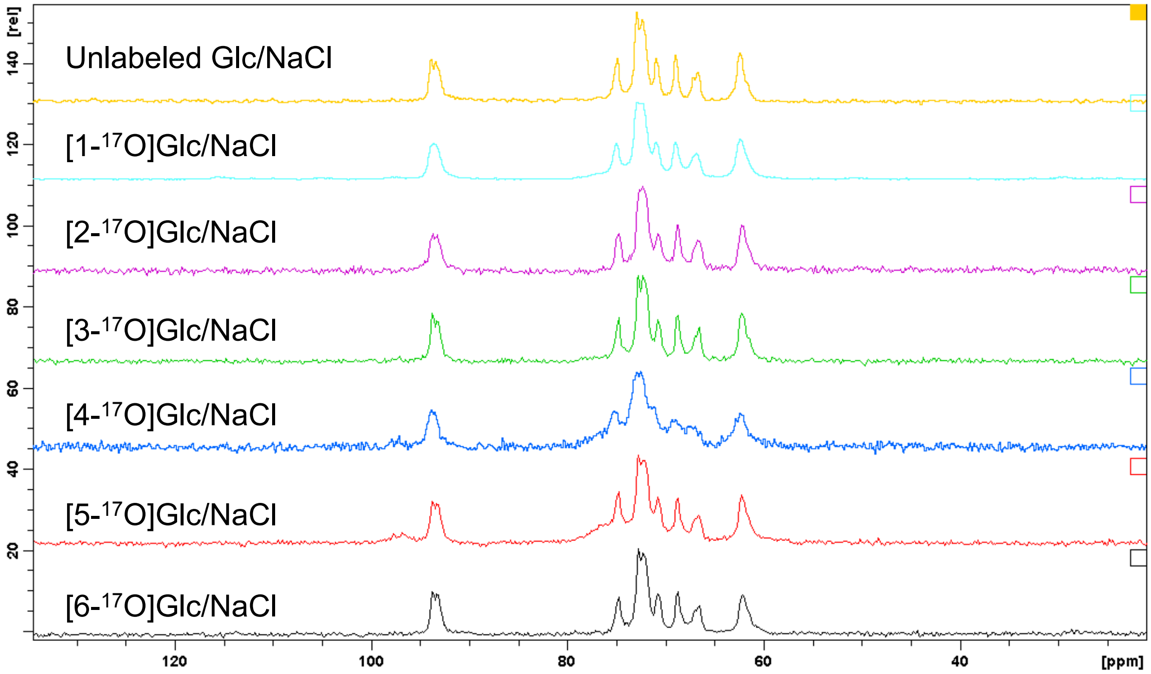

Figure S4. Experimental 12-kHz ^{13}C CP/MAS NMR spectra of the glucose compounds examined in this study. All experiments were performed at 14.1 T.

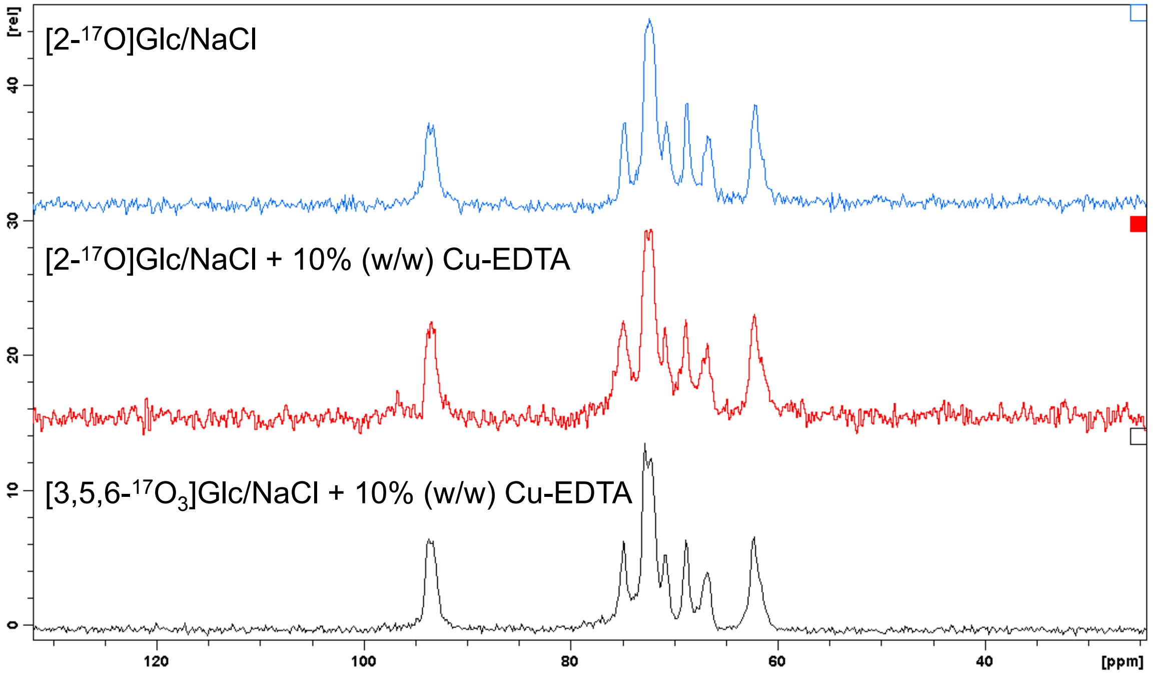

Figure S5. Comparison of ^{13}C CP/MAS NMR spectra obtained for regular and Cu-EDTA doped glucose samples. All experiments were performed at 14.1 T with a spinning frequency of 12 kHz.

4. GIPAW DFT results

4.1 Hydrogen bonding interactions around water molecules

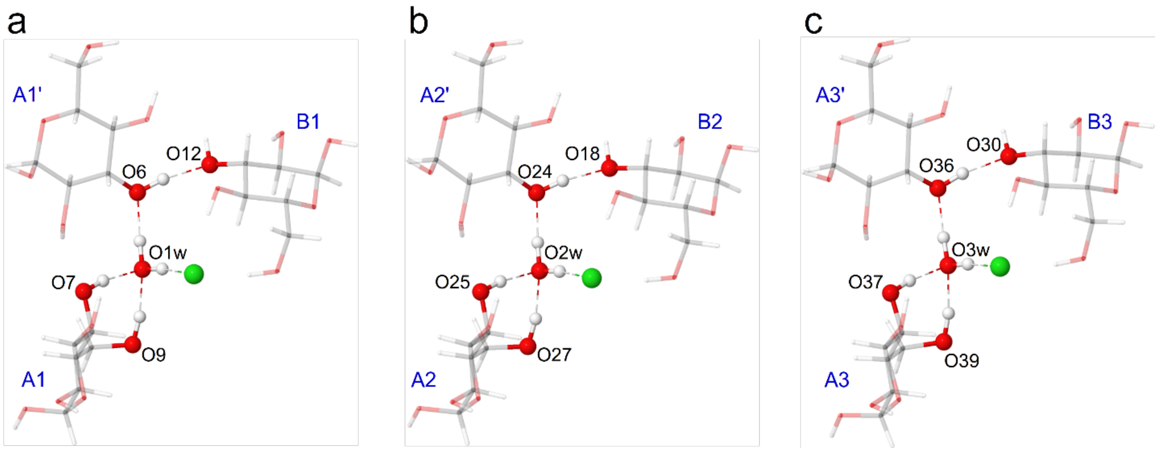

Figure S6: Hydrogen bonding environments of the three water molecules in the asymmetric unit cell of the D-glucose/NaCl/H_2O (2/1/1) cocrystal. A prime in the label indicates glucose molecules related by the symmetry transformation (y-x, -x, z-1/3) to the basic molecule labeled without a prime. The water oxygen O2w was only bonded to a single hydrogen atom in the original crystal structure (CCDC 1281434.cif). An additional hydrogen atom was added to oxygen O2w such that after geometry optimization using DFT, the hydrogen-bonding environment of all three water molecules is consistent.

4.2 Complete GIPAW DFT results on ^{17}O CS and QC tensors

Table S3. GIPAW DFT results on ^{17}O CS and QC tensors for the six crystallograpically distinct glucose molecules in the asymmetric unit of α-D-glucose/NaCl/H_2O cocrystal.

Mol	Atom[a]	Atom[b]	Calc	σ_{iso}/ppm	σ_{11}/ppm	σ_{22}/ppm	σ_{33}/ppm	C_Q/MHz	η_Q
A1	O1	O4	PBE	234.4	193.4	226.1	283.6	–9.166	0.84
			rPBE	237.3	195.9	233.0	283.1	–9.179	0.88
			D2[c]	261.9	220.1	260.8	304.7	–9.163	0.83
			D2[d]	245.8	204.4	241.3	291.8	–9.174	0.86
	O2	O5	PBE	271.7	240.6	275.1	299.5	9.965	0.98
			rPBE	274.2	243.3	277.5	301.8	10.160	0.95
			D2[c]	298.9	268.8	305.1	322.9	–9.858	0.99
			D2[d]	282.3	251.6	285.7	309.7	10.016	0.97
	O3	O6	PBE	248.6	229.2	241.5	275.2	9.514	0.99
			rPBE	250.9	234.9	243.2	274.6	9.574	0.99
			D2[c]	282.2	263.6	276.1	307.1	–9.662	0.98
			D2[d]	261.4	243.6	254.4	286.1	9.590	0.99
	O4	O7	PBE	260.6	229.2	256.0	296.6	9.915	0.96
			rPBE	264.7	234.1	261.2	298.9	10.177	0.92
			D2[c]	290.1	261.2	291.6	317.4	9.808	0.98
			D2[d]	272.3	242.1	269.3	305.6	10.008	0.94
	O5	O8	PBE	199.4	166.8	189.2	242.3	10.950	0.86
			rPBE	200.7	168.2	191.1	242.9	11.055	0.86
			D2[c]	223.5	193.9	214.2	262.3	10.731	0.91
			D2[d]	209.3	177.7	199.8	250.5	10.923	0.87
	O6	O9	PBE	281.0	248.1	269.4	325.3	9.880	0.90
			rPBE	284.0	253.8	273.0	325.3	10.042	0.89
			D2[c]	314.5	287.5	306.2	349.8	9.593	0.98
			D2[d]	292.9	262.9	282.5	333.3	9.874	0.92
A2	O1	O22	PBE	236.5	194.2	228.3	287.1	–9.196	0.89
			rPBE	239.1	196.2	235.1	286.0	–9.205	0.92
			D2[c]	263.4	219.9	263.5	306.9	–9.191	0.87
			D2[d]	247.8	204.4	243.8	295.2	–9.201	0.91
	O2	O23	PBE	272.3	242.1	275.8	298.9	10.060	0.96
			rPBE	274.5	244.9	277.9	300.8	10.246	0.94
			D2[c]	299.2	269.7	305.8	322.1	–9.877	0.99
			D2[d]	282.9	253.0	286.6	309.1	10.108	0.96
	O3	O24	PBE	249.0	228.9	242.2	276.1	9.579	0.99
			rPBE	251.8	234.7	244.4	276.4	9.650	0.99
			D2[c]	282.8	263.4	276.7	308.2	–9.702	0.98
			D2[d]	261.8	243.2	255.2	286.9	9.649	0.99

	O4	O25	PBE	260.2	228.5	255.5	296.5	9.923	0.96
			rPBE	264.5	233.9	260.8	298.9	10.176	0.93
			D2[c]	289.8	260.9	291.3	317.3	9.805	0.98
			D2[d]	272.1	241.8	268.9	305.6	10.019	0.94
	O5	O26	PBE	198.4	166.2	188.9	240.0	10.910	0.87
			rPBE	199.8	167.5	190.6	241.4	10.991	0.86
			D2[c]	222.5	193.2	213.5	260.8	10.675	0.92
			D2[d]	208.3	177.1	199.3	248.5	10.882	0.88
	O6	O27	PBE	281.3	251.2	268.8	324	9.982	0.90
			rPBE	284.7	257.9	271.8	324.4	10.167	0.89
			D2[c]	315.3	290.4	306.4	348.9	9.695	0.98
			D2[d]	293.3	265.7	282.1	332.2	9.979	0.92
A3	O1	O34	PBE	236.6	195.2	229.3	285.4	–9.168	0.86
			rPBE	239.4	197.8	236.7	283.7	–9.187	0.90
			D2[c]	264.3	221.2	264.6	307.0	–9.151	0.85
			D2[d]	248.1	205.8	244.6	293.8	–9.171	0.89
	O2	O35	PBE	271.8	241.1	274.4	299.8	9.947	0.98
			rPBE	274.6	244.3	277.5	301.9	10.149	0.95
			D2[c]	299.3	269.5	305.0	323.3	–9.825	0.99
			D2[d]	282.5	252.1	285.4	310.0	10.004	0.97
	O3	O36	PBE	248.8	229.0	241.7	275.6	9.571	0.98
			rPBE	251.0	235.0	242.8	275.3	9.621	0.98
			D2[c]	282.6	263.7	275.9	308.2	–9.659	0.99
			D2[d]	261.6	243.5	254.5	286.8	9.636	0.98
	O4	O37	PBE	260.6	228.7	256.4	296.7	9.922	0.96
			rPBE	264.8	233.5	262.0	299.0	10.207	0.92
			D2[c]	290.2	260.7	292.2	317.5	9.807	0.97
			D2[d]	272.5	241.8	269.9	305.8	10.013	0.94
	O5	O38	PBE	199.5	167.4	188.8	242.3	10.960	0.86
			rPBE	200.7	168.8	190.6	242.8	11.075	0.86
			D2[c]	223.9	193.9	215.0	262.7	10.738	0.92
			D2[d]	209.5	178.2	199.7	250.7	10.937	0.88
	O6	O39	PBE	280.9	248.5	269.4	324.9	9.834	0.91
			rPBE	283.9	254.1	272.9	324.6	9.999	0.90
			D2[c]	314.4	287.9	306.1	349.3	9.570	0.99
			D2[d]	292.7	263.2	282.3	332.7	9.839	0.92
B1	O1	O10	PBE	238.4	196.0	231.3	288.0	–9.162	0.93
			rPBE	240.9	198.6	238.6	285.4	–9.173	0.97
			D2[c]	265.6	221.0	267.6	308.2	–9.128	0.91
			D2[d]	249.7	205.9	247.1	296.2	–9.155	0.95
	O2	O11	PBE	269.8	243.5	272.2	293.8	9.964	0.97
			rPBE	273.2	246.9	275.4	297.2	10.159	0.94
			D2[c]	297.1	271.7	301.3	318.1	–9.805	0.99
			D2[d]	280.8	254.5	283.0	304.8	10.012	0.96

	O3	O12	PBE	241.8	209.2	246.9	269.4	10.110	0.89
			rPBE	245.7	215.0	250.5	271.5	10.217	0.88
			D2[c]	276.9	245.5	286.9	298.2	9.846	0.97
			D2[d]	255.7	224.3	262.1	280.7	10.115	0.90
	O4	O13	PBE	253.2	219.5	245.2	294.9	10.220	0.92
			rPBE	257.0	223.6	249.9	297.4	10.349	0.90
			D2[c]	283.3	252.3	281.9	315.8	9.897	0.97
			D2[d]	265.7	233.1	260.2	303.9	10.233	0.91
	O5	O14	PBE	201.5	171.5	190.2	242.8	10.890	0.89
			rPBE	202.1	171.2	191.5	243.6	10.988	0.88
			D2[c]	224.4	195.5	215.3	262.6	10.643	0.94
			D2[d]	211.1	181.6	200.7	250.8	10.864	0.90
	O6	O15	PBE	265.3	218.9	253.4	323.6	9.806	0.92
			rPBE	268.4	226.3	254.5	324.4	10.019	0.90
			D2[c]	299.9	262.6	289.6	347.5	9.526	1.00
			D2[d]	277.4	235.1	265.8	331.5	9.797	0.93
B2	O1	O16	PBE	237.0	196.7	229.9	284.5	–9.109	0.89
			rPBE	240.0	200.1	237.8	282.1	–9.129	0.93
			D2[c]	264.7	222.4	265.8	305.9	–9.084	0.88
			D2[d]	248.5	207.1	245.4	292.9	–9.109	0.91
	O2	O17	PBE	269.3	241.8	271.7	294.5	9.870	0.99
			rPBE	273.1	245.6	275.4	298.4	10.071	0.96
			D2[c]	297.0	270.9	300.8	319.2	–9.784	0.98
			D2[d]	280.3	253.1	282.3	305.6	9.929	0.97
	O3	O18	PBE	241.0	209.2	245.1	268.6	10.010	0.90
			rPBE	244.0	214.6	247.5	270.0	10.072	0.89
			D2[c]	275.8	245.1	284.8	297.6	9.753	0.98
			D2[d]	254.7	224.0	260.0	280.0	10.018	0.91
	O4	O19	PBE	253.6	219.9	246.3	294.7	10.210	0.91
			rPBE	257.6	223.9	251.6	297.3	10.346	0.90
			D2[c]	283.8	252.6	283.3	315.5	9.888	0.96
			D2[d]	266.1	233.4	261.4	303.5	10.220	0.90
	O5	O20	PBE	202.7	171.7	190.8	245.4	10.920	0.89
			rPBE	203.1	171.6	192.0	245.6	11.039	0.88
			D2[c]	225.5	195.8	216.2	264.5	10.696	0.94
			D2[d]	212.1	181.9	201.4	253.1	10.901	0.90
	O6	O21	PBE	264.2	215.3	253.9	323.3	9.673	0.92
			rPBE	266.7	221.5	255.6	322.9	9.839	0.90
			D2[c]	298.8	259.4	289.9	347.0	9.420	1.00
			D2[d]	276.5	232.0	266.3	331.1	9.670	0.93
B3	O1	O28	PBE	236.3	195.0	228.0	286.0	–9.153	0.91
			rPBE	239.2	197.4	234.8	285.3	–9.159	0.94
			D2[c]	263.5	220.6	263.7	306.2	–9.140	0.89
			D2[d]	247.8	205.2	243.8	294.3	–9.153	0.93

O2	O29	PBE	269.3	242.8	272.1	292.9	9.993	0.97
		rPBE	272.3	245.7	274.9	296.2	10.186	0.94
		D2[c]	296.1	270.8	300.7	316.8	–9.843	0.99
		D2[d]	280.1	253.7	282.6	303.9	10.040	0.96
O3	O30	PBE	241.4	208.9	245.1	270.2	10.020	0.90
		rPBE	245.2	214.6	248.7	272.4	10.144	0.89
		D2[c]	276.2	244.9	284.8	299.1	9.777	0.98
		D2[d]	255.2	223.9	260.1	281.6	10.038	0.91
O4	O31	PBE	252.9	219.6	244.2	294.9	10.200	0.92
		rPBE	256.2	223.4	248.2	297.1	10.313	0.90
		D2[c]	282.8	252.1	280.6	315.6	9.875	0.97
		D2[d]	265.3	233.0	259.0	303.8	10.207	0.91
O5	O32	PBE	201.1	170.4	190.6	242.4	10.880	0.88
		rPBE	202.1	170.5	192.6	243.1	10.974	0.88
		D2[c]	224.2	195.8	214.8	262.1	10.636	0.93
		D2[d]	210.8	181.0	201.1	250.3	10.849	0.89
O6	O33	PBE	265.7	220.0	252.5	324.7	9.891	0.91
		rPBE	269.4	228.7	253.7	326.0	10.116	0.89
		D2[c]	300.6	264.1	289.3	348.5	9.585	0.99
		D2[d]	278.1	236.5	265.2	332.5	9.875	0.92

[a]Atomic numbering according to Scheme 1. [b]Atomic numbering in the original crystal structure (CCDC 1281434.cif). [c]rPBE and D2 with $d = 3.25$. [d]rPBE and D2 with $d = 5.0$.

4.3 Complete GIPAW DFT results on ^{13}C isotropic magnetic shielding constants

Table S4. GIPAW DFT results on ^{13}C isotropic magnetic shielding constants for the six crystallograpically distinct glucose molecules in the asymmetric unit of α-D-glucose/NaCl/H_2O cocrystal.

Mol	Atom[a]	Atom[b]	Calc	σ_{iso}/ppm	Mol	Atom[a]	Atom[b]	Calc	σ_{iso}/ppm
A1	C1	C1	PBE	72.6	B1	C1	C7	PBE	72.0
			rPBE	74.1				rPBE	73.5
			D2[c]	84.7				D2[c]	83.9
			D2[d]	78.1				D2[d]	77.4
	C2	C2	PBE	97.3		C2	C8	PBE	96.8
			rPBE	99.1				rPBE	98.7
			D2[c]	109.2				D2[c]	108.9
			D2[d]	102.9				D2[d]	102.5
	C3	C3	PBE	94.3		C3	C9	PBE	94.3
			rPBE	95.9				rPBE	96.1
			D2[c]	107.6				D2[c]	107.7
			D2[d]	100.4				D2[d]	100.5
	C4	C4	PBE	101.2		C4	C10	PBE	102.5
			rPBE	102.1				rPBE	103.7
			D2[c]	112.7				D2[c]	114.1
			D2[d]	106.3				D2[d]	107.6
	C5	C5	PBE	97.5		C5	C11	PBE	98.0
			rPBE	99.0				rPBE	99.1
			D2[c]	109.9				D2[c]	109.6
			D2[d]	103.0				D2[d]	103.2
	C6	C6	PBE	108.8		C6	C12	PBE	109.3
			rPBE	110.3				rPBE	110.1
			D2[c]	123.2				D2[c]	123.3
			D2[d]	114.6				D2[d]	114.9
A2	C1	C19	PBE	72.5	B2	C1	C13	PBE	72.0
			rPBE	74.1				rPBE	73.5
			D2[c]	84.5				D2[c]	84.1
			D2[d]	78.0				D2[d]	77.5
	C2	C20	PBE	97.0		C2	C14	PBE	97.0
			rPBE	98.8				rPBE	98.8
			D2[c]	109.2				D2[c]	108.9
			D2[d]	102.7				D2[d]	102.6
	C3	C21	PBE	94.3		C3	C15	PBE	94.5
			rPBE	95.9				rPBE	96.4
			D2[c]	107.6				D2[c]	108.0
			D2[d]	100.3				D2[d]	100.8
	C4	C22	PBE	101.0		C4	C16	PBE	102.6

			rPBE	102.0				rPBE	103.8
			D2[c]	112.7				D2[c]	114.1
			D2[d]	106.1				D2[d]	107.7
	C5	C23	PBE	97.4		C5	C17	PBE	98.1
			rPBE	99.0				rPBE	99.2
			D2[c]	109.7				D2[c]	109.7
			D2[d]	102.9				D2[d]	103.2
	C6	C24	PBE	108.0		C6	C18	PBE	110.1
			rPBE	109.2				rPBE	111.2
			D2[c]	122.6				D2[c]	124.1
			D2[d]	113.8				D2[d]	115.8
A3	C1	C31	PBE	72.3	B3	C1	C25	PBE	72.3
			rPBE	73.9				rPBE	73.9
			D2[c]	84.5				D2[c]	84.2
			D2[d]	77.9				D2[d]	77.8
	C2	C32	PBE	97.7		C2	C26	PBE	96.3
			rPBE	99.4				rPBE	98.3
			D2[c]	109.7				D2[c]	108.4
			D2[d]	103.3				D2[d]	102.1
	C3	C33	PBE	93.9		C3	C27	PBE	94.5
			rPBE	95.6				rPBE	96.1
			D2[c]	107.3				D2[c]	107.9
			D2[d]	100.1				D2[d]	100.7
	C4	C34	PBE	101.1		C4	C28	PBE	102.5
			rPBE	102.1				rPBE	103.7
			D2[c]	112.6				D2[c]	114.2
			D2[d]	106.2				D2[d]	107.6
	C5	C35	PBE	97.5		C5	C29	PBE	98.1
			rPBE	99.0				rPBE	99.3
			D2[c]	110.0				D2[c]	109.6
			D2[d]	103.0				D2[d]	103.2
	C6	C36	PBE	108.6		C6	C30	PBE	109.4
			rPBE	109.9				rPBE	110.3
			D2[c]	122.9				D2[c]	123.5
			D2[d]	114.3				D2[d]	115.1

[a]Atomic numbering according to Scheme 1. [b]Atomic numbering in the original crystal structure (CCDC 1281434.cif). [c]rPBE and D2 with d = 3.25. [d]rPBE and D2 with d = 5.0.

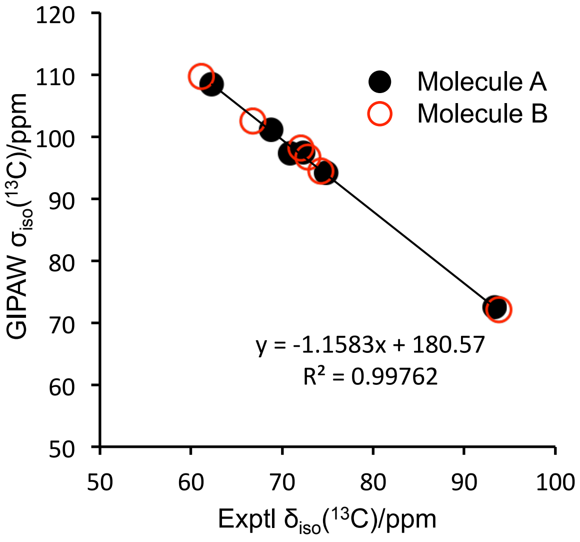

Figure S7. Comparison between GIPAW DFT computed ^{13}C isotropic magnetic shielding constants and experimental ^{13}C isotropic chemical shifts for Molecules A and B in the D-glucose "dimer". The root mean square error of the data is 0.5 ppm.

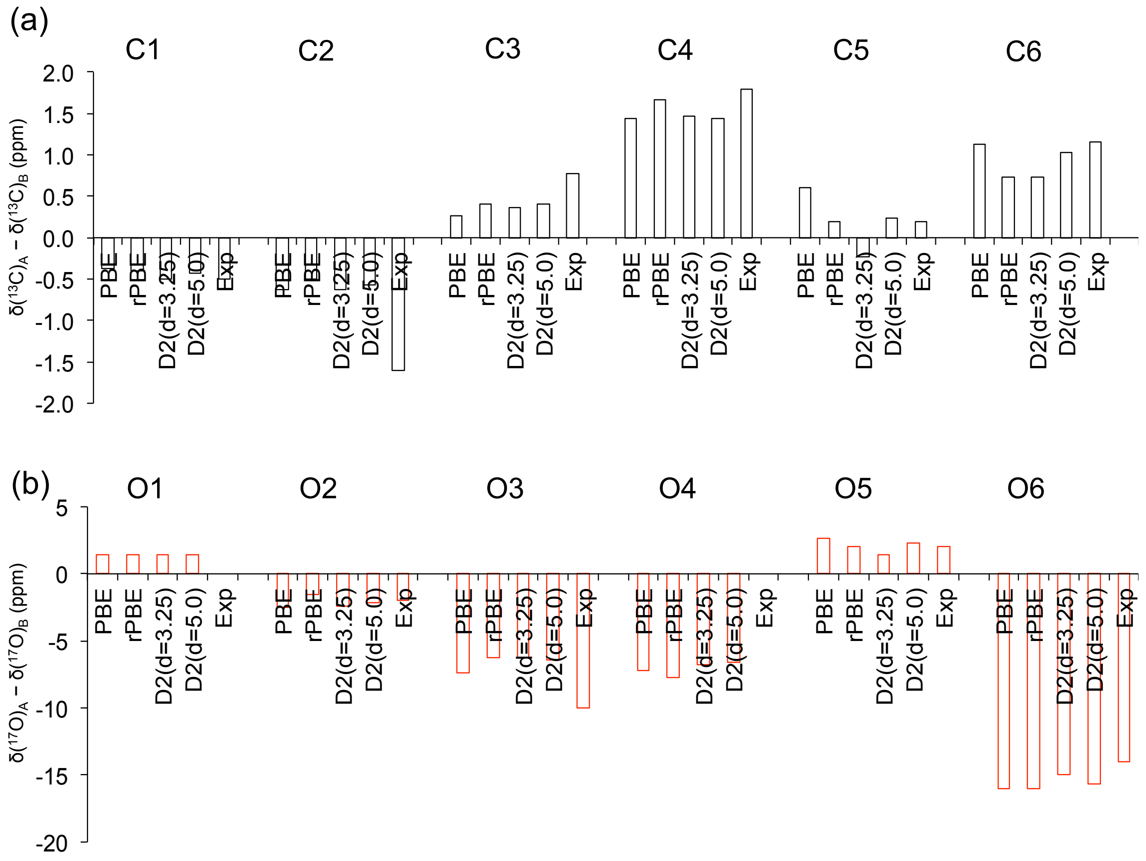

Figure S8. Comparison between observed and all GIPAW DFT calculated ^{13}C (a) and ^{17}O (b) chemical shift differences between Molecules A and B in α-D-glucose/NaCl/H_2O cocrystal. In (b), because the line width observed in the 3Q isotropic dimension for the O2 and O5 3QMAS signals was about 5 ppm, the upper limit of any potential signal splittings for O2 and O5 was estimated to be 2 ppm.

Appendix: Supplementary solution ^{1}H, ^{13}C, and ^{17}O NMR spectra

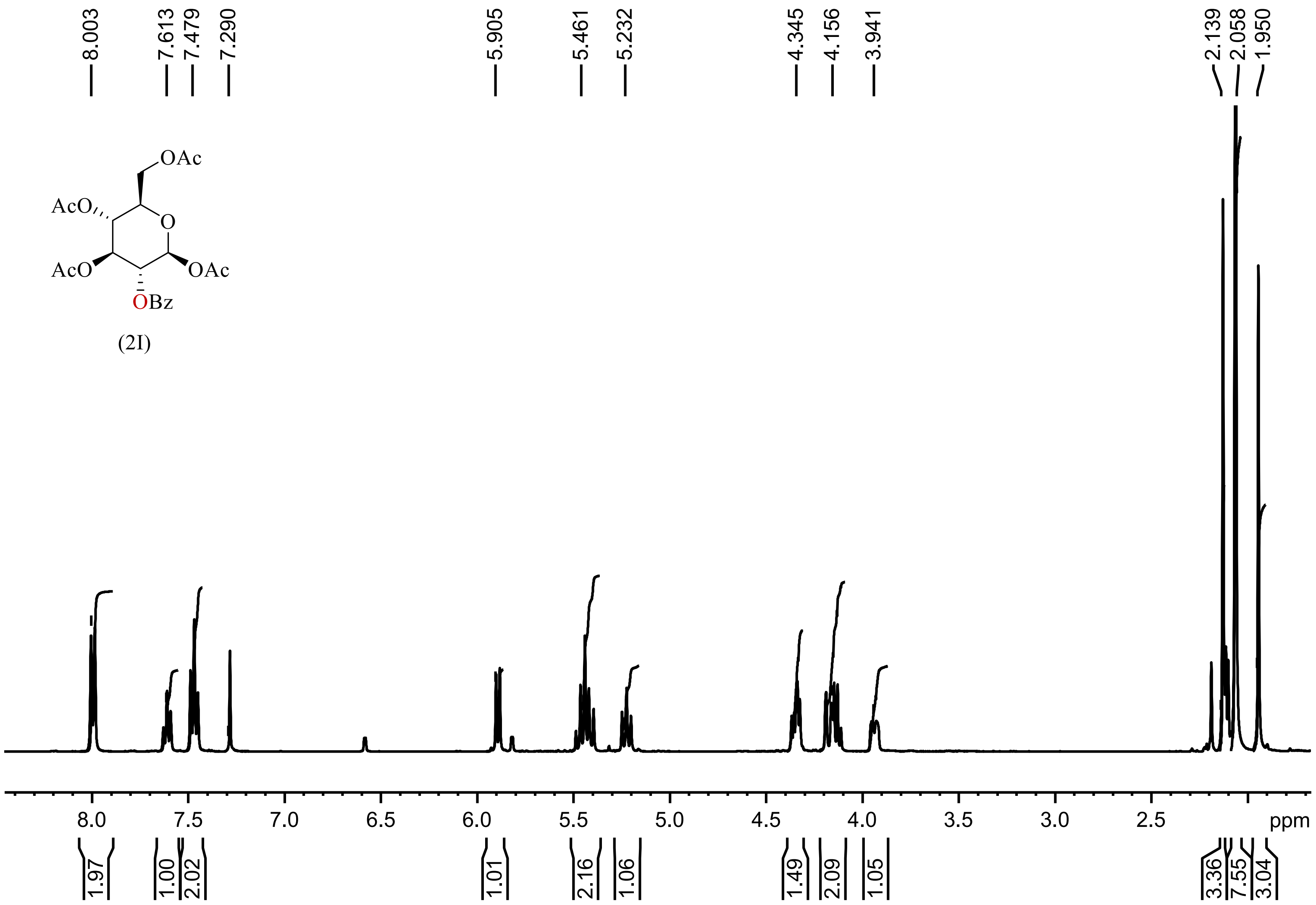

Figure S9. ^{1}H NMR of 1,3,4,6-Tetra-O-acetyl, [2-^{17}O]-benzoyl-beta-D-mannopyranose **(2I)** in $CDCl_3$ at 9.4 T.

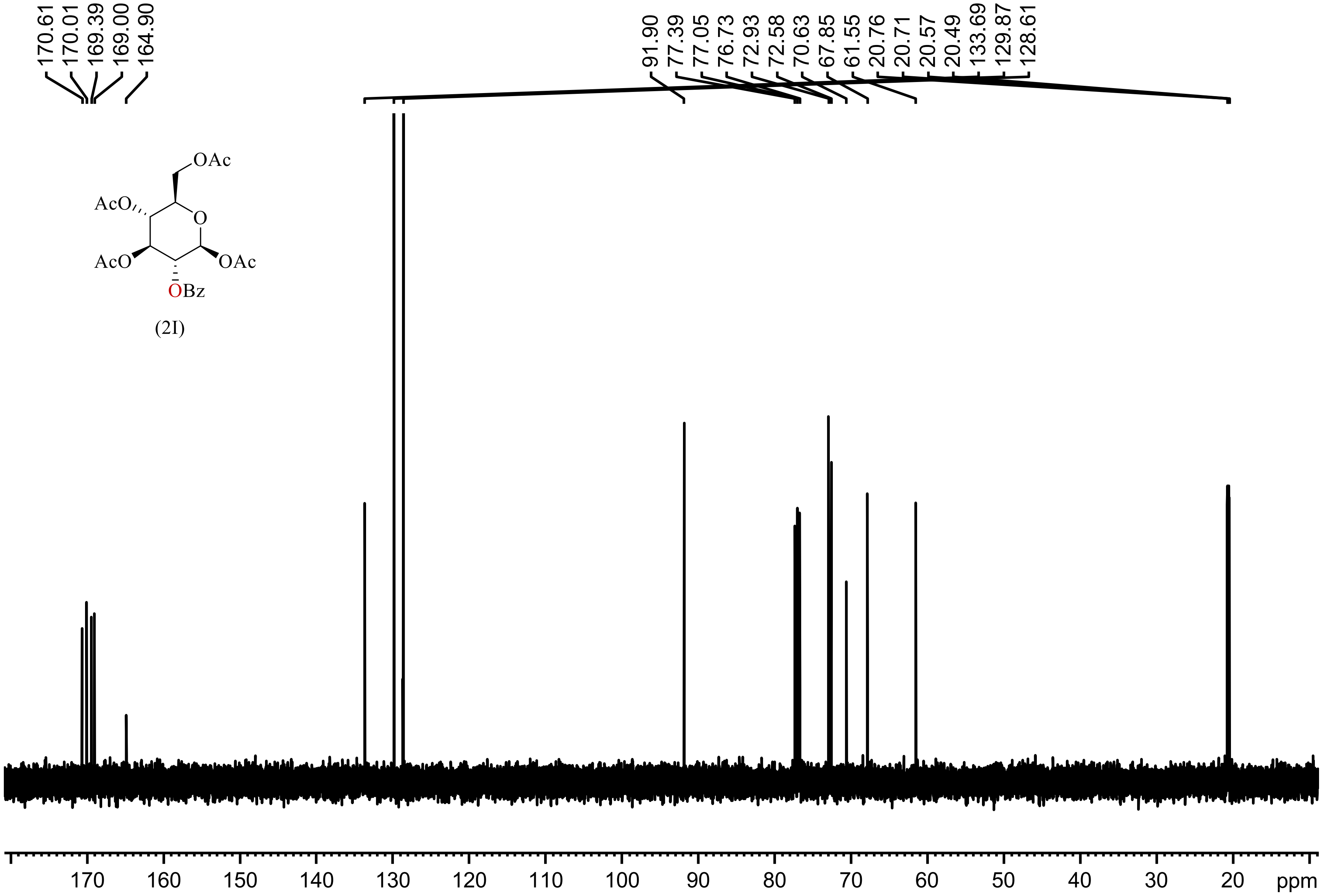

Figure S10. ^{13}C NMR of 1,3,4,6-Tetra-O-acetyl, [2-^{17}O]-benzoyl-beta-D-mannopyranose **(**2I**)** in $CDCl_3$ at 9.4 T.

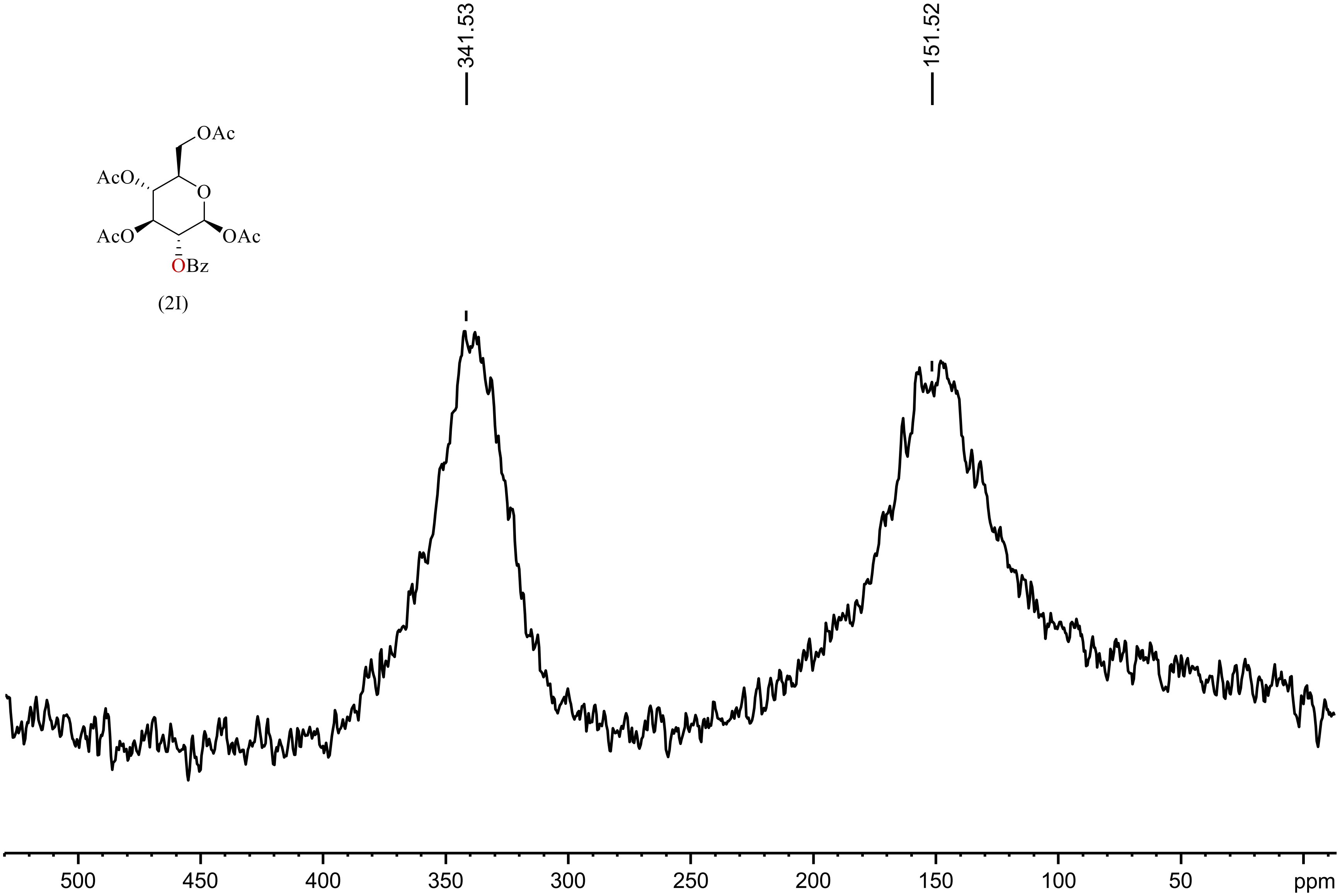

Figure S11. ^{17}O NMR of 1,3,4,6-Tetra-O-acetyl, [2-^{17}O]-benzoyl-beta-D-mannopyranose **(**2I**)** in $CDCl_3$ at 9.4 T.

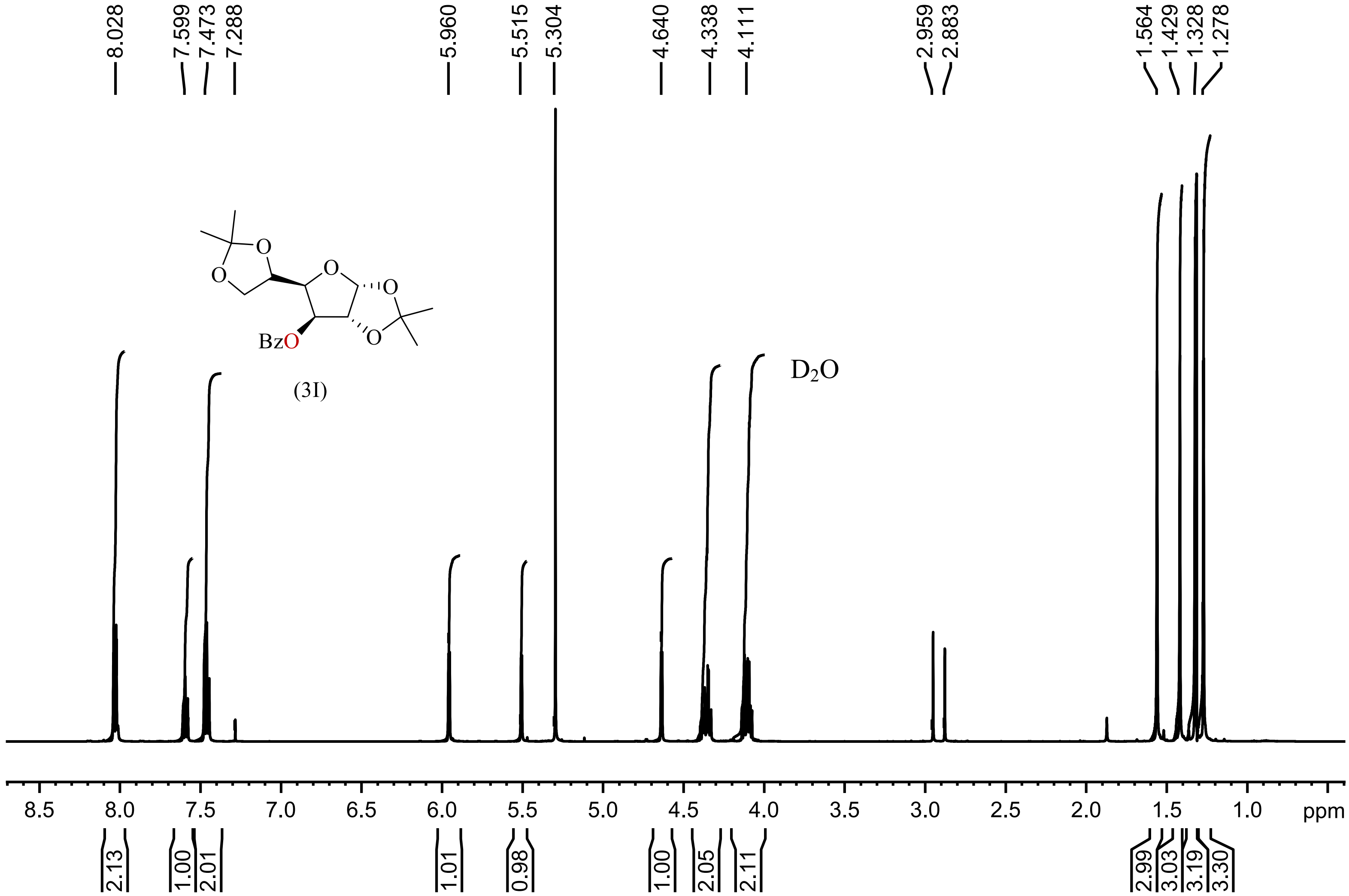

Figure S12. ^{1}H NMR of [3-^{17}O]-Benzoyl-1, 2-O-isopropylidene-α-D-glucofuranose (3I) in $CDCl_3$ at 11.7 T.

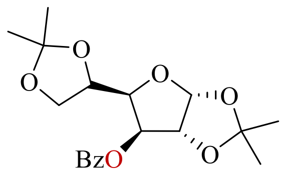

(3I)

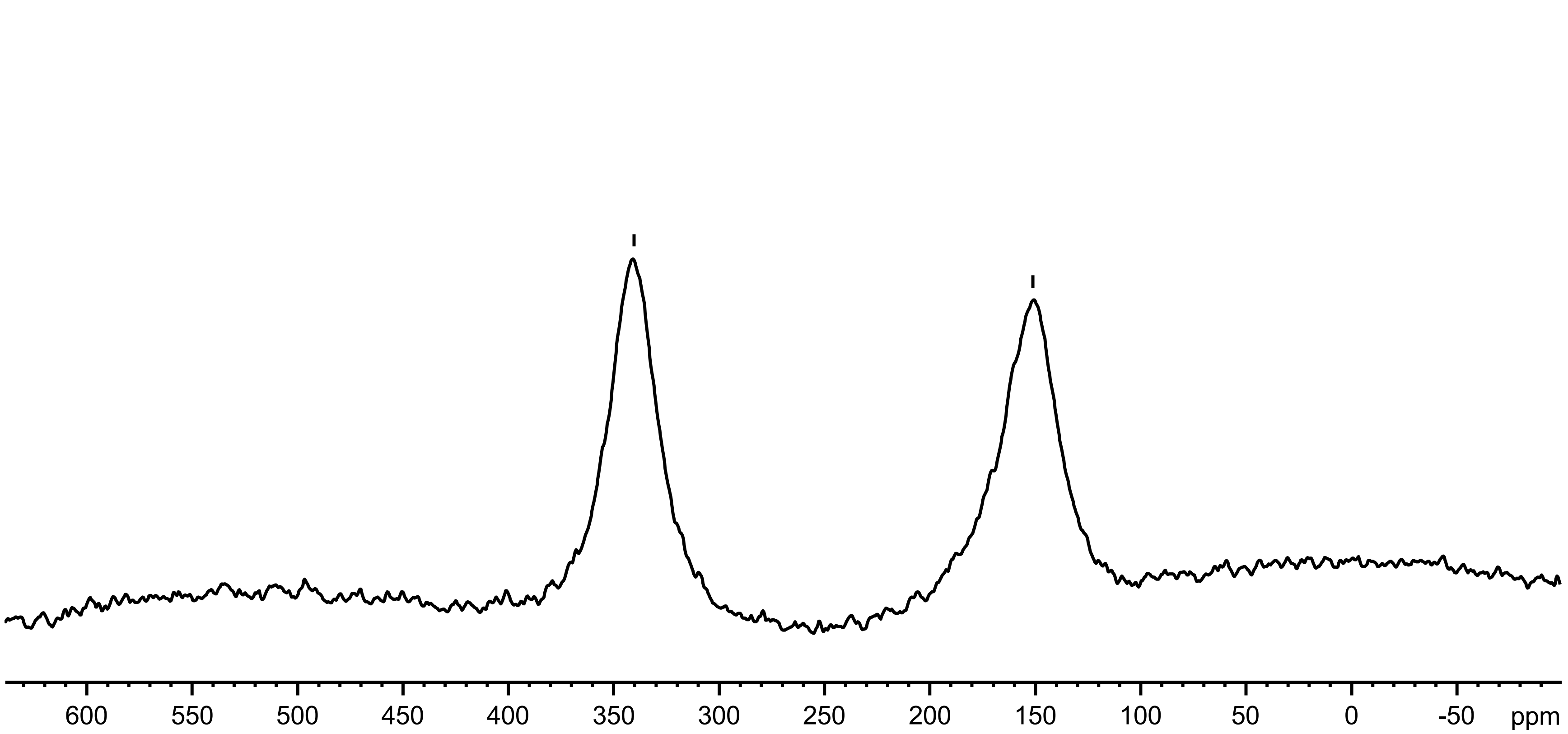

Figure S13. ^{17}O NMR of [3-^{17}O]-Benzoyl-1, 2-O-isopropylidene-α-D-glucofuranose (3I) in $CDCl_3$ at 9.4 T.

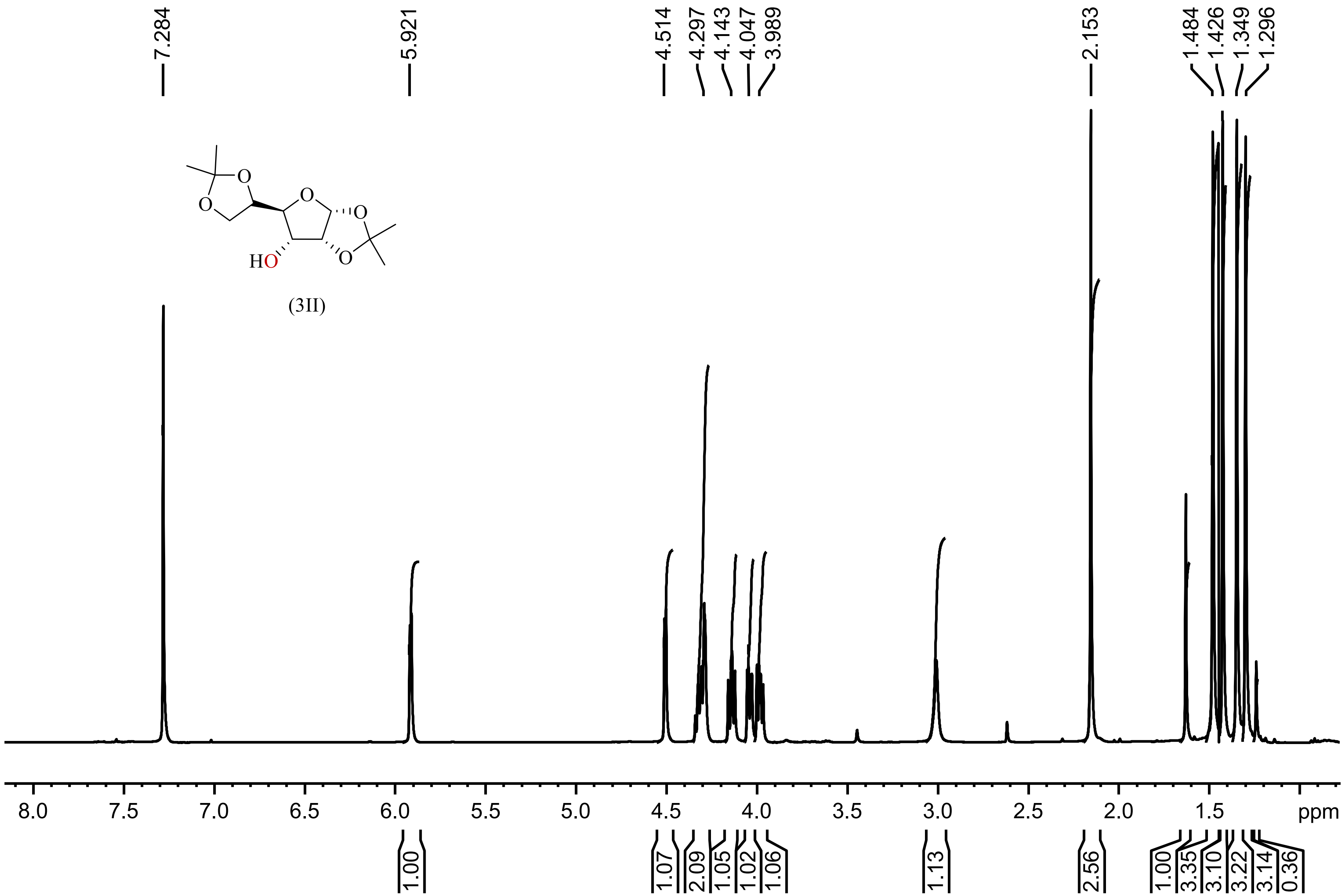

Figure S14. ^{1}H NMR of [3-^{17}O]-1,2;5,6-Di-O-isopropylidene-α-D-glucofuranose (3II) in $CDCl_3$ in 9.4 T.

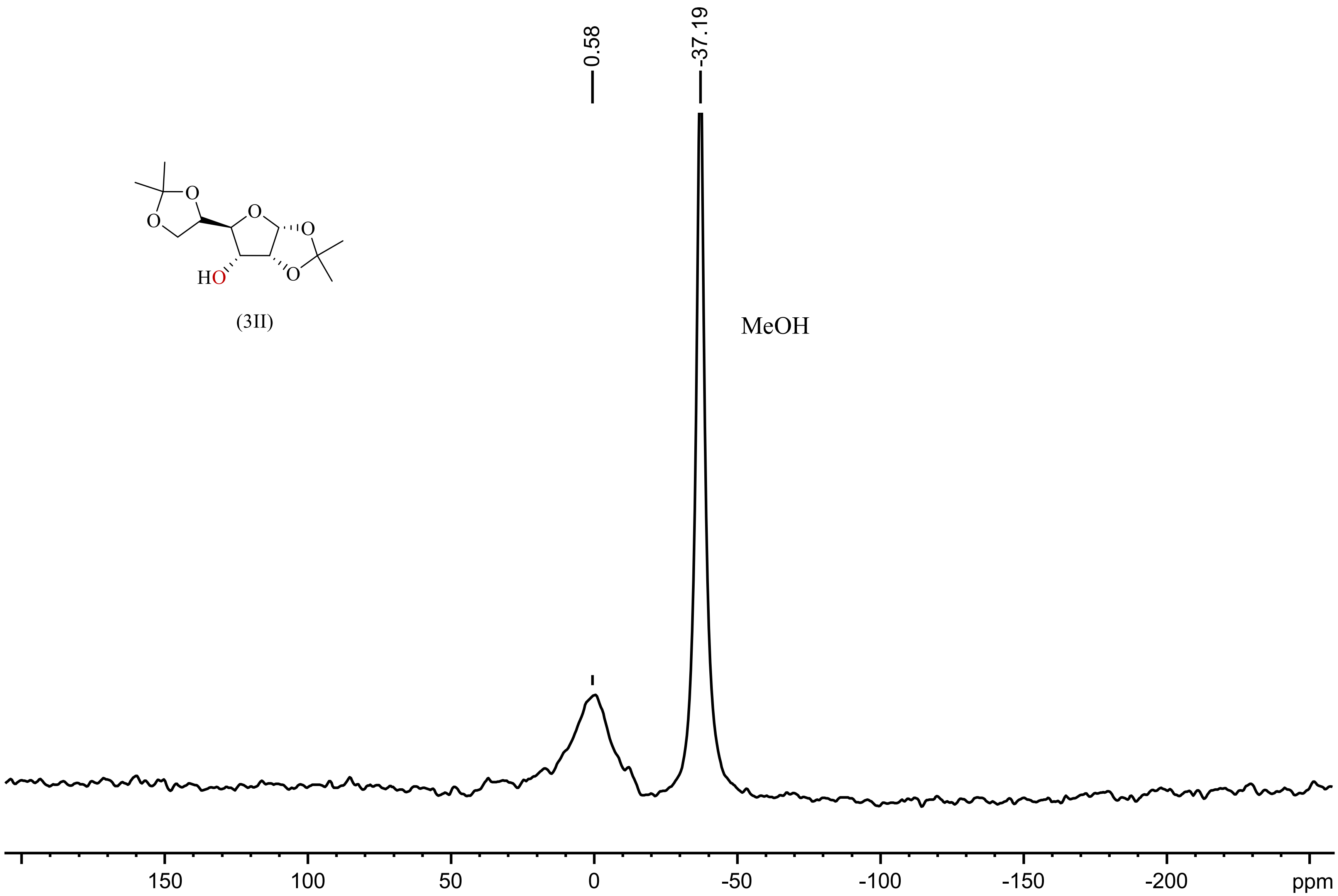

Figure S15. ^{17}O NMR of [3-^{17}O]-1,2;5,6-Di-O-isopropylidene-α-D-glucofuranose (3II) in $CDCl_3$ at 9.4 T.

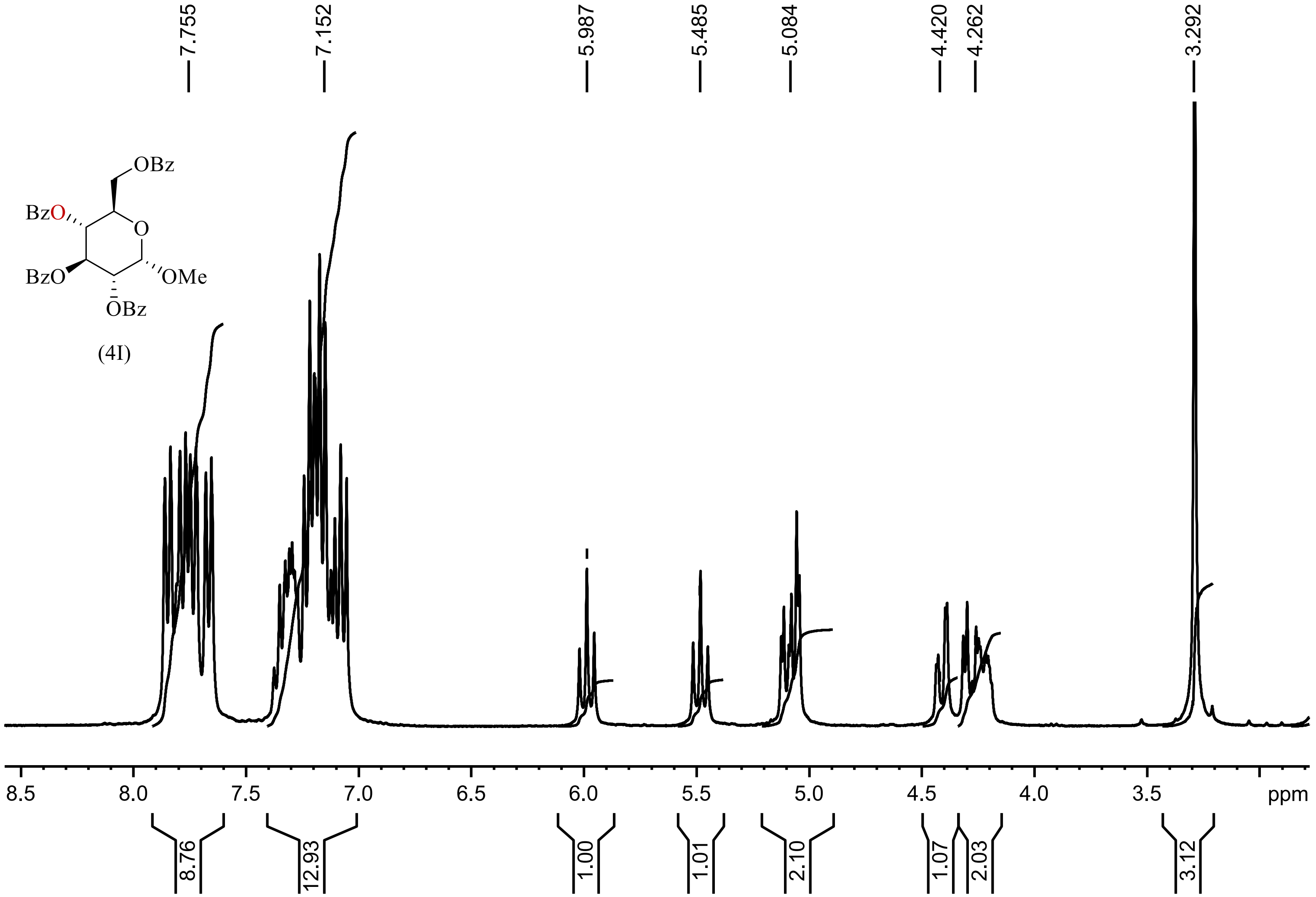

Figure S16. ^{1}H NMR of 1-Methyl-2, 3, [4-^{17}O], 6-tetra-O-benzoyl-α-D-glucopyranoside (4I) in $CDCl_3$ at 7.1 T.

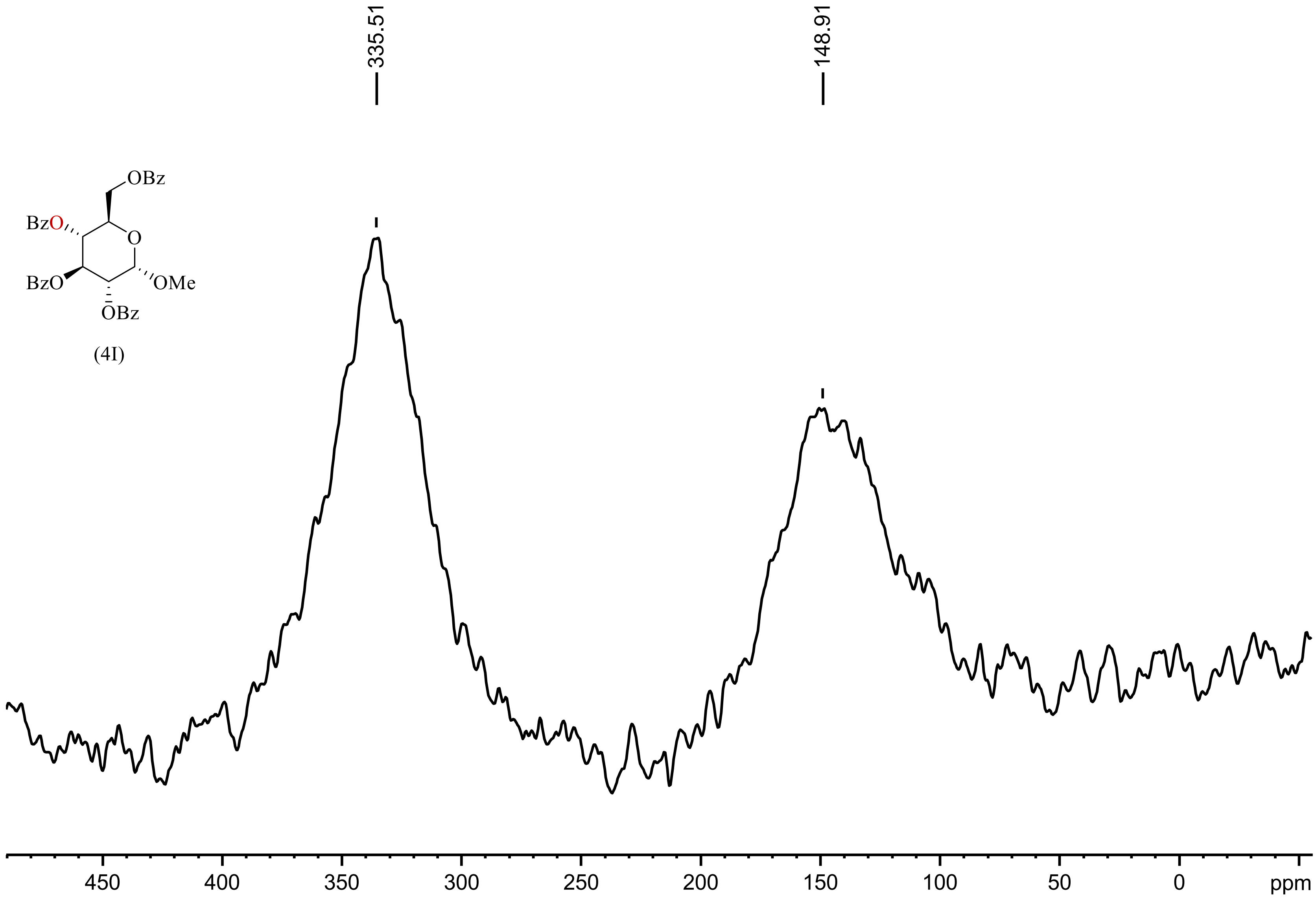

Figure S17. ^{17}O NMR of 1-Methyl-2, 3, [4-^{17}O], 6-tetra-O-benzoyl-α-D-glucopyranoside (4I) in $CDCl_3$ at 11.7 T.

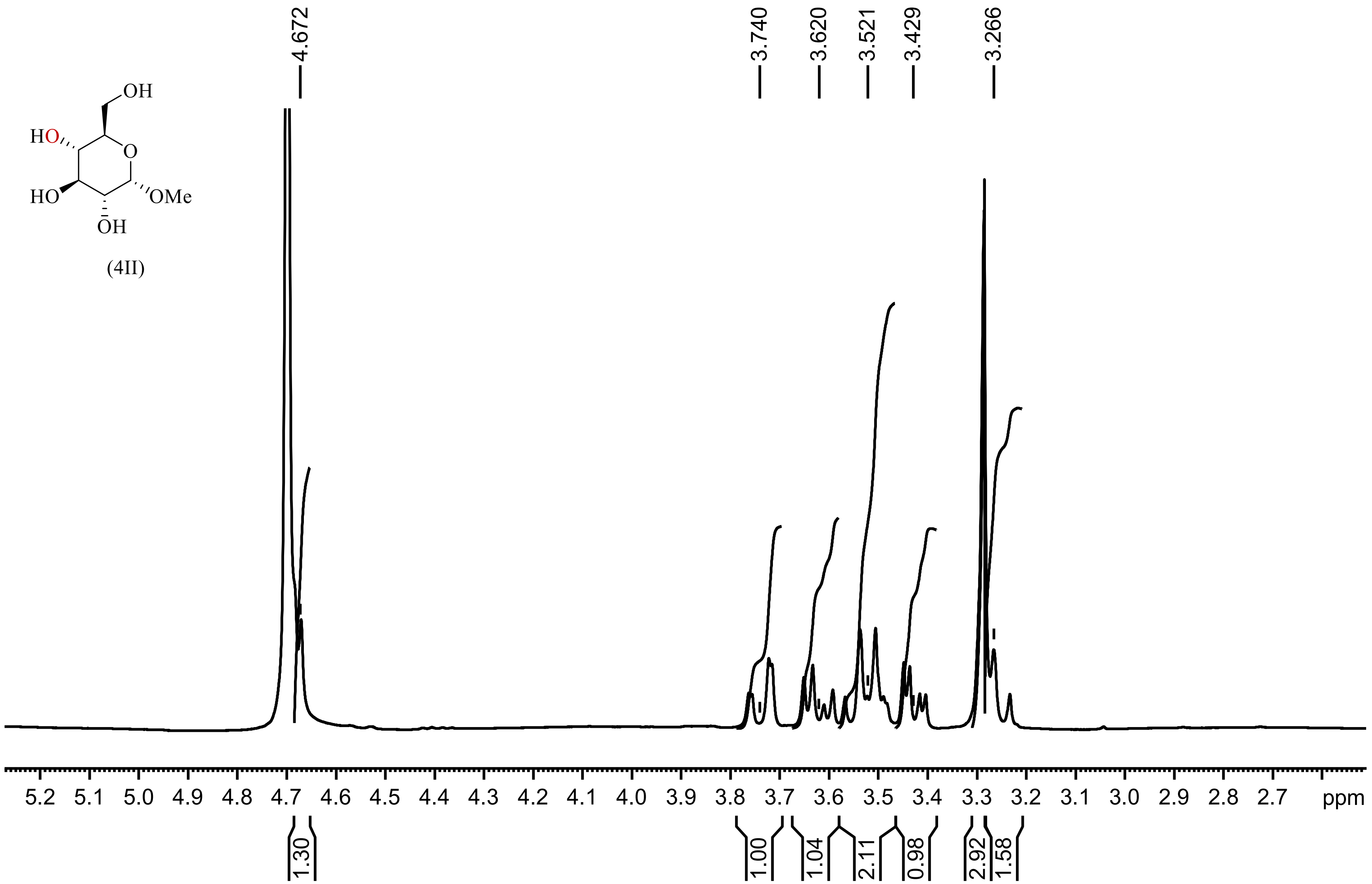

Figure S18. ^{1}H NMR of [4-^{17}O]-1-Methyl-α-D-glucopyranoside (4II) in D_2O at 9.4 T.

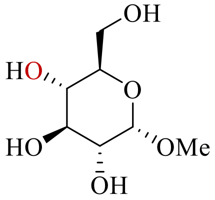

(4II)

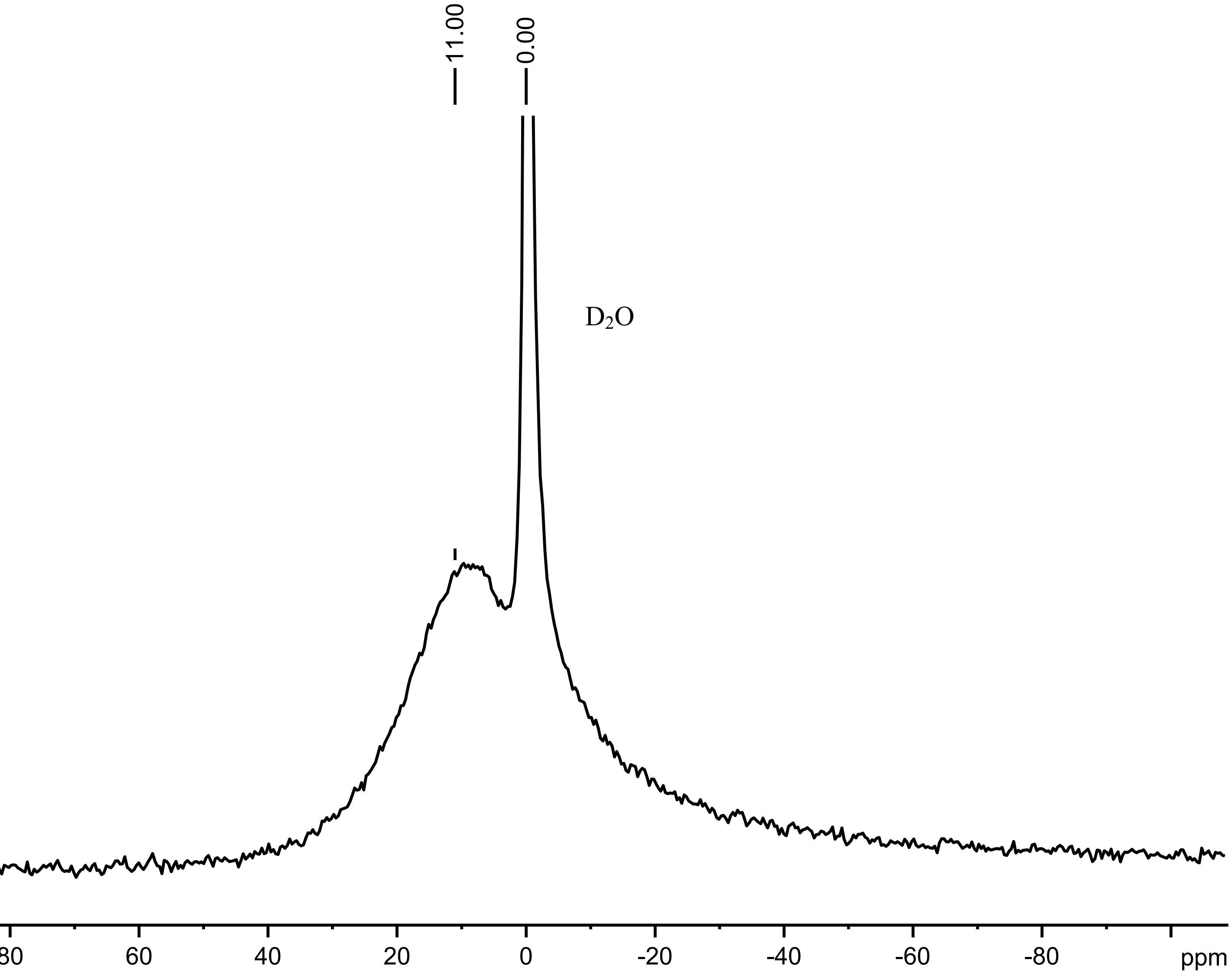

Figure S19. ^{17}O NMR of [4-^{17}O]-1-Methyl-α-D-glucopyranoside (4II) in D_2O at 11.7 T.

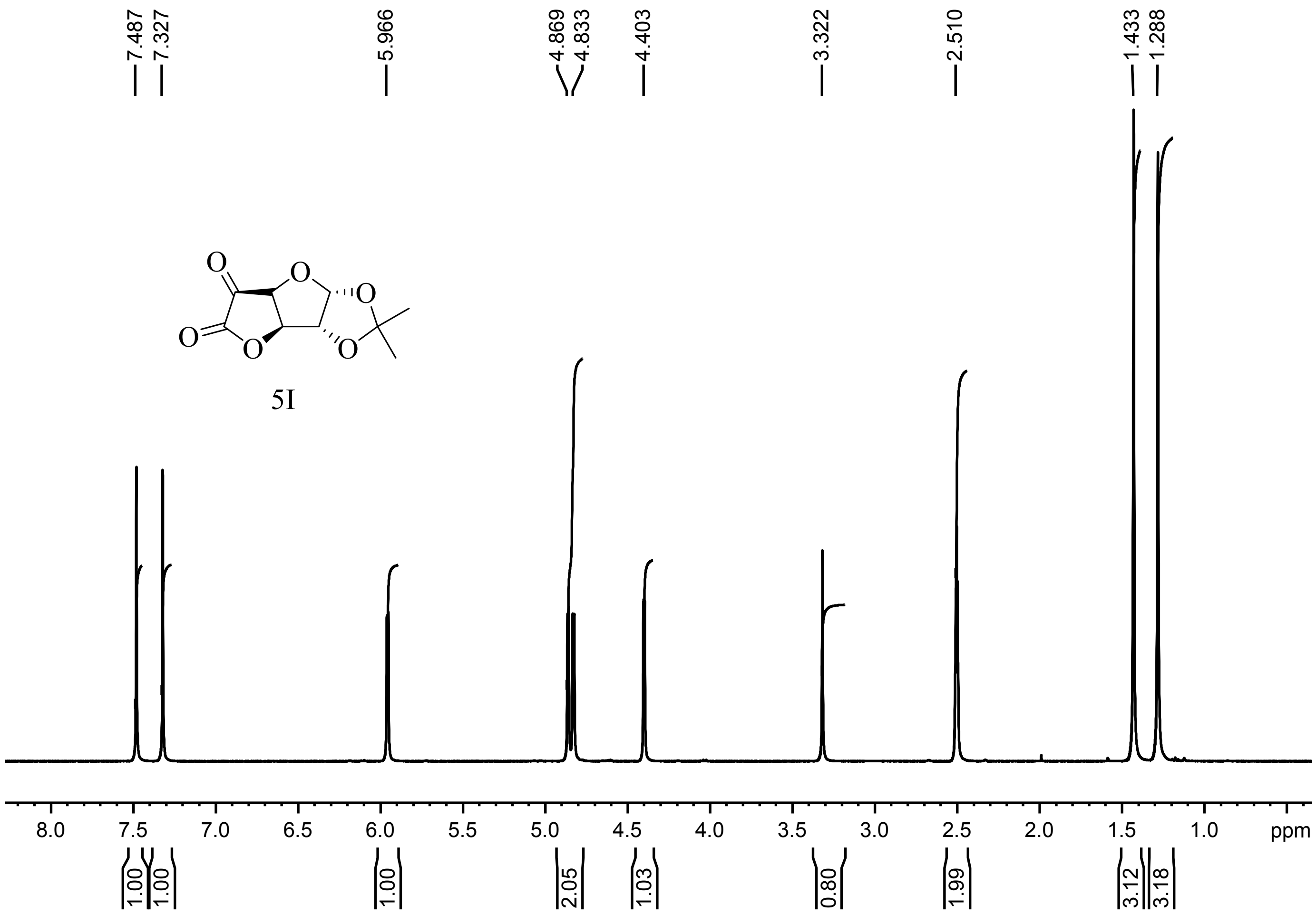

Figure S20. ^{1}H NMR spectrum of 1, 2-O-isopropylidene-D-xylo-hexofuranurono-6, 3-lactone-5-ulose hydrate (5I) in DMSO-d_6 at 9.4 T.

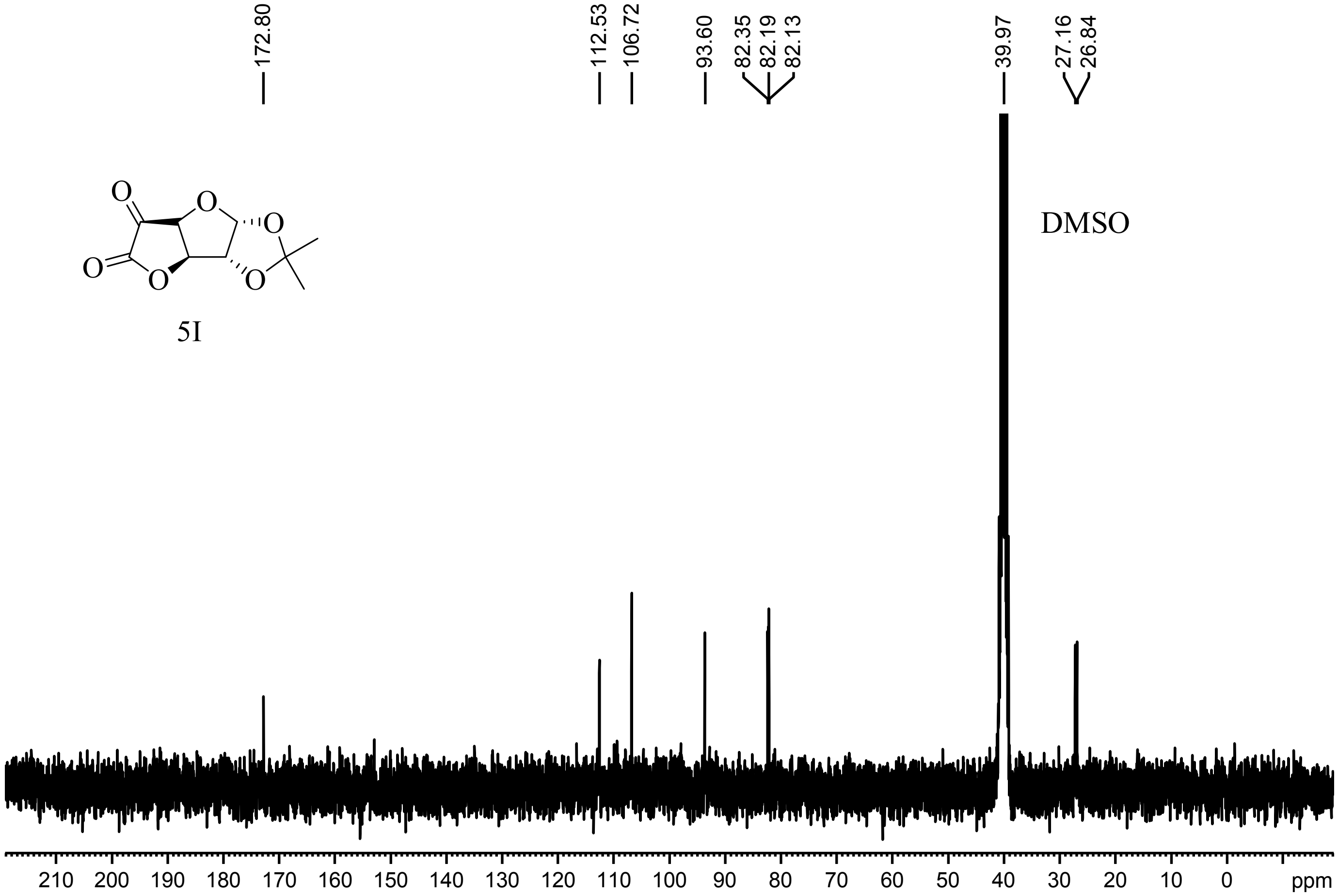

Figure S21. ^{13}C NMR spectrum of 1, 2-O-isopropylidene-D-xylo-hexofuranurono-6, 3-lactone-5-ulose hydrate (5I) in DMSO-d_6 at 7.1 T.

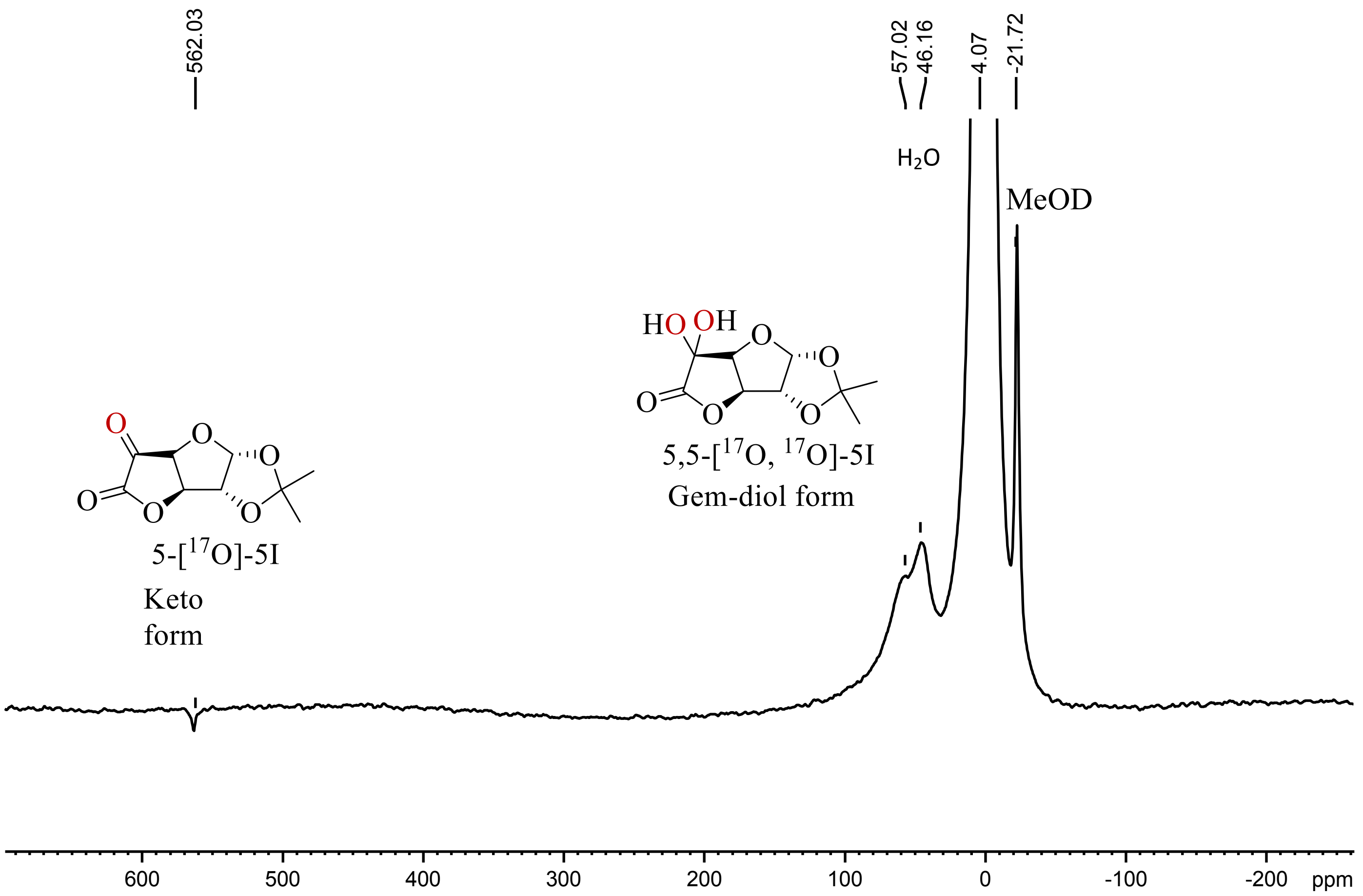

Figure S22. ^{17}O NMR spectrum of [5,5-$^{17}O_2$]-1, 2-O-isopropylidene-D-xylo-hexofuranurono-6, 3-lactone-5-ulose hydrate (5I) in methanol-d_4 at 9.4 T.

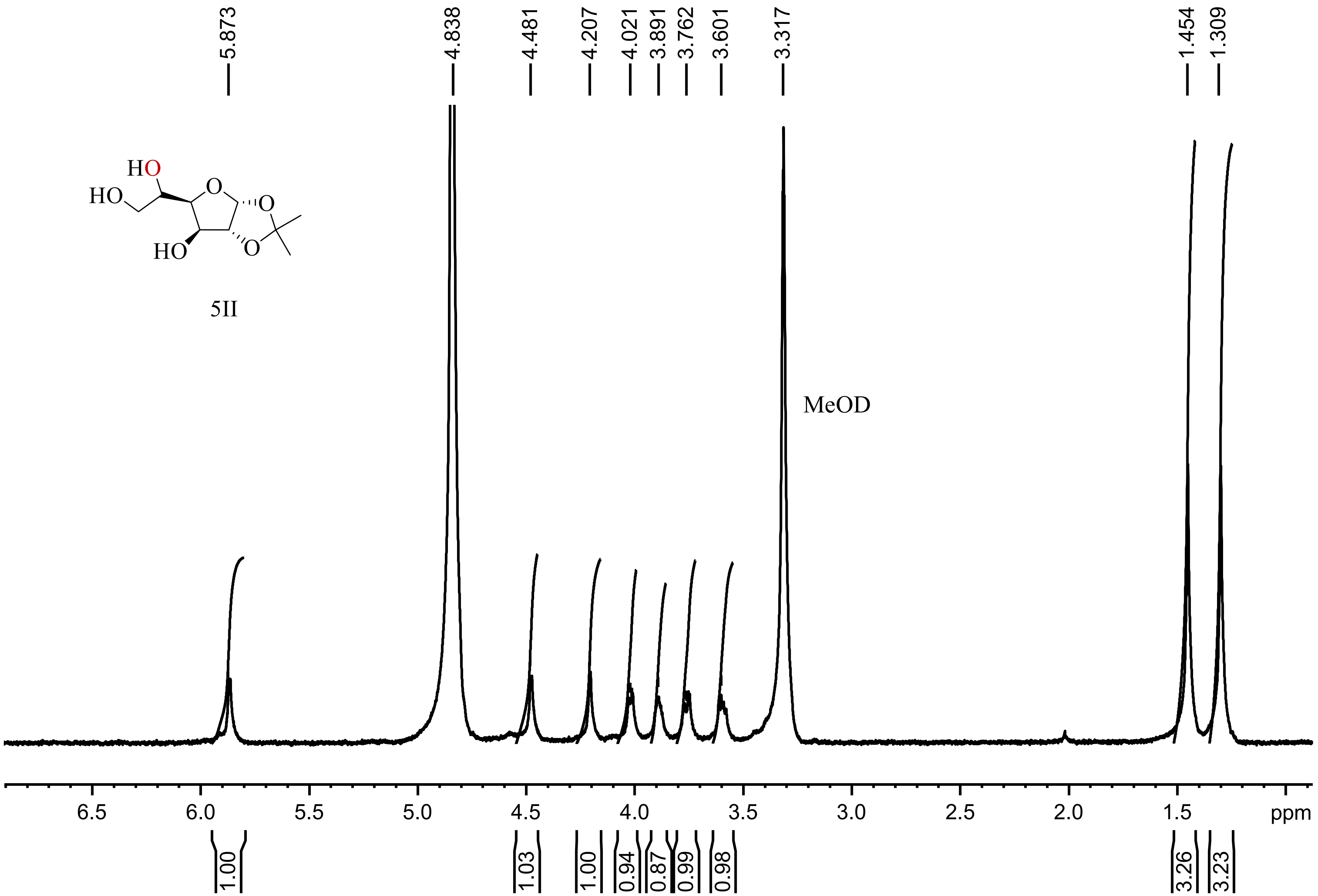

Figure S23. ^{1}H NMR of 1, 2-O-isopropylidene-α-D-[5-^{17}O]-glucofuranose (5II) in methanol-d_4 at 11.7 T.

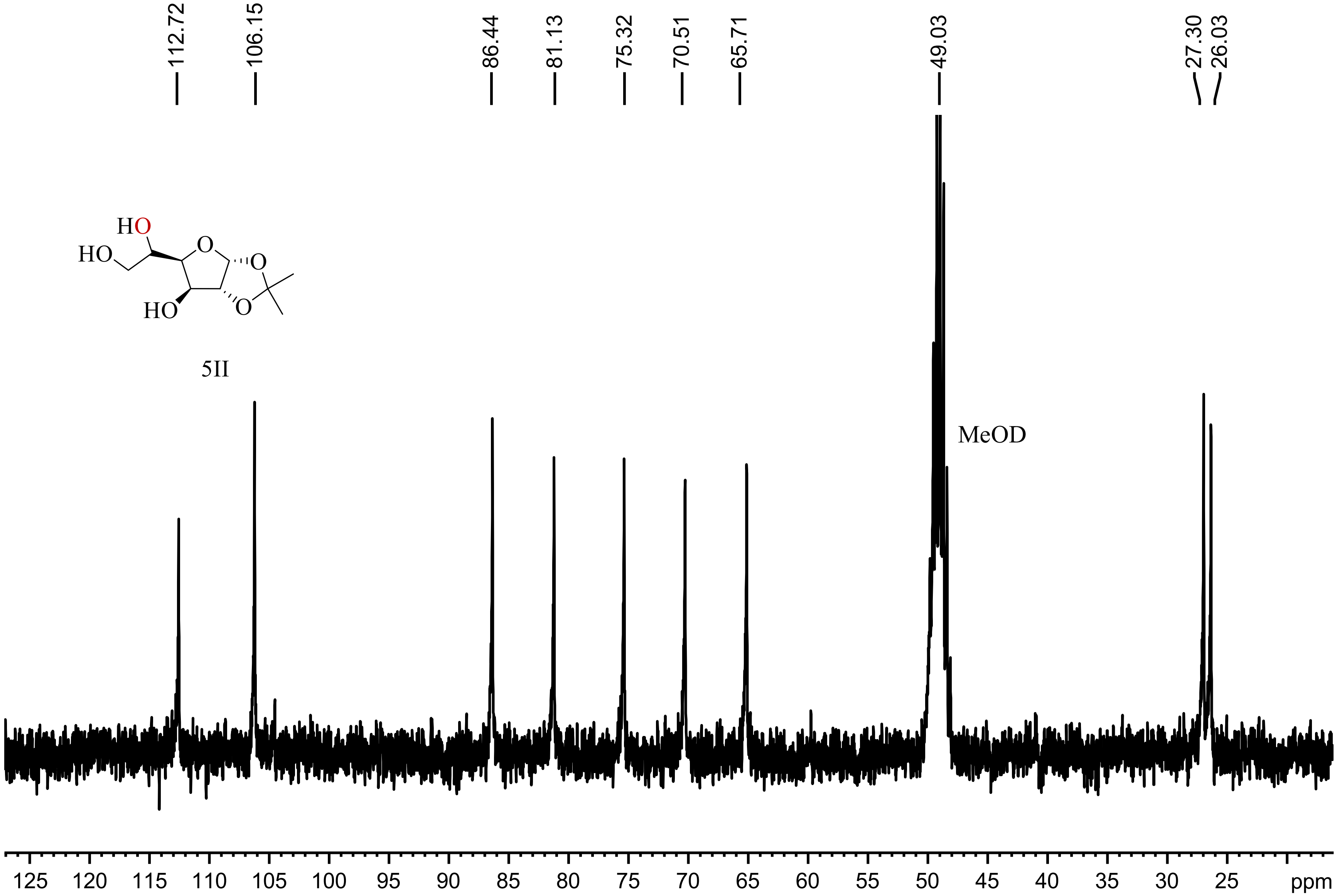

Figure S24. ^{13}C NMR of 1, 2-O-isopropylidene-α-D-[5-^{17}O]-glucofuranose (5II) in methanol-d_4 at 7.1 T.

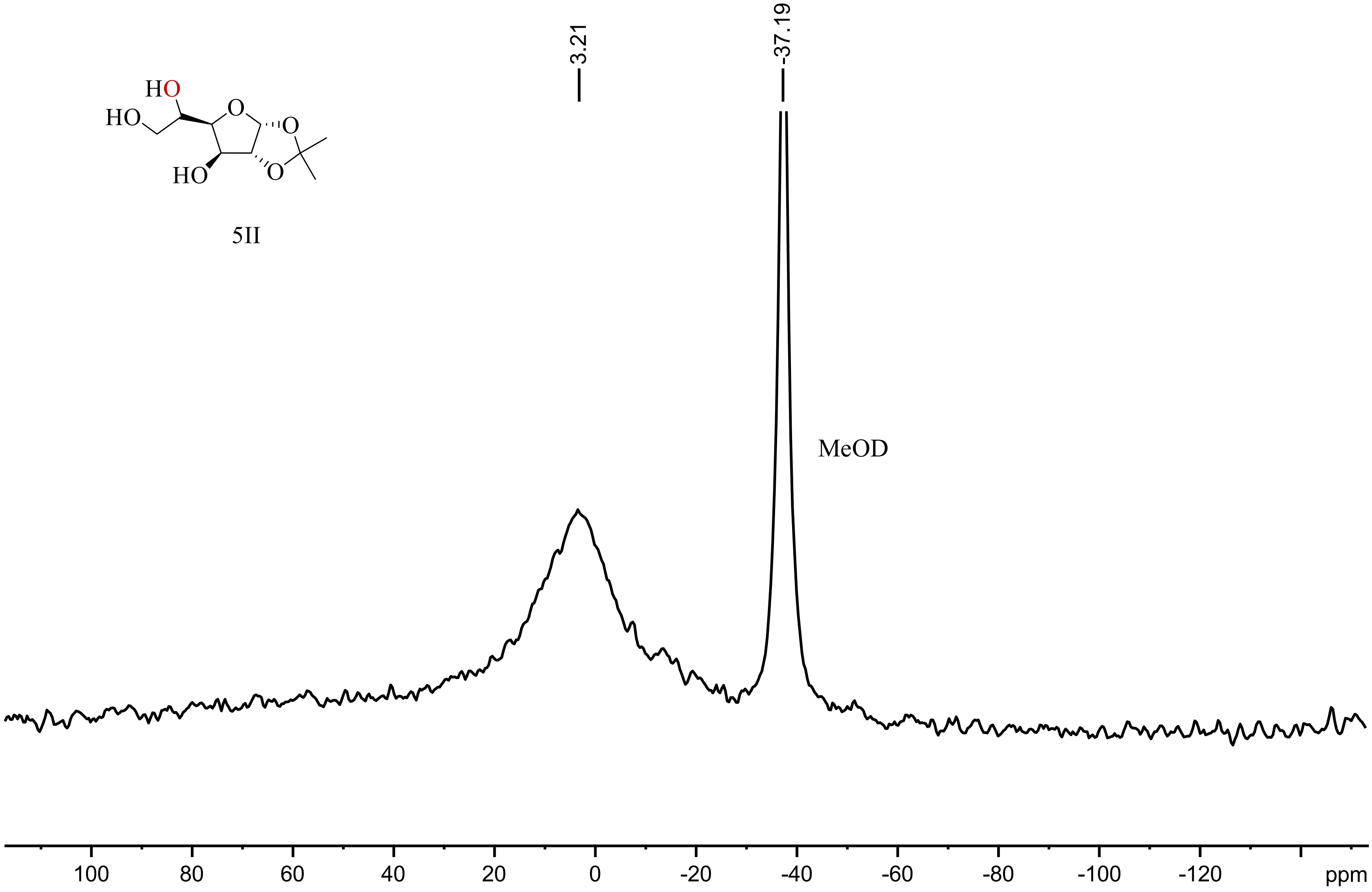

Figure S25. ^{17}O NMR spectrum of 1, 2-O-isopropylidene-α-D-[5-^{17}O]-glucofuranose (5II) in methanol-d_4 at 11.7 T.

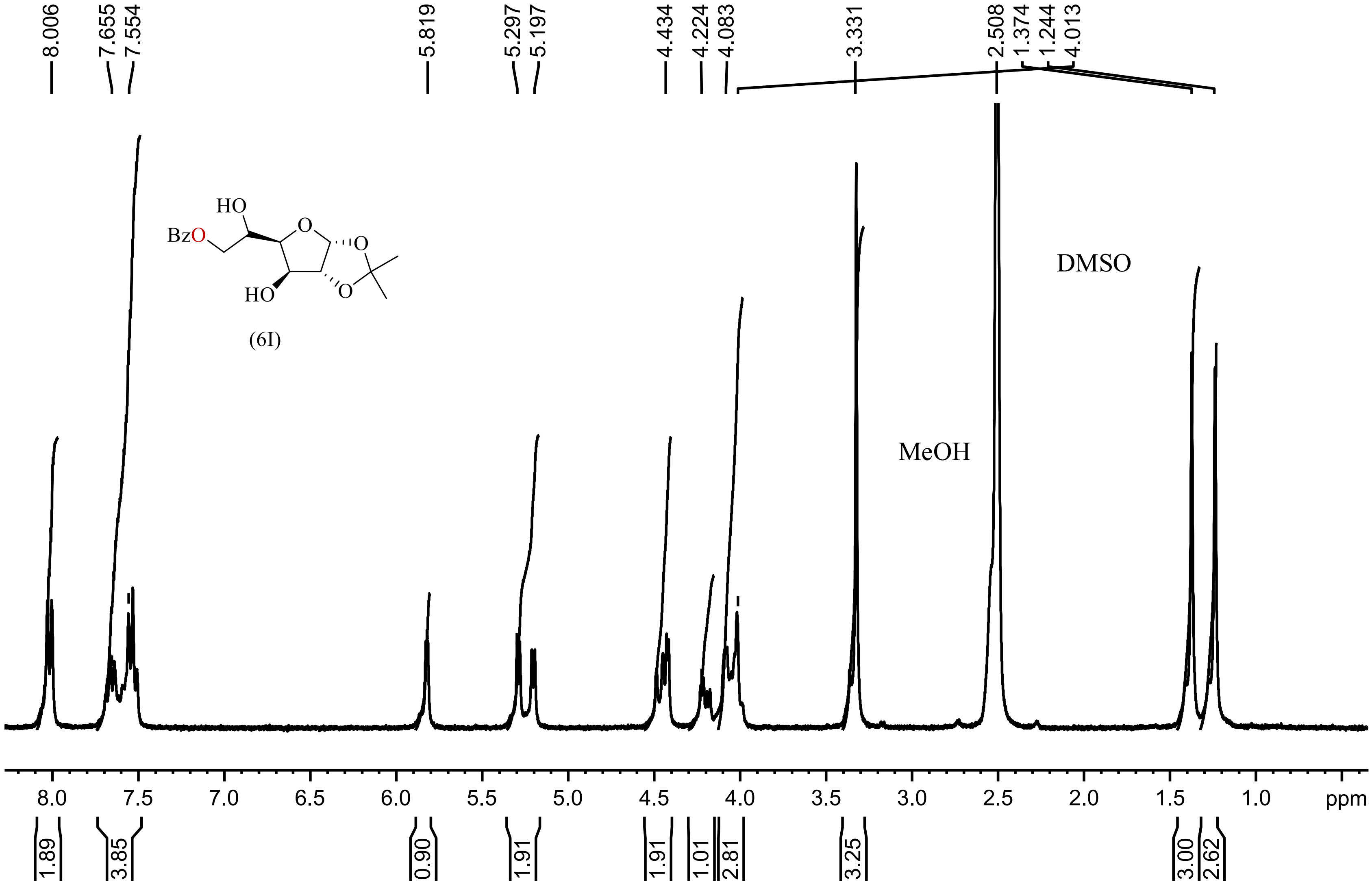

Figure S26. ^{1}H NMR of [6-^{17}O]-Benzoyl-1, 2-O-isopropylidene-α-D-glucofuranose (6I) in DMSO-d_6 at 7.1 T.

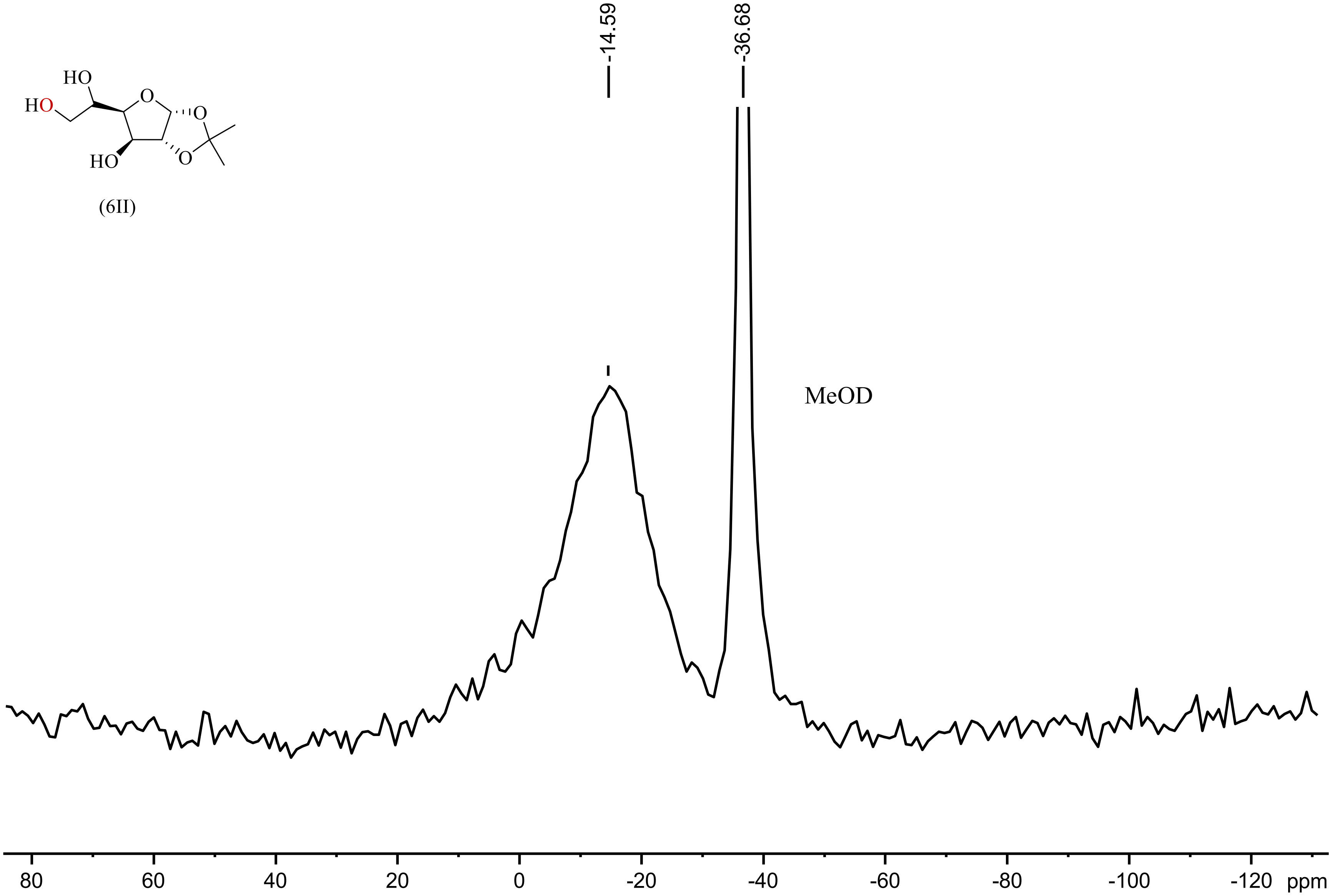

Figure S27. ^{17}O NMR of 1, 2-O-Isopropylidene-α-D-[6-^{17}O] glucofuranose (6II) in Methonal-d_4 at 9.4 T.
